# Supplementary material for: Accessibility to Peptidoglycan Is Important for the Recognition of Gram-Positive Bacteria in Drosophila
Source: Cell Rep. 2019 May 21;27(8):2480–2492.e6. doi: 10.1016/j.celrep.2019.04.103 (PMC6533200; doi:10.1016/j.celrep.2019.04.103)
Supplement: Document S1. Figures S1–S8 and Tables S1–S4 [file mmc1.pdf]

Cell Reports, Volume *27*

Supplemental Information

Accessibility to Peptidoglycan Is Important for the Recognition of Gram-Positive Bacteria in *Drosophila*

Filipa Vaz, Ilias Kounatidis, Gonçalo Covas, Richard M. Parton, Maria Harkiolaki, Ilan Davis, Sergio Raposo Filipe, and Petros Ligoxygakis

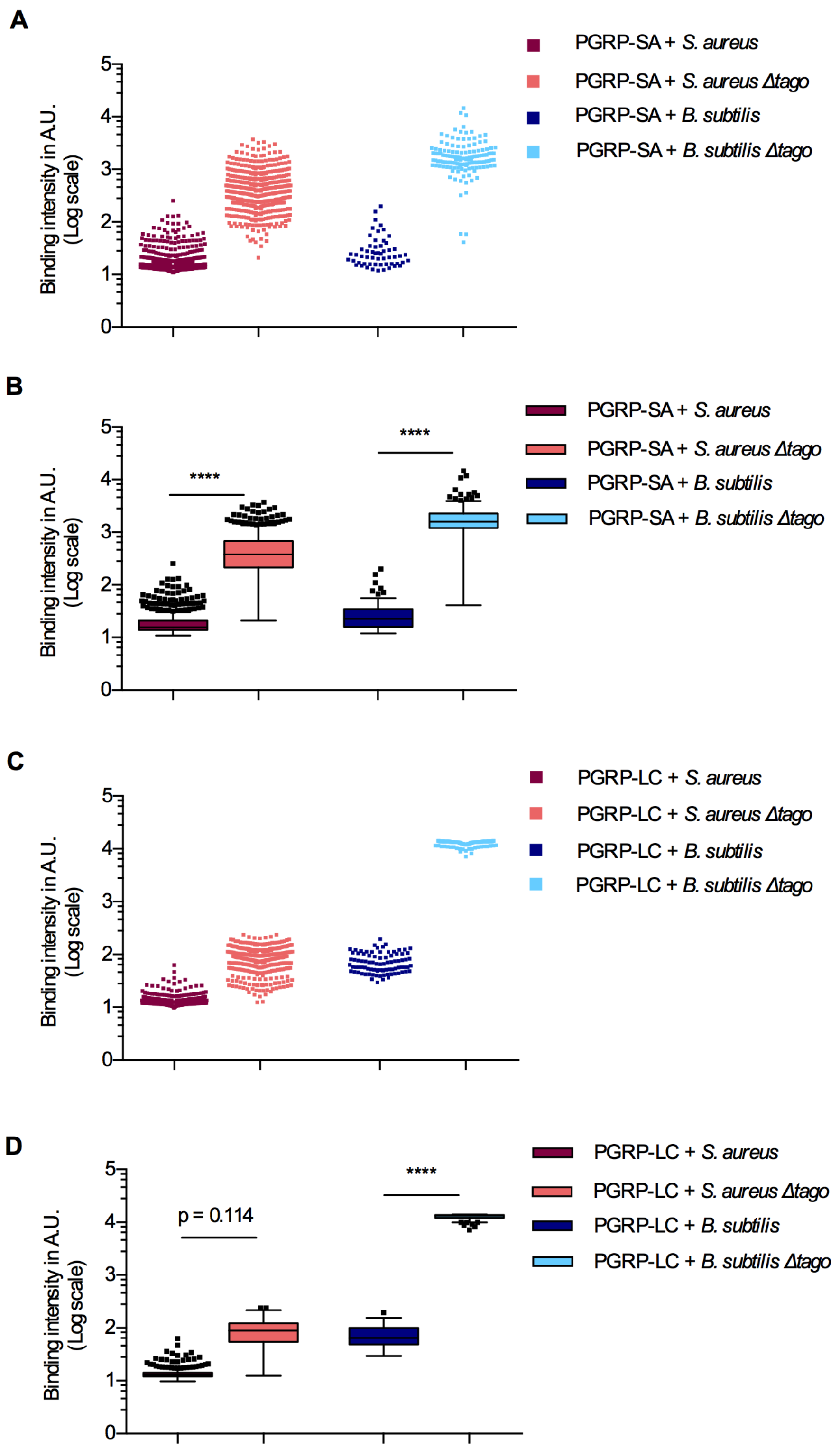
A
Binding intensity in A.U. (Log scale)
0
1
2
3
4
5
PGRP-SA + S. aureus
PGRP-SA + S. aureus Δtago
PGRP-SA + B. subtilis
PGRP-SA + B. subtilis Δtago
B
****
****
C
PGRP-LC + S. aureus
PGRP-LC + S. aureus Δtago
PGRP-LC + B. subtilis
PGRP-LC + B. subtilis Δtago
D
p = 0.114
****

Figure S1. Quantification of PGRP-SA and PGRP-LC binding to *S. aureus, B. bacillus* and their TagO variants. Related to Figures 1 and 2 (A) Scatterplot to indicate all values of mCherry-PGRP-SA binding to whole bacteria **(B)** Box-plot with mean values and 95% confidence intervals; all comparisons with one-way ANOVA (see Table S1). **(C)** Scatterplot to indicate all values of mCherry-PGRP-LCx binding to whole bacteria **(D)** Box-plot with mean values and 95% confidence intervals; all comparisons with one-way ANOVA (see Table S1).

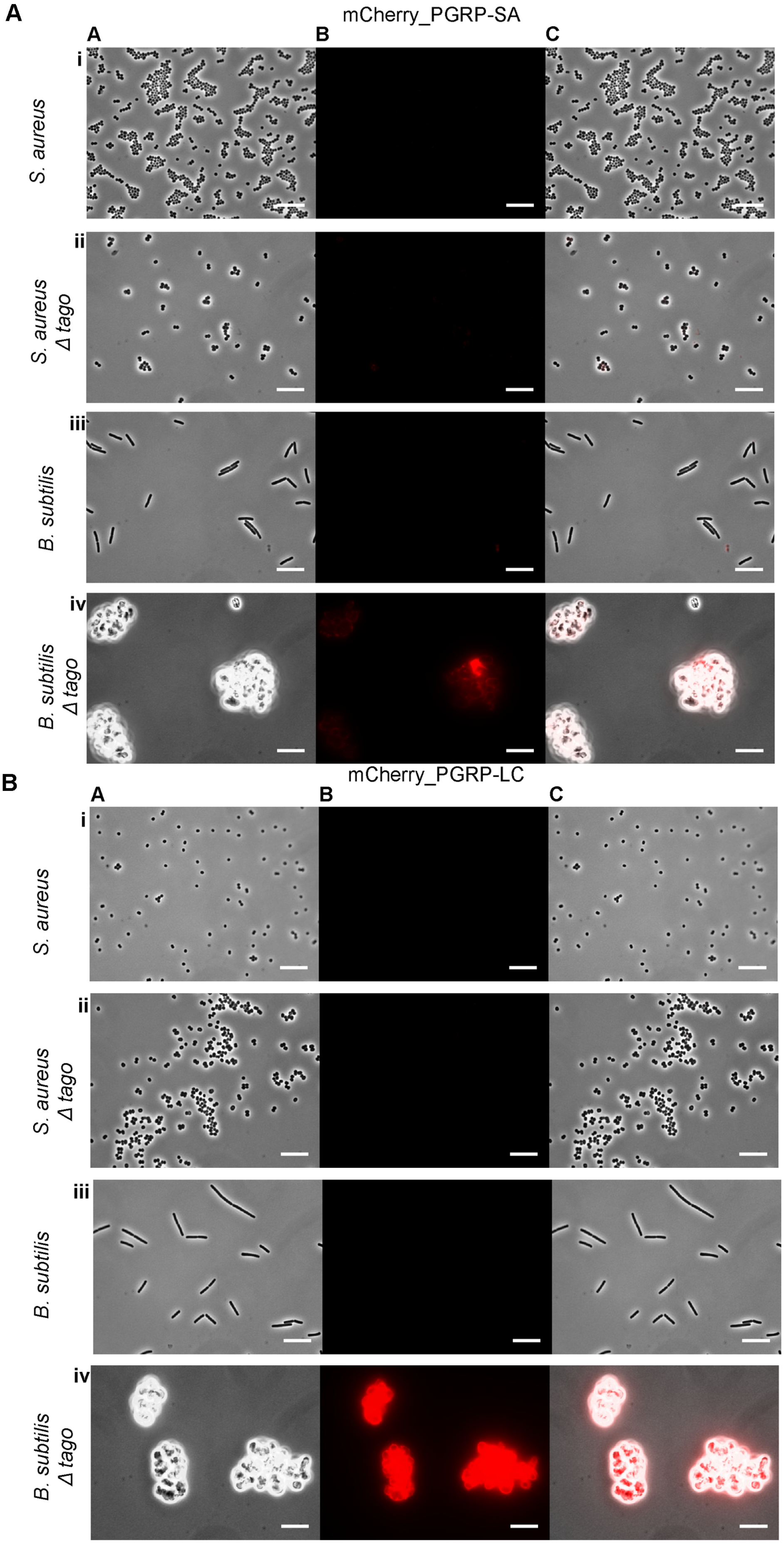

A
mCherry_PGRP-SA
A
B
C
i
S. aureus
ii
S. aureus Δ tago
iii
B. subtilis
iv
B. subtilis Δ tago
B
mCherry_PGRP-LC
A
B
C
i
S. aureus
ii
S. aureus Δ tago
iii
B. subtilis
iv
B. subtilis Δ tago

Figure S2. Binding of mCherry-PGRP-SA (A) and mPGRP-LC (B) to *S. aureus, B. bacillus* and their TagO mutants with contrast adjusted for easier visual comparison of images with low signal intensities. Related to Figures 1 and 2. (A) Phase contrast **(B)** m-Cherry-PGRP-SA and **(C)** overlay

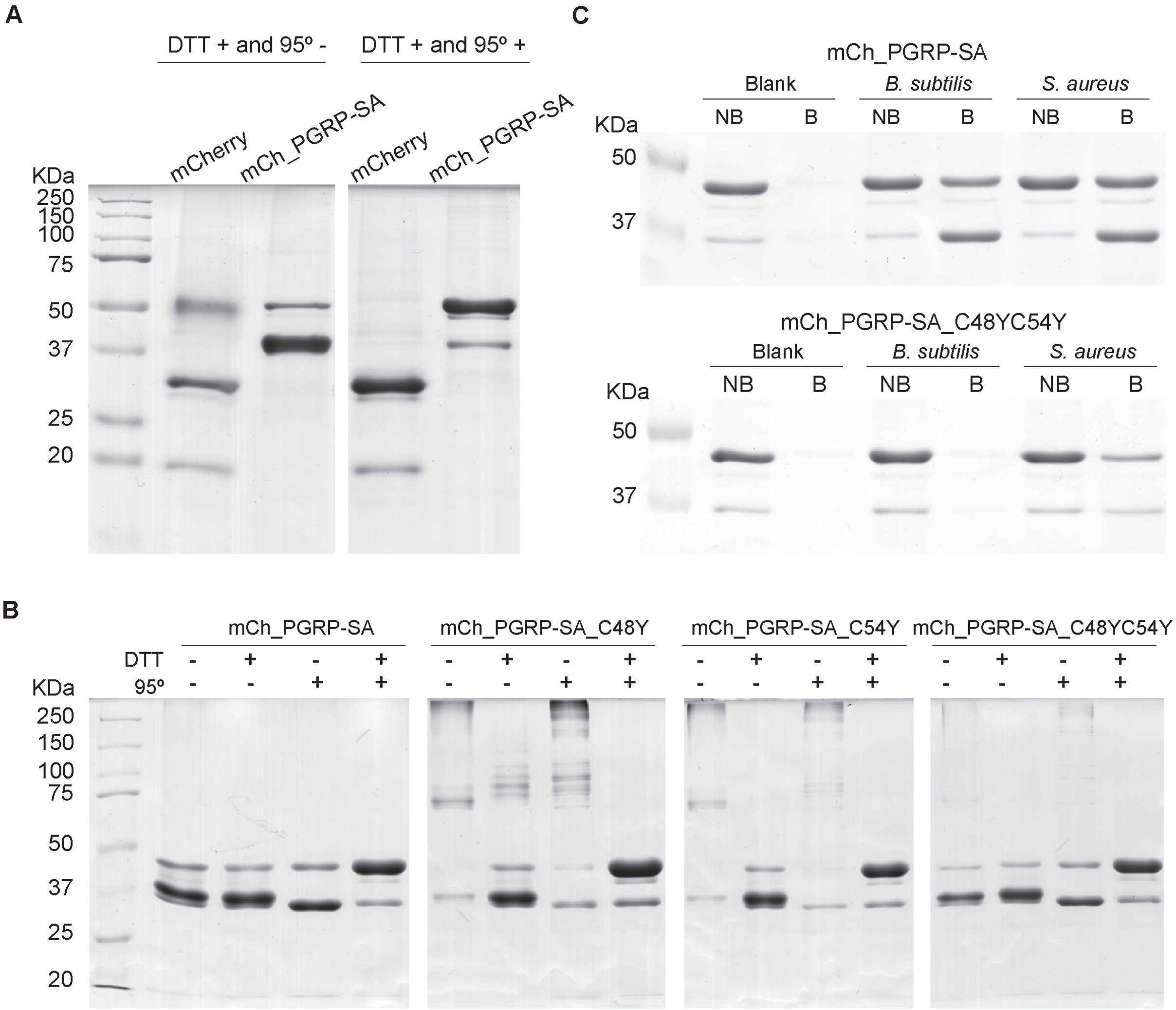
A
DTT + and 95º -
DTT + and 95º +
mCherry
mCh_PGRP-SA
mCherry
mCh_PGRP-SA
KDa
250
150
100
75
50
37
25
20
C
mCh_PGRP-SA
Blank
B. subtilis
S. aureus
KDa
NB
B
NB
B
NB
B
50
37
mCh_PGRP-SA_C48YC54Y
Blank
B. subtilis
S. aureus
KDa
NB
B
NB
B
NB
B
50
37
B
mCh_PGRP-SA
mCh_PGRP-SA_C48Y
mCh_PGRP-SA_C54Y
mCh_PGRP-SA_C48YC54Y
DTT
95º
KDa
250
150
100
75
50
37
25
20

Figure S3. Purified recombinant mCherry_PRGP-SA proteins. Related to Figures 1, 2 and 4. (A) Purified mCherry has an expected size of 29.070 KDa. Under non-fully denaturing conditions (DTT but not heated), mCherry presented a multimer form (ca. 50 KDa), the fully denatured 29 KDa band and a band below due to an internal RBS, which led to a 12aa shorter protein. The lower band, ca. 20 KDa is a degradation product. mCherry_PGRP-SA submitted to the same treatment, presented two bands ca. 50 KDa, which corresponded to the full-length denatured protein (49.701 KDa) and to the 12aa shorter form. The band of ca. 40 KDa is the non-fully denatured form. When heated at 95°, both proteins showed a stronger band with the expected sizes for the full-length proteins. **(B)** The single mutants C48Y and C54Y had an expected size of 49.77 KDa, while C48YC54Y a size of 49.81 KDa. Both single cysteine mutants presented high number of multimer forms, which suggested that they had folding defects. In contrast, C48YC54Y presented in solution, a similar native conformation as the wild-type protein. **(C)** Purified and quantified PG from *B. subtilis* and *S. aureus* was incubated with mCherry_PGRP-SA and mCherry_PGRP-SA^{C48YC54Y}. After incubation, the complex PG-PGRP was pulled down by low speed centrifugation. NB – Non-bound fraction, i.e. amount of PGRP that did not bind to PG, present in the supernatant after centrifugation. B – Bound fraction, i.e. amount of PGRP that bound to PG and was pulled down after centrifugation (pellet). Blank shows each PGRP incubated in the reaction buffer without PG under the same conditions as the PG-PGRP sample. Both mCherry_PGRP-SA and mCherry_PGRP-SA^{C48YC54Y} were soluble and did not precipitate. The double cysteine mutant however, showed impaired binding ability to both PG types.

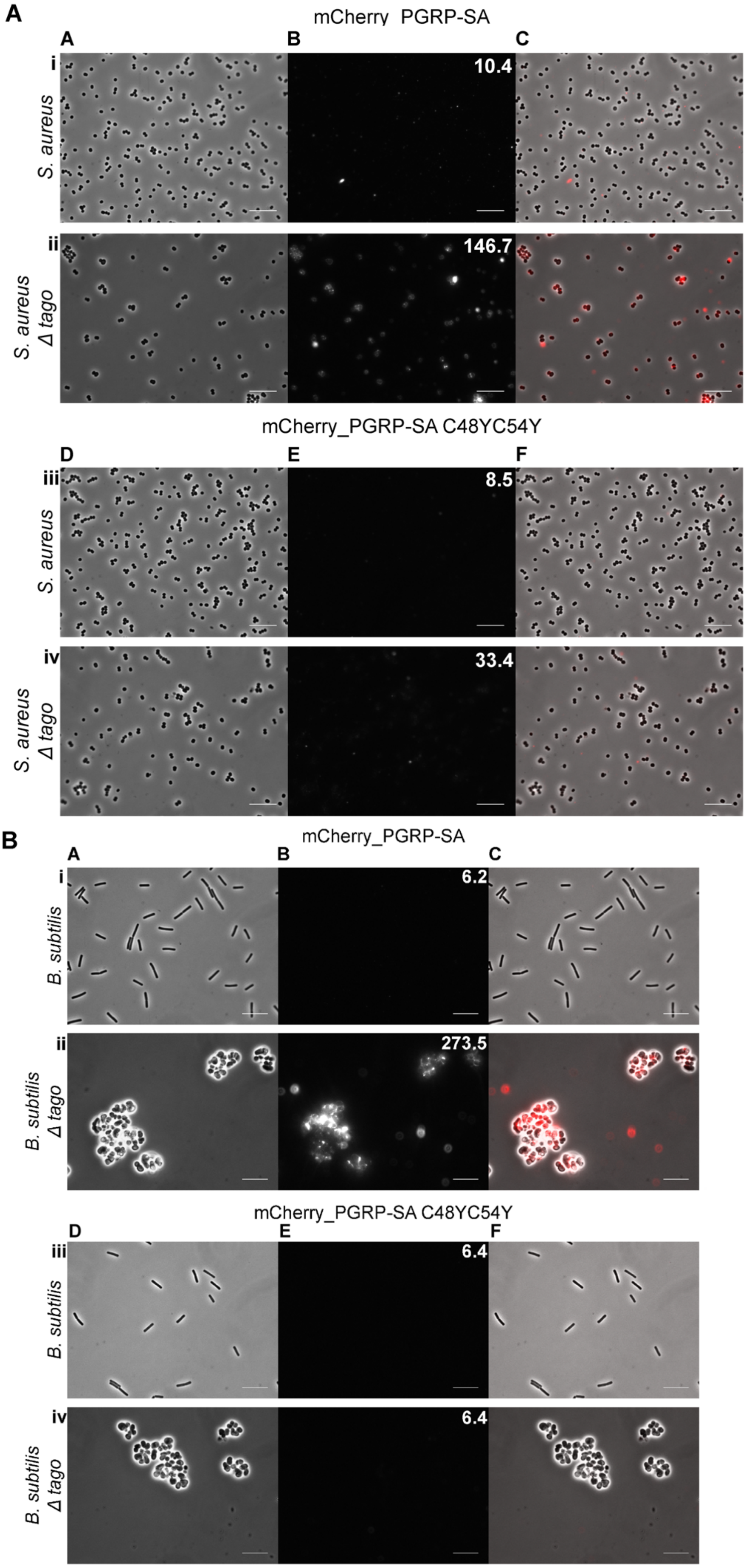
A
mCherry PGRP-SA
A
B
C
i
S. aureus
10.4
ii
S. aureus Δ tago
146.7
mCherry_PGRP-SA C48YC54Y
D
E
F
iii
S. aureus
8.5
iv
S. aureus Δ tago
33.4
B
mCherry_PGRP-SA
A
B
C
i
B. subtilis
6.2
ii
B. subtilis Δ tago
273.5
mCherry_PGRP-SA C48YC54Y
D
E
F
iii
B. subtilis
6.4
iv
B. subtilis Δ tago
6.4

Figure S4. Binding of m-Cherry-PGRP-SA and m-Cherry-PGRP-SA^{C48YC54Y} to *S. aureus* (A) and to *B. subtilis* (B). Related to Figures 1 and 2.

***S. aureus* wild type and *TagO* mutant*:* (A-C).** Binding of a wild type version of a fluorescently tagged PGRP-SA (mCherry_PGRP-SA) to *S. aureus* (i) and *S. aureusΔTagO* (ii). **(A)** Phase contrast channel **(B)** m-Cherry-PGRP-SA and **(C)** overlay. Quantification of binding showed a significant increase in median intensity from 10.4 AU (Bi) to 146.7 AU (Bii). **(D-F).** Binding of a fluorescently tagged mutant PGRP-SA (mCherry_PGRP-SA^{C48YC54Y}) to *S. aureus* (iii) and *S. aureusΔTagO* (iv). **(D)** Phase contrast channel **(E)** m-Cherry-PGRP-SA^{C48YC54Y} and **(F)** overlay. Quantification of binding showed a non-significant increase of median intensity from 8.5 AU (Eiii) to 33.4 AU (Eiv). Exposure time for all 10msec, scale bar 1μm.

***B. subtilis* wild type and *TagO* mutant (A-C).** Binding of a wild type version of a fluorescently tagged PGRP-SA (mCherry_PGRP-SA) to *B. subtilis* (i) and *B. subtilisΔTagO* (ii). **(A)** Phase contrast channel **(B)** m-Cherry-PGRP-SA and **(C)** overlay. Quantification of binding showed a significant increase in median intensity from 6.2 AU (Bi) to 273.5 AU (Bii). **(D-F).** Binding of a mutant version of a mutant fluorescently tagged PGRP-SA (mCherry_PGRP-SA^{C48YC54Y}) to *B. subtilis* (iii) and *B. subtilisΔTagO* (iv). **(D)** DIC channel **(E)** m-Cherry-PGRP-SA^{C48YC54Y} and **(F)** overlay. Quantification of binding showed no increase in median intensity (compare Eiii and Eiv). Exposure time for all 10ms, scale bar 1μm.

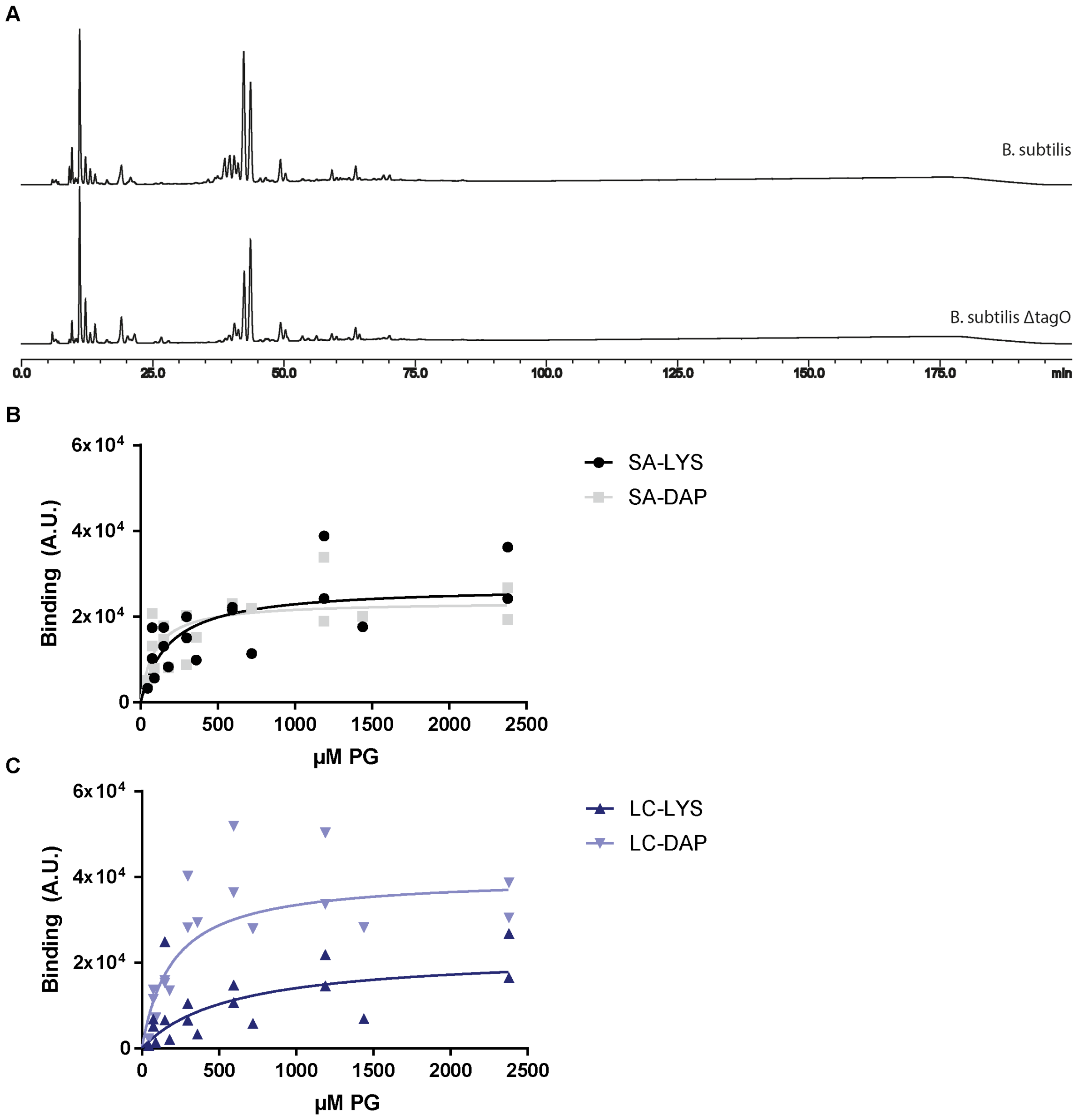

Figure S5. HPLC profiles of *B. subtilisΔTagO* and its parental strain and saturation binding of PG to PGRPs. Related to Figure 4. (A) ***HPLC Profiles***: muropeptide composition of the two *B. subtilis* strains used were identical as seen by HPLC analysis of mutanolysin-digested PG. Therefore, removal of WTA did not affect PG composition.

(B and C) ***Saturation binding experiments:*** Curves of best fit in PG saturation binding experiments where the concentration of each PGRP was kept constant while the amount of quantified PG added to the reaction was increased until no more PGRP precipitated with the addition of the PG ligand. **(B)** There was no significant difference (non-linear regression analysis) between the binding of PGRP-SA to Lys-type and DAP-type PG. **(C)** In contrast, there was significant preference of PGRP-LC for DAP-type PG and comparable to PGRP-SA (but lower) binding to Lys-type PG.

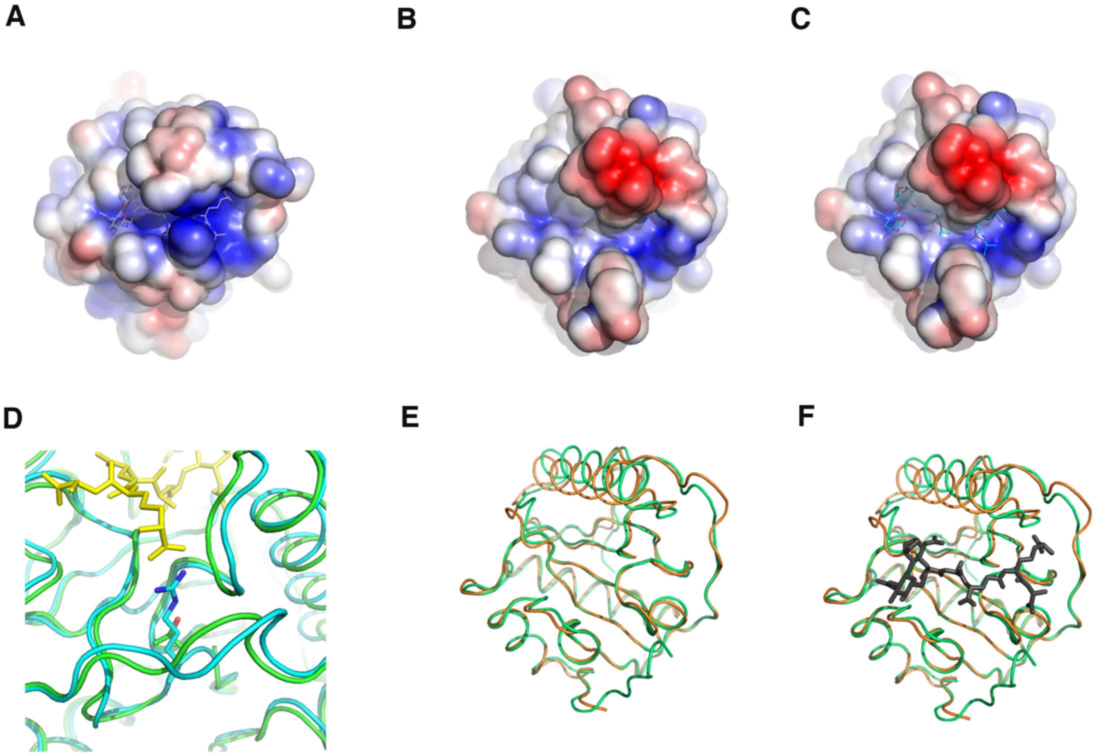

Figure S6. Structural meta-analysis of PGRP-LC and PGRP-SA binding to PG. Related to Figure 4. APBS/Pymol Electrostatic surface representations of: **(A)** PGRP-LC (with TCT in stick representation), **(B)** PGRP-SA, and **(C)** PGRP-SA with the TCT in position as placed by the superposition of the protein backbone of PGRP-LC against PGRP-SA. **(D)** A close-up of TCT bound to PGRP-LC (cyan) with the structure of PGRP-SA (green) superposed; the key Arg413 that docks DAP in PGRP-LC is shown (blue) along with the Threonine (red) that takes its place in PGRP-SA. **(E)** Calpha-defined superposition of PGRP-LC and PGRP-SA and **(F)** the same with TCT bound.

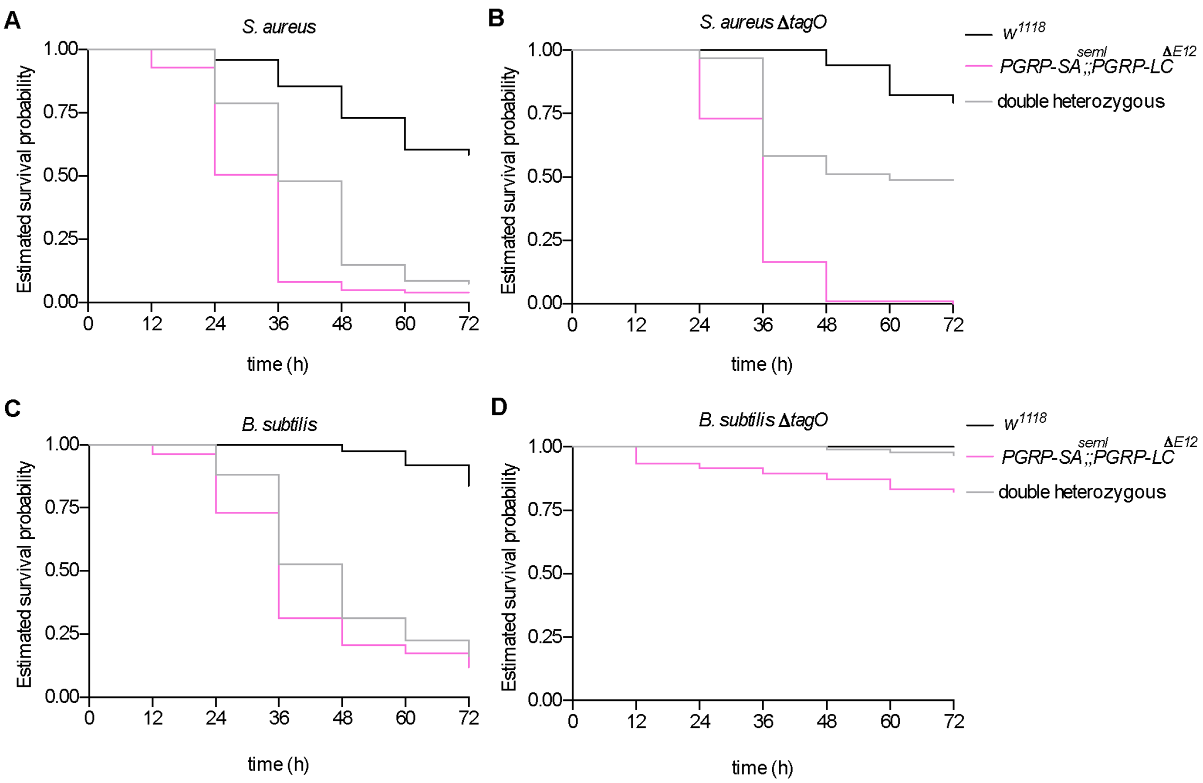

Figure S7. Survival probabilities of controls vs. double *PGRP-SA^{seml};;PGRP-LC$^{\Delta E12}$* homozygous mutants. Related to Figure 5. In all cases namely, after infection with **(A)** *S. aureus* **(B)** *S. aureusΔTagO* **(C)** *B. subtilis* **(D)** *B. subtilisΔTagO, w1118* flies were the most resistant. The double homozygous mutant was the most susceptible while the double heterozygote mutant was significantly different from the double homozygote in all cases and significantly different from wild type in cases A, B and C (stats in Table S3).

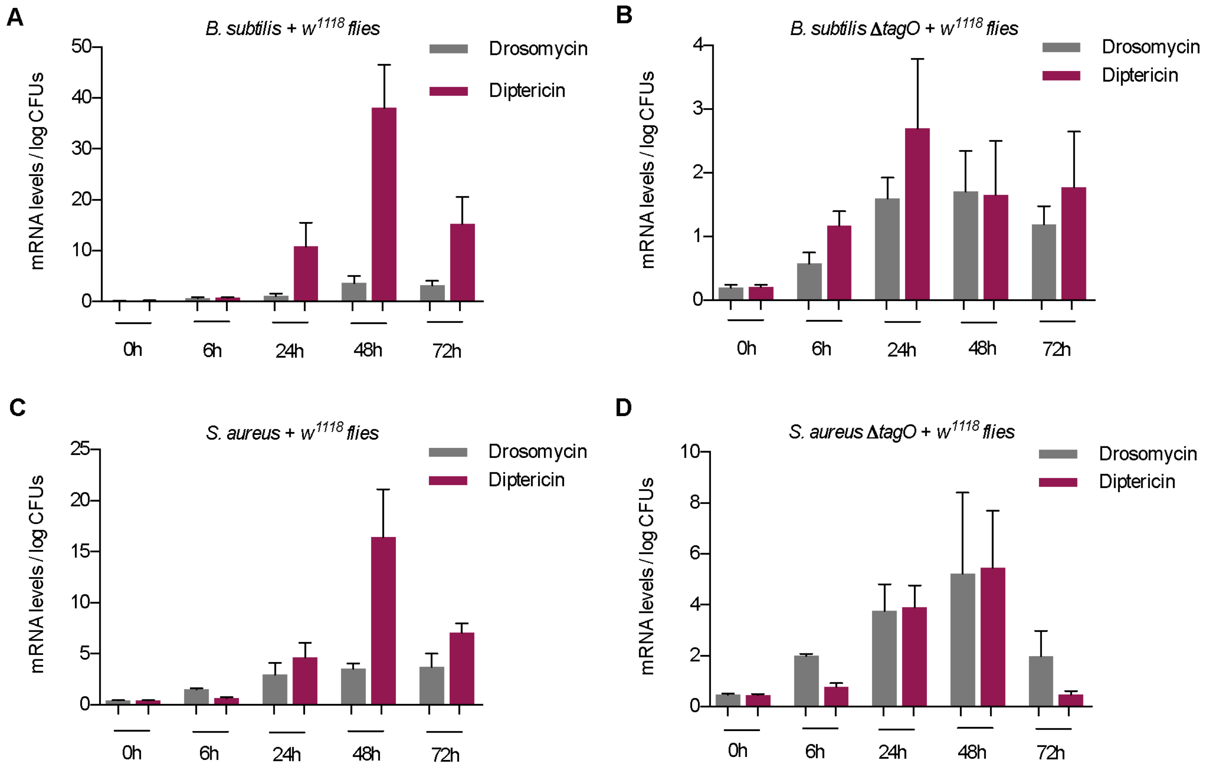

Figure S8. Pattern of AMP induction in *w[1118]* flies normalized to CFUs. Related to Figure 7. (A) Infection by *B. subtilis* induced differential AMP activation with significant differences between *dipt* and *drs*. **(B)** These differences disappeared after infection with *B. subtilisΔTagO*. **(C)** Similarly, infection with *S. aureus* induced differential AMP expression that was abolished **(D)** when flies were infected with *S. aureusΔTagO.*

Statistics (SF3 and SF4)	
**Ordinary one-way ANOVA**	
Number of families	1
Number of comparisons per family	28
Alpha	0.05

Uncorrected Fisher's LSD	Mean Diff.	95% CI of diff.	Significant?	Summary	Individual P Value
PGRP-LC + *B. subtilis* vs. PGRP-LC + *B. subtilis Δtago*	-12690	-12873 to -12507	Yes	****	< 0.0001
PGRP-LC + *B. subtilis* vs. PGRP-LC + *S. aureus*	59.91	-92.49 to 212.3	No	ns	0.4408
PGRP-LC + *B. subtilis* vs. PGRP-LC + *S. aureus Δtago*	-16.07	-171.0 to 138.9	No	ns	0.8389
PGRP-LC + *B. subtilis* vs. PGRP-SA + *B. subtilis*	40.07	-181.2 to 261.4	No	ns	0.7225
PGRP-LC + *B. subtilis* vs. PGRP-SA + *B. subtilis Δtago*	-2058	-2235 to -1881	Yes	****	< 0.0001
PGRP-LC + *B. subtilis* vs. PGRP-SA + *S. aureus*	51.89	-99.80 to 203.6	No	ns	0.5024
PGRP-LC + *B. subtilis* vs. PGRP-SA + *S. aureus Δtago*	-480.4	-630.7 to -330.1	Yes	****	< 0.0001
PGRP-LC + *B. subtilis Δtago* vs. PGRP-LC + *S. aureus*	12750	12614 to 12887	Yes	****	< 0.0001
PGRP-LC + *B. subtilis Δtago* vs. PGRP-LC + *S. aureus Δtago*	12674	12535 to 12814	Yes	****	< 0.0001
PGRP-LC + *B. subtilis Δtago* vs. PGRP-SA + *B. subtilis*	12730	12520 to 12941	Yes	****	< 0.0001
PGRP-LC + *B. subtilis Δtago* vs. PGRP-SA + *B. subtilis Δtago*	10633	10469 to 10796	Yes	****	< 0.0001
PGRP-LC + *B. subtilis Δtago* vs. PGRP-SA + *S. aureus*	12742	12607 to 12878	Yes	****	< 0.0001
PGRP-LC + *B. subtilis Δtago* vs. PGRP-SA + *S. aureus Δtago*	12210	12076 to 12344	Yes	****	< 0.0001
PGRP-LC + *S. aureus* vs. PGRP-LC + *S. aureus Δtago*	-75.98	-171.6 to 19.62	No	ns	0.1192
PGRP-LC + *S. aureus* vs. PGRP-SA + *B. subtilis*	-19.84	-204.5 to 164.9	No	ns	0.8332
PGRP-LC + *S. aureus* vs. PGRP-SA + *B. subtilis Δtago*	-2118	-2246 to -1989	Yes	****	< 0.0001
PGRP-LC + *S. aureus* vs. PGRP-SA + *S. aureus*	-8.02	-98.25 to 82.22	No	ns	0.8617
PGRP-LC + *S. aureus* vs. PGRP-SA + *S. aureus Δtago*	-540.3	-628.2 to -452.5	Yes	****	< 0.0001
PGRP-LC + *S. aureus Δtago* vs. PGRP-SA + *B. subtilis*	56.14	-130.6 to 242.9	No	ns	0.5556
PGRP-LC + *S. aureus Δtago* vs. PGRP-SA + *B. subtilis Δtago*	-2042	-2173 to -1910	Yes	****	< 0.0001
PGRP-LC + *S. aureus Δtago* vs. PGRP-SA + *S. aureus*	67.96	-26.49 to 162.4	No	ns	0.1584
PGRP-LC + *S. aureus Δtago* vs. PGRP-SA + *S. aureus Δtago*	-464.4	-556.6 to -372.2	Yes	****	< 0.0001
PGRP-SA + *B. subtilis* vs. PGRP-SA + *B. subtilis Δtago*	-2098	-2303 to -1892	Yes	****	< 0.0001
PGRP-SA + *B. subtilis* vs. PGRP-SA + *S. aureus*	11.82	-172.3 to 195.9	No	ns	0.8998
PGRP-SA + *B. subtilis* vs. PGRP-SA + *S. aureus Δtago*	-520.5	-703.5 to -337.5	Yes	****	< 0.0001
PGRP-SA + *B. subtilis Δtago* vs. PGRP-SA + *S. aureus*	2110	1982 to 2237	Yes	****	< 0.0001
PGRP-SA + *B. subtilis Δtago* vs. PGRP-SA + *S. aureus Δtago*	1577	1451 to 1703	Yes	****	< 0.0001
PGRP-SA + *S. aureus* vs. PGRP-SA + *S. aureus Δtago*	-532.3	-619.0 to -445.7	Yes	****	< 0.0001

Table S1. Related to Figures 1, 2, S2, S3.

PGRPs							
mCherry_PGRP-SA				mCherry_PGRP-LC			
Lys Type PG		DAP Type PG		Lys Type PG		DAP Type PG	
Kd	Bmax	Kd	Bmax	Kd	Bmax	Kd	Bmax
182.4	27054	88.94	23486	575.9	22152	198.3	40085

Table S2. Binding affinity (Kd) and Maximum binding (Bmax) of mCherry_PGRP-SA and mCherry_PGRP-LC to Lys type and DAP type peptidoglycan. Related to Figure 4 and Figures S5B and S5C. Calculations were made with GraphPad using one site specific binding regression curve. It is based on the Michaelis-Menten enzymology equation {S + E <-> [SE] -> E + P} where S = substrate, E = enzyme, P = product. Based on published structures (see Fig. S6 above), we assume that PGRP-SA and PGRP-LC have only one binding site for PG, which means that 1 PGRP molecule binds to PG only once.

Statistics for Figure 5A								
Log-rank test	*w*^{1118} vs *PGRP-LC*$^{\Delta E12}$	*w*^{1118} vs *PGRP-SA*	*w*^{1118} vs *PGRP-SA;;PGRP-LC*$^{\Delta E12}$	*w*^{1118} vs double hetero	*PGRP-LC*$^{\Delta E12}$ vs *PGRP-SA*	*PGRP-SA* vs *PGRP-SA;;PGRP-LC*$^{\Delta E12}$	*PGRP-LC*$^{\Delta E12}$ vs *PGRP-SA;;PGRP-LC*$^{\Delta E12}$	*PGRP-SA;;PGRP-LC*$^{\Delta E12}$ *vs double hetero*
Chi square	3.908	85.99	79.19	45.5	64.36	7.402	69.55	30.87
df	1	1	1	1	1	1	1	1
P value	0.0481	< 0.0001	< 0.0001	< 0.0001	< 0.0001	0.0065	< 0.0001	< 0.0001
P value summary	*	****	****	****	****	**	****	****
Are the survival curves sig different?	Yes	Yes	Yes	Yes	Yes	Yes	Yes	Yes

Statistics for Figure 5B								
Log-rank test	*w*^{1118} vs *PGRP-LC*$^{\Delta E12}$	*w*^{1118} vs *PGRP-SA*	*w*^{1118} vs *PGRP-SA;;PGRP-LC*$^{\Delta E12}$	*w*^{1118} vs double hetero	*PGRP-LC*$^{\Delta E12}$ vs *PGRP-SA*	*PGRP-SA* vs *PGRP-SA;;PGRP-LC*$^{\Delta E12}$	*PGRP-LC*$^{\Delta E12}$ vs *PGRP-SA;;PGRP-LC*$^{\Delta E12}$	*PGRP-SA;;PGRP-LC*$^{\Delta E12}$ *vs double hetero*
Chi square	4.004	84.25	97.69	10.45	82.49	15.82	108	74.96
df	1	1	1	1	1	1	1	1
P value	0.0454	< 0.0001	< 0.0001	0.0012	< 0.0001	< 0.0001	< 0.0001	< 0.0001
P value summary	*	****	****	**	****	****	****	****
Are the survival curves sig different?	Yes	Yes	Yes	Yes	Yes	Yes	Yes	Yes

Statistics for Figure 5C								
Log-rank test	*w*^{1118} vs *PGRP-LC*$^{\Delta E12}$	*w*^{1118} vs *PGRP-SA*	*w*^{1118} vs *PGRP-SA;;PGRP-LC*$^{\Delta E12}$	*w*^{1118} vs double hetero	*PGRP-LC*$^{\Delta E12}$ vs *PGRP-SA*	*PGRP-SA* vs *PGRP-SA;;PGRP-LC*$^{\Delta E12}$	*PGRP-LC*$^{\Delta E12}$ vs *PGRP-SA;;PGRP-LC*$^{\Delta E12}$	*PGRP-SA;;PGRP-LC*$^{\Delta E12}$ *vs double hetero*
Chi square	24.48	56.36	66.87	50.22	11.05	8.178	25.7	25.7
df	1	1	1	1	1	1	1	1
P value	< 0.0001	< 0.0001	< 0.0001	< 0.0001	0.0009	0.0042	< 0.0001	< 0.0001
P value summary	****	****	****	****	***	**	****	****
Are the survival curves sig different?	Yes	Yes	Yes	Yes	Yes	Yes	Yes	Yes

Statistics for Figure 5D								
Log-rank test	*w*^{1118} vs *PGRP-LC*$^{\Delta E12}$	*w*^{1118} vs *PGRP-SA*	*w*^{1118} vs *PGRP-SA;;PGRP-LC*$^{\Delta E12}$	*w*^{1118} vs double hetero	*PGRP-LC*$^{\Delta E12}$ vs *PGRP-SA*	*PGRP-SA* vs *PGRP-SA;;PGRP-LC*$^{\Delta E12}$	*PGRP-LC*$^{\Delta E12}$ vs *PGRP-SA;;PGRP-LC*$^{\Delta E12}$	*PGRP-SA;;PGRP-LC*$^{\Delta E12}$ *vs double hetero*
Chi square	0.7735	1.173	6.915	1.18	0.255	20.45	13.11	11.04
df	1	1	1	1	1	1	1	1
P value	0.3791	0.2788	0.0085	0.2774	0.6136	< 0.0001	0.0003	0.0009
P value summary	ns	ns	**	ns	ns	****	***	***
Are the survival curves sig different?	No	No	Yes	No	No	Yes	Yes	Yes

Table S3. Statistics of survival probabilities (log-rank test) related to Fig 5.

Statistics for Figure 5A								
Gehan-Breslow-Wilcoxon test	*w*^{1118} vs *PGRP-LC*$^{\Delta E12}$	*w*^{1118} vs *PGRP-SA*	*w*^{1118} vs *PGRP-SA;;PGRP-LC*$^{\Delta E12}$	*w*^{1118} vs double hetero	*PGRP-LC*$^{\Delta E12}$ vs *PGRP-SA*	*PGRP-SA* vs *PGRP-SA;;PGRP-LC*$^{\Delta E12}$	*PGRP-LC*$^{\Delta E12}$ vs *PGRP-SA;;PGRP-LC*$^{\Delta E12}$	*PGRP-SA;;PGRP-LC*$^{\Delta E12}$ *vs double hetero*
Chi square	4.424	69.37	72.42	39.15	40.52	13.66	57.77	37.81
df	1	1	1	1	1	1	1	1
P value	0.0427	< 0.0001	< 0.0001	< 0.0001	< 0.0001	0.0002	< 0.0001	< 0.0001
P value summary	*	****	****	****	****	***	****	****
Are the survival curves sig different?	Yes	Yes	Yes	Yes	Yes	Yes	Yes	Yes

Statistics for Figure 5B								
Gehan-Breslow-Wilcoxon test	*w*^{1118} vs *PGRP-LC*$^{\Delta E12}$	*w*^{1118} vs *PGRP-SA*	*w*^{1118} vs *PGRP-SA;;PGRP-LC*$^{\Delta E12}$	*w*^{1118} vs double hetero	*PGRP-LC*$^{\Delta E12}$ vs *PGRP-SA*	*PGRP-SA* vs *PGRP-SA;;PGRP-LC*$^{\Delta E12}$	*PGRP-LC*$^{\Delta E12}$ vs *PGRP-SA;;PGRP-LC*$^{\Delta E12}$	*PGRP-SA;;PGRP-LC*$^{\Delta E12}$ *vs double hetero*
Chi square	5.442	69.12	75.96	14.36	57.46	15.91	85.11	58.24
df	1	1	1	1	1	1	1	1
P value	0.0197	< 0.0001	< 0.0001	0.0002	< 0.0001	< 0.0001	< 0.0001	< 0.0001
P value summary	*	****	****	***	****	****	****	****
Are the survival curves sig different?	Yes	Yes	Yes	Yes	Yes	Yes	Yes	Yes

Statistics for Figure 5C								
Gehan-Breslow-Wilcoxon test	*w*^{1118} vs *PGRP-LC*$^{\Delta E12}$	*w*^{1118} vs *PGRP-SA*	*w*^{1118} vs *PGRP-SA;;PGRP-LC*$^{\Delta E12}$	*w*^{1118} vs double hetero	*PGRP-LC*$^{\Delta E12}$ vs *PGRP-SA*	*PGRP-SA* vs *PGRP-SA;;PGRP-LC*$^{\Delta E12}$	*PGRP-LC*$^{\Delta E12}$ vs *PGRP-SA;;PGRP-LC*$^{\Delta E12}$	*PGRP-SA;;PGRP-LC*$^{\Delta E12}$ *vs double hetero*
Chi square	26.4	54.24	61	47.78	8.374	14.1	25.34	11.84
df	1	1	1	1	1	1	1	1
P value	< 0.0001	< 0.0001	< 0.0001	< 0.0001	0.0038	0.0002	< 0.0001	0.0006
P value summary	****	****	****	****	**	***	****	***
Are the survival curves sig different?	Yes	Yes	Yes	Yes	Yes	Yes	Yes	Yes

Statistics for Figure 5D								
Gehan-Breslow-Wilcoxon test	*w*^{1118} vs *PGRP-LC*$^{\Delta E12}$	*w*^{1118} vs *PGRP-SA*	*w*^{1118} vs *PGRP-SA;;PGRP-LC*$^{\Delta E12}$	*w*^{1118} vs double hetero	*PGRP-LC*$^{\Delta E12}$ vs *PGRP-SA*	*PGRP-SA* vs *PGRP-SA;;PGRP-LC*$^{\Delta E12}$	*PGRP-LC*$^{\Delta E12}$ vs *PGRP-SA;;PGRP-LC*$^{\Delta E12}$	*PGRP-SA;;PGRP-LC*$^{\Delta E12}$ *vs double hetero*
Chi square	0.7324	0.994	6.551	1.01	0.325	17.672	8.75	9.52
df	1	1	1	1	1	1	1	1
P value	0.3851	0.3121	0.0094	0.3192	0.7127	< 0.0001	0.0008	0.0023
P value summary	ns	ns	**	ns	ns	***	**	**
Are the survival curves sig different?	No	No	Yes	No	No	Yes	Yes	Yes

Table S4. Statistics of survival probabilities (Gehan-Breslow-Wilcoxon test) related to Fig 5.
