# Supplementary material for: Accessibility to Peptidoglycan Is Important for the Recognition of Gram-Positive Bacteria in Drosophila
Source: Cell Rep. 2019 May 21;27(8):2480–2492.e6. doi: 10.1016/j.celrep.2019.04.103 (PMC6533200; doi:10.1016/j.celrep.2019.04.103)
Supplement: Document S2. Article plus Supplemental Information [file mmc4.pdf]

Cell Reports

Article

Accessibility to Peptidoglycan Is Important for the Recognition of Gram-Positive Bacteria in *Drosophila*

Graphical Abstract

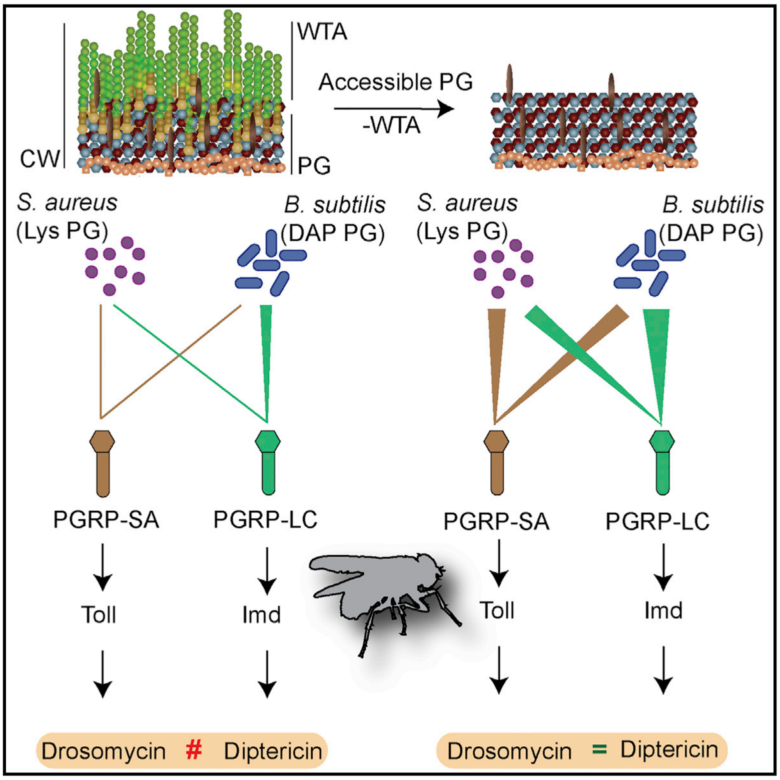

Highlights

- PGRP-SA equally recognizes Lys- and DAP-type peptidoglycans (PGs)
- PGRP-LC binds to *S. aureus* and triggers a robust response
- When WTAs are removed, immunity depends on both PGRP-SA and -LC
- Accessibility to PG plays a central role in bacterial sensing

Authors

Filipa Vaz, Ilias Kounatidis, Gonçalo Covas, ..., Ilan Davis, Sergio Raposo Filipe, Petros Ligoxygakis

Correspondence

sfilipe@fct.unl.pt (S.R.F.),
petros.ligoxygakis@bioch.ox.ac.uk (P.L.)

In Brief

It is widely believed that preference in recognition of structural features of bacterial peptidoglycan (PG) drives specificity in the *Drosophila* antibacterial response. Vaz et al. challenge this dogma by showing that accessibility to PG plays a central role in bacterial sensing and that structural discrimination is much less stringent than previously thought.

Vaz et al., 2019, Cell Reports *27*, 2480–2492
May 21, 2019 © 2019 The Authors.
https://doi.org/10.1016/j.celrep.2019.04.103

CellPress

OPEN ACCESS
CellPress

Cell Reports
Article

Accessibility to Peptidoglycan Is Important for the Recognition of Gram-Positive Bacteria in *Drosophila*

Filipa Vaz,[1,2,5] Ilias Kounatidis,[1,3,5] Gonçalo Covas,[2,4,5] Richard M. Parton,[1] Maria Harkiolaki,[3] Ilan Davis,[1] Sergio Raposo Filipe,[2,4,6,*] and Petros Ligoxygakis[1,6,7,*]
[1]Department of Biochemistry, University of Oxford, South Parks Rd., OX1 3QU Oxford, UK
[2]Instituto de Tecnologia Química e Biológica António Xavier, Universidade Nova de Lisboa, Avenida da República, 2780-157 Oeiras, Portugal
[3]Diamond Light Source, Ltd., Harwell Science and Innovation Campus, OX11 0DE Didcot, UK
[4]UCIBIO-REQUIMTE, Departamento de Ciências da Vida, Faculdade de Ciências e Tecnologia, Universidade Nova de Lisboa, 2829-516 Caparica, Portugal
[5]These authors contributed equally
[6]Senior author
[7]Lead Contact
*Correspondence: sfilipe@fct.unl.pt (S.R.F.), petros.ligoxygakis@bioch.ox.ac.uk (P.L.)
https://doi.org/10.1016/j.celrep.2019.04.103

SUMMARY

In *Drosophila*, it is thought that peptidoglycan recognition proteins (PGRPs) SA and LC structurally discriminate between bacterial peptidoglycans with lysine (Lys) or diaminopimelic (DAP) acid, respectively, thus inducing differential antimicrobial transcription response. Here, we find that accessibility to PG at the cell wall plays a central role in immunity to infection. When wall teichoic acids (WTAs) are genetically removed from *S. aureus* (Lys type) and *Bacillus subtilis* (DAP type), thus increasing accessibility, the binding of both PGRPs to either bacterium is increased. PGRP-SA and -LC double mutant flies are more susceptible to infection with both WTA-less bacteria. In addition, WTA-less bacteria grow better in PGRP-SA/-LC double mutant flies. Finally, infection with WTA-less bacteria abolishes any differential activation of downstream antimicrobial transcription. Our results indicate that accessibility to cell wall PG is a major factor in PGRP-mediated immunity and may be the cause for discrimination between classes of pathogens.

INTRODUCTION

As the first line of host defense, innate immunity is activated by a non-clonal system of receptors (Medzhitov and Janeway, 1997). These receptors sense microbial molecules and subsequently trigger specific signaling pathways that regulate target genes. The virtue of the system lies in the distinctive connection between individual microbes and the activation of certain sets of target host immune genes. Thus, each pathogen is associated with a specific transcriptional outcome in host defense (Medzhitov and Janeway, 1997).

In *Drosophila*, there is differential transcriptional activation by nuclear factor κB (NF-κB) pathways, depending on the bacterial type (Lemaitre et al., 1997; De Gregorio et al., 2002). This has been attributed to the ability of the upstream host receptors, peptidoglycan recognition proteins (PGRPs), to discriminate between different structures of bacterial peptidoglycan (PG) (for review, see Royet and Dziarski, 2007). PGRPs are highly conserved from insects to mammals and show distinct specificities in PG recognition. These specificities are thought to be the main determinant of the downstream response outcome.

Polymeric PG is composed of repeating units of muropeptide, comprising disaccharide *N*-acetyl glucosaminyl (GlcNAc)-*N*-acetylmuramic acid (MurNAC) linked to a stem peptide of D- or L- (or *meso*-) amino acids, in which the third amino acid is lysine (Lys) in Gram-positive bacteria and diaminopimelic acid (DAP) in Gram-negative bacteria and in certain Gram-positive bacilli. Circulating PGRP-SA is thought to interact preferentially with Lys-type PG and thus activate the Toll pathway (Leulier et al., 2003). In contrast, the cell membrane-associated PGRP-LC and intracellular PGRP-LE have been identified as the immunodeficiency (IMD) cascade receptors sensing DAP-type PG (Leulier et al., 2003; Kaneko et al., 2004; Takehana et al., 2002). Activation of these pathways culminates in the NF-κB-dependent differential induction of antimicrobial peptide (AMP) genes (reviewed in Kounatidis and Ligoxygakis, 2012). This differential induction is thought to be the consequence of upstream structural discrimination in PG recognition.

This view of Lys versus DAP PG discrimination has been based on the following evidence.

1. Interaction assays of recombinant PGRP-SA with purified PG have shown binding to both Lys- and DAP-type Gram-negative PG, but not to amidated DAP-type found in Gram-positive bacilli such as *Bacillus subtilis* and *B. thuringiensis* (Chang et al., 2004; Mellroth et al., 2005; Leone et al., 2008). In all of these experiments, however, PG was not quantified in terms of the number of sugar molecules added to the reaction, but only in terms of equal

 © 2019 The Authors.
This is an open access article under the CC BY license (http://creativecommons.org/licenses/by/4.0/).

weight, precluding any insight into potentially real differences of PGRP-SA binding to different types of PG. Moreover, these assays were performed either using a 20-mM Tris-HCl, pH 7.8, 300-mM NaCl buffer (Chang et al., 2004; Leone et al., 2008) or a 20-mM HEPES, pH 7.2, 150-mM NaCl buffer (Mellroth et al., 2005). Therefore, the binding conditions were not similar to what may happen in the hemolymph (insect equivalent of mammalian blood), in which all of these interactions take place. PG binding assays of PGRP-LE (albeit with the same experimental drawbacks outlined above) also suggested a clear binding preference for DAP-type PG (Takehana et al., 2002). In a notable exception, however, Mellroth et al. (2005) have observed the binding of PGRP-LCx (the PGRP-LC isoform with PG binding ability) to polymeric Lys-type PG *in vitro.*

2. Structural studies have sought to corroborate the suggested Lys/DAP structural discrimination by comparing and contrasting the unligated structure of PGRP-SA (Chang et al., 2004; Reiser et al., 2004), with the structure of PGRP-LC co-crystallized with a naturally occurring DAP-type PG fragment from *Bordetella pertusis*, known as tracheal cytotoxin (TCT; Chang et al., 2006). TCT, which is essentially monomeric PG (GlcNAc-1,6-anhydro-MurNAc-L-Ala-γ-D-Glu-*meso*-DAP-D-Ala), strongly activates IMD through PGRP-LC (Kaneko et al., 2004). In the PGRP-LC/TCT structure, Arg^{413}, Asp^{395}, and Trp^{394} of PGRP-LCx form a binding pocket for the DAP group. Since this pocket is surrounded by three solvent-exposed basic residues (Arg^{349}, Lys^{423}, and Arg^{427}), the authors of the report argue that the presence of an Lys instead of a DAP would destabilize the docking interaction. Moreover, the absence of positively charged residues in the same region of PGRP-SA would be consistent with preferred binding to Lys-type PG (Chang et al., 2006). However, evidence from a crystal structure will always provide a "freeze-frame," and therefore these assertions may prove problematic when considering the whole "movie." For example, one could argue that the positive charge potential of the three solvent-exposed residues mentioned above is sufficiently flexible and distant (>10 Å) from any ligand that it would not interfere with binding to Lys. Moreover, their surface positive charge potential may be fulfilled with water (see Results for a more comprehensive appraisal of alternative options).

It is also not clear how widespread the recognition of monomeric PG fragments is, as opposed to polymeric ones. If the latter is also a binding possibility, then the amino group linked to the epsilon carbon of the Lys residue in polymeric Lys-type PG will not retain a basic charge if there is a cross-bridge linking it to D-Ala and consequently connecting two different PG stem peptides. Thus, as has been shown by Mellroth et al. (2005), this will lead to PGRP-LCx binding to Lys-type PG. The hypothetical binding of PGRP-SA to monomeric PG is also problematic, as it has been shown that PGRP-SA from the large beetle *Tenebrio molitor* activates downstream signaling through clustering to polymeric Lys-type PG (Park et al., 2007). In addition, monomeric muropeptides fail to activate Toll (Filipe et al., 2005), while muramidase-treated PG is unable to induce either pathway (Leulier et al., 2003; Filipe et al., 2005). Therefore, comparing the PGRP-LC/TCT structure with a hypothetical entity of PGRP-SA/Lys-type monomer may not be physiologically relevant. Structural studies using analogs of monomeric PG (muramyl pentapeptides; see Guan et al., 2004; Swaminathan et al., 2006) and PGRP-LC may have limited *in vivo* relevance due to the differences between the synthetic muropeptides that were used and the native ones.

Moreover, in *Tenebrio molitor*, polymeric DAP-type PG from *Bacillus subtilis* and *Escherichia coli* was bound by PGRP-SA, leading to activation of the proteolytic cascade upstream of Toll as Lys-type PG did (Yu et al., 2010). This indicated that any structural discrimination was certainly not an insect-wide phenomenon.

3. *In vivo* studies have also indicated that the injection of Lys-type PG activates Toll at least 3× more than DAP-type PG (Leulier et al., 2003). Again, the lack of quantification by the number of sugars injected (and not by weight, which was typically 50 mg/mL) precludes a direct comparison.

Our previous work has established that accessibility to PG played an important role in PGRP-SA recognition of whole Gram-positive bacteria with Lys-type PG. Wall teichoic acids (WTAs) are anionic polymers of glycerol- or ribitol-phosphate that conceal the PG layer from direct interaction with the extracellular environment. Removal of WTAs from *Staphylococcus aureus* (*S. aureus*Δ*tagO*), increased PGRP-SA binding to whole bacteria by 1,000× and made the PGRP-SA-dependent clearance of *S. aureus* independent of Toll (Atilano et al., 2011).

In this study, we address the importance of accessibility to PG in PGRP-mediated recognition of whole bacteria in more general terms. In addition to *S. aureus* as the Lys-type representative, we used *B. subtilis* as the DAP-type model. We found that in a hemolymph-like buffer, both PGRPs were able to bind *in vitro* both bacteria with exposed PGs at their surface, as well as their purified and quantified PG. When WTAs were removed, PGRP-SA was able to bind significantly more both types of bacteria, while PGRP-LC was able to bind significantly more only to *B. subtilis* and at the margin of statistical significance to *S. aureus*. In addition, there was no measurable preference of PGRP-SA binding to any of the two PGs *in vitro*, while PGRP-LC showed a quantifiable 5× preference for DAP-type PG. Despite that, PGRP-LC co-localized with the cell surface of both *S. aureus* and *S. aureus*-Δ*TagO ex vivo* and activated a robust antimicrobial response that was statistically comparable to that mediated by PGRP-SA. Following infection with *S. aureus* or *B. subtilis*, differential AMP induction was observed. However, when WTAs were removed, antimicrobial responses per bacterial number became statistically indistinguishable across the course of infection, indicating that more accessibility to PG equalized downstream transcription. At the level of the whole organism, flies that were double mutant for PGRP-SA and PGRP-LC were more susceptible than single mutants to infection with WTA-less bacteria, while the growth of WTA-less bacteria showed a statistically significant increase in double mutant flies compared to the single fly mutants.

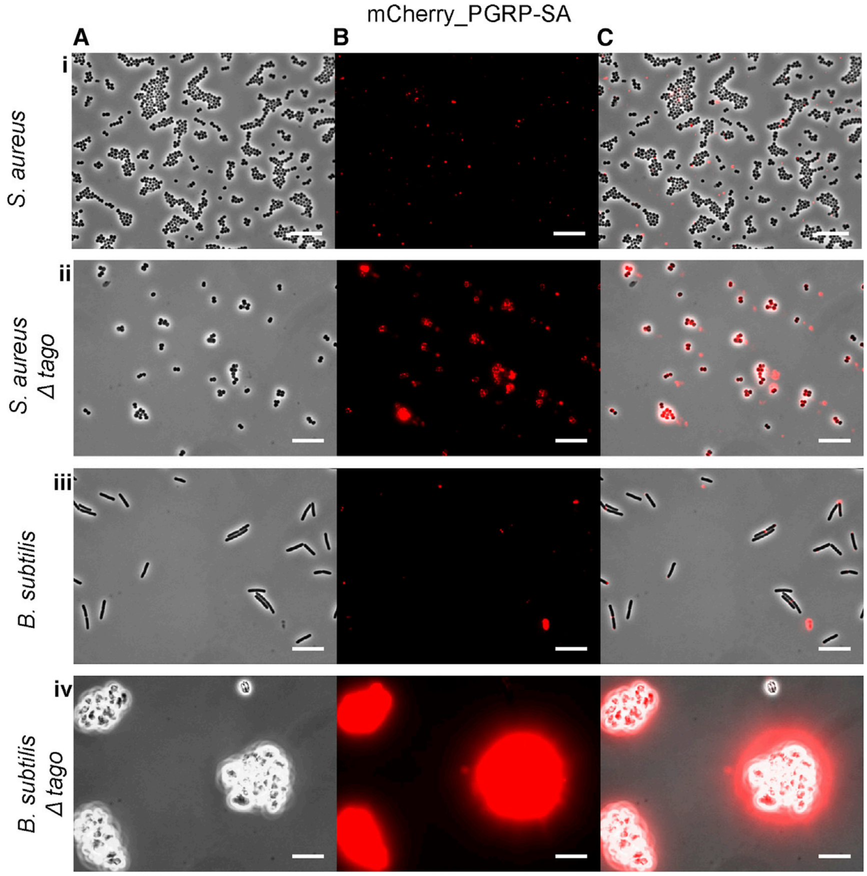

Figure 1. Binding of PGRP-SA to a Gram-Positive Bacterium and a Gram-positive Bacillus *In Vitro*

Binding of a fluorescently tagged PGRP-SA (mCherry_PGRP-SA) in (A) phase contrast channel, (B) mCherry channel, and (C) overlay. We tested for binding to *S. aureus* (Ai–Ci), *S. aureusTagO* (Aii–Cii), *B. subtilis* (Aiii–Ciii), and *B. subtilisTagO* (Aiv–Civ). Increased binding was consistently observed in the TagO mutants compared to their parental strains. See main text and Figure S1 for the quantification of median intensity binding.

We argue that accessibility to PG is one of the main determinants of PGRP specificity in *Drosophila*. Given the evolutionary conservation of innate immunity in most metazoans, our results have implications for animals when PGRPs are used as recognition receptors of whole bacteria.

RESULTS

We chose to use two bacteria (*S. aureus* and *B. subtilis*) that carry PGs that represent a wide range of bacteria and bacilli. In general, bacterial PG can be divided into two groups: group A (in which crosslinking between stem peptides occurs between positions 3 and 4), which is the most common among pathogenic bacteria (Schleifer and Kandler, 1972), and group B (in which crosslinking between stem peptides occurs between positions 2 and 4), found only among some coryneform bacteria, especially from plants (Schleifer and Kandler, 1972). *S. aureus* PG belongs to group A3 (i.e., crosslinking between stem peptides is made up of interpeptide bridges consisting of monocarboxylic L-amino acids or glycine or both). PG of the A3 group is very common among Gram-positive bacteria (Schleifer and Kandler, 1972). *B. subtilis* PG belongs to group A1 (i.e., different stem peptides are connected through direct crosslinking). PG of the A1 group is very common among Gram-negative bacteria.

Previous studies (e.g., Lemaitre et al., 1997; Leulier et al., 2003; Chang et al., 2004) have used the Gram-positive bacterium *Micrococcus luteus* as "the Gram-positive bacterium of choice" for infection studies, and subsequently many labs used *M. luteus* PG for interaction studies. However, *M. luteus* PG belongs to group A2 (in which crosslinking is made by one polymerized stem peptide), which is not frequently found among bacteria (Schleifer and Kandler, 1972). The PGs that belong to this group have two important particularities: >50% of the *N*-acetylmuramic residues are not substituted by a stem peptide; all glycine residues in the native cell wall are C-terminal and therefore are not involved in the crosslinking between different stem peptides (Schleifer and Kandler, 1972). These characteristics make *M. luteus* PG not a representative choice for a Gram-positive bacterium. Moreover, *M. luteus* genetics is not at all developed, while both *S. aureus* and *B. subtilis* have been used as genetic models and thus we know a great deal about their cell wall physiology. This is an important point since only the use of bacterial genetics will reveal if and how Gram-positive bacteria and bacilli that have their PG directly exposed to the host conceal it from PGRPs.

Removal of WTAs Enhances Binding of PGRP-SA and PGRP-LC to Whole Bacteria

We hypothesized that differential activation of immunity by different bacterial species may not be due to the structural discrimination of PG, but instead is due to differential PG accessibility. To test this hypothesis, we compared the *in vitro* binding of fluorescently tagged PGRP subtypes to whole bacteria in a buffer similar to hemolymph (Croghan and Lockwood 1960; Van Der Meer and Jaffe, 1983; henceforth called HemoBuffer; see Method Details for recipe). Binding to the parental reference strains (National Collection of Type Cultures [NCTC] 8325-4 for *S. aureus* and EB6 for *B. subtilis*) was used as a control. Our results are shown in Figure 1 for PGRP-SA and Figure 2 for PGRP-LC, while quantification of these bindings is shown in Figure S1. To compare binding of mCherry-PGRP-SA and mCherry-PGRP-LC to different bacterial strains, we used a 10-ms exposure time. Given the variability in signal intensity, for visualization purposes only, the contrast of images shown in Figures 1 and 2 was

OPEN
ACCESS
CellPress

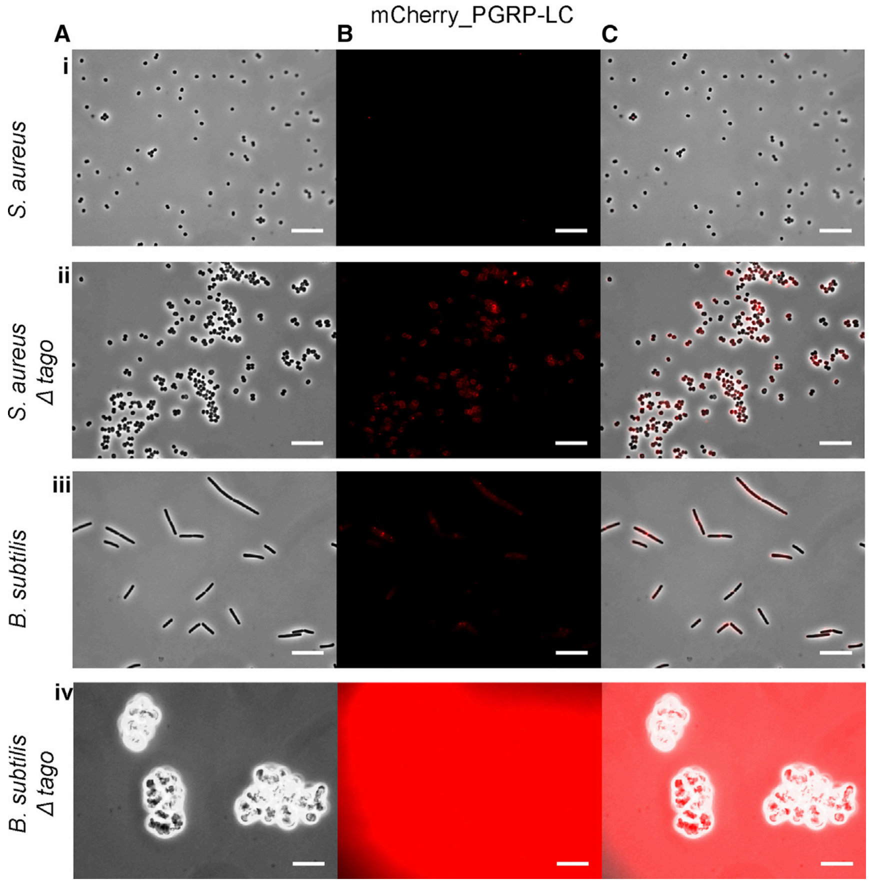

Figure 2. Binding of PGRP-LC to a Gram-Positive Bacterium and a Gram-Positive Bacillus *In Vitro*

Binding of a fluorescently tagged PGRP-LC (mCherry_PGRP-LC) in (A) phase contrast channel, (B) mCherry channel, and (C) overlay. We tested for binding to *S. aureus* (Ai–Ci), *S. aureusTagO* (Aii–Cii), *B. subtilis* (Aiii–Ciii), and *B. subtilisTagO* (Aiv–Civ). Increased binding was consistently observed in the TagO mutants compared to their parental strains. See main text and Figure S1 for the quantification of median intensity binding.

adjusted for easier visual comparison of images with high signal intensities, while the contrast in Figures S2A (PGRP-SA) and S2B (PGRP-LC) was adjusted for easier visual comparison of images with low signal intensities. Statistical tests for all of the possible comparisons are presented in Table S1.

PGRP-SA demonstrated no statistical difference between *S. aureus* NCTC and *B. subtilis* EB6 binding (Figures 1Ai–1Ci,1Aiii–1Ciii, S1A, and S1B; Table S1). The median intensity of the fluorescent signal of mCherry-PGRP-SA associated with bacteria was 16 and 23, respectively. Similarly, we found no significant difference between mCherry-PGRP-LC binding to *S. aureus* NCTC and *B. subtilis* EB6 (Figures 2Ai–2Ci, 2Aiii–2Ciii, S1C, and S1D; Table S1). The median intensity of the fluorescent signal of mCherry-PGRP-LC associated with bacteria was 13 and 64, respectively. Upon WTA removal, both PGRPs displayed increased recognition of both bacteria (see Figures 1Aii–1Cii and 1Aiv–1Civ for PGRP-SA and 2Aii–2Cii and 2Aiv–2Civ for PGRP-LC). Quantification showed that PGRP-SA binding was significantly increased in both *S. aureusΔTagO* (with a median fluorescent signal increase from 16 to 379) and *B. subtilisΔTagO* (with a median fluorescent signal increase from 23 to 1,593) (Figure S1B; Table S1).

However, it was crucial to verify that the increased PGRP binding to the *ΔTagO* mutants was not a result of the mCherry protein tag. To this end, we constructed and purified mutant versions of mCherry-PGRP-SA that carried either or both of the mutations C48 to Y and C54 to Y (Figure S3). A C54-to-Y change is the mutation present in *Drosophila PGRP-SA^{seml}* flies, which disrupts the highly conserved disulfide bridge formed between Cys48 and Cys54 (Michel et al., 2001). This bond tethers the H2 helix to the central β sheet through the L1 loop (Chang et al., 2004), and its disruption abrogates downstream signaling (Michel et al., 2001). In addition, to prevent the formation of alternative inter-disulfide bridges, we introduced the mutation C48 to Y. We reckoned that a C-to-Y rather than a C-to-A change would alleviate problems in expression of the C48A mutant observed previously (Chang et al., 2004). The expression of the recombinant m-Cherry and mCherry_PGRP-SA is shown in Figure S3A, while the expression of mCherry_PGRP-SA^{C48YC54Y} protein and the single mCherry_PGRP-SA^{C48Y} and mCherry_PGRP-SA^{C54Y} mutants is shown in Figure S3B. The single mutants showed a high number of multimer forms; however, the double mutant showed protein expression similar to that of the wild-type protein (Figure S3B). Despite its normal expression, mCherry_PGRP-SA^{C48YC54Y} was not able to bind the PG of either bacterium (Figure S3C). Consistent with this, the mutant mCherry_PGRP-SA^{C48YC54Y} was unable to bind to *S. aureus* NCTC and *S. aureusΔTagO* (Figure S4A) and to *B. subtilis* and *B. subtilisΔTagO* (Figure S4B). The results in Figures S3 and S4 clearly show that only the PGRP-SA part of the mCherry_PGRP-SA fusion was responsible for binding.

PGRP-LC binding to *B. subtilisΔTagO* was also significantly intensified compared to the parental strain with a median fluorescent signal that increased from 64 to 13,402 (Figures 2 and S1D; Table S1). Binding of PGRP-LC to *S. aureusΔTagO* was also increased from 13 to 88, but statistically it was just below a significant p value (Figure S1D; Table S1). Nevertheless, a closer look at the binding values of PGRP-LC to *S. aureusΔTagO* (Figure S1C) showed that most were above the *S. aureus* NCTC parental strain. Consistent with this, using a longer exposure time (500 ms) to visualize the fluorescent protein bound to the bacterial surface, PGRP-LC binding to *S. aureusΔTagO* was significantly increased (with a median fluorescent signal from 175.5 to 5,437; data not shown).

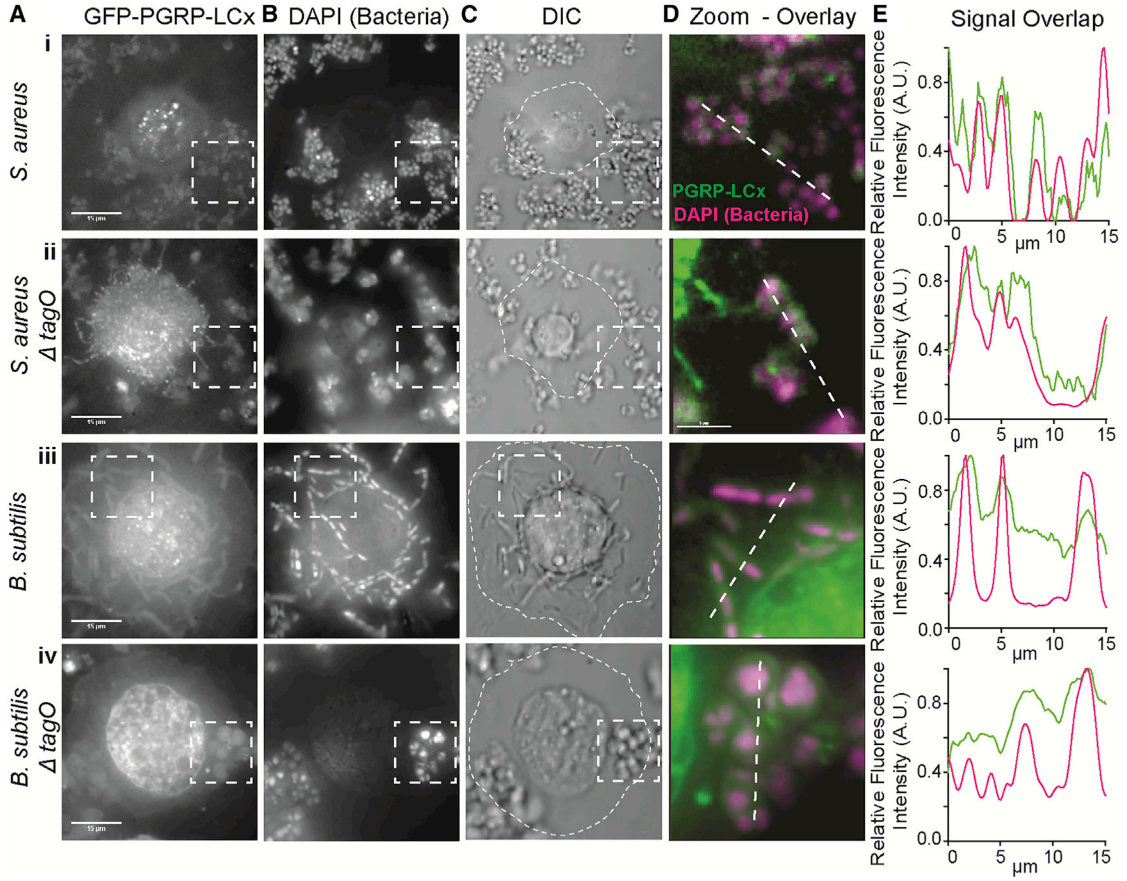

Figure 3. Binding of PGRP-LC to a Gram-Positive Bacterium and a Gram-Positive Bacillus *Ex Vivo*

The GFP-tagged transmembrane PGRP-LCx (GFP-PGRP-LCx, expressed in macrophages) was assessed by fluorescence imaging for binding to *S. aureus* (Ai–Ei), *S. aureusTagO* (Aii–Eii), *B. subtilis* (Aiii–Eiii), and *B. subtilisTagO* (Aiv–Eiv), respectively. (Ai–Aiv) GFP-PGRP-LCx-expressing hemocytes were mixed with bacteria. (Bi–Biv) Bacteria were pre-stained and identified by DAPI. (Ci)–(Civ) presents corresponding DIC images in which the extent of macrophages is indicated by the dashed outlines. (Di–Div) Dashed squared regions (82 μm) indicated in (A)–(C) are shown as a dual color overlay of GFP (PGRP-LC) and DAPI (bacteria) signals. (Ei–Eiv) Plots of fluorescence intensity along the dashed lines in (Di–Div) (15 μm transect) are shown highlighting the overlap of the extracellular signals between GFP and DAPI.

These results indicated that PGRP-SA showed no apparent discrimination between the two wild-type strains of bacteria. However, when WTAs were removed, PGRP-SA had a preference for *B. subtilisΔTagO*. PGRP-LC showed no discrimination between the wild-type parental strains. When WTAs were removed, *B. subtilisΔTagO* was the preferred strain of PGRP-LC, while binding to *S. aureus* increased but not as much. Nevertheless, the latter result was contrary to expectations, as PGRP-LC was not supposed to bind Gram-positive bacteria. Therefore, we wanted to explore the interaction of live *S. aureus* bacteria with primary macrophages expressing PGRP-LC on their membrane.

This was because our *in vitro* binding experiments used an mCherry-PGRP-LC construct that only included the PGRP ectodomain of the protein, so we wanted to ascertain that when expressed in *Drosophila* cells, transmembrane PGRP-LC was also able to bind to *S. aureus* and *B. subtilis* wild-type strains and their WTA-less mutants. To this end, we used a *UAS-PGRP-LCx-GFP* transgene (Schmidt et al., 2011) expressed in post-embryonic hemocytes through the *hemolectin-GAL4* driver. To visualize binding, we devised an *ex vivo* system of hemocyte imaging (see Method Details). Briefly, we bled *hml-GAL4; UAS-PGRP-LCx-GFP* larvae on a slide to isolate hemocytes, which were then kept in a humid chamber to preserve them for live imaging. Bacteria were stained with DAPI before being added to the hemocyte preparation. The results are shown in Figure 3.

Schmidt et al. (2011) have described the pathogen-induced detachment of a FLAG-tagged PGRP-LCx from the membrane of S2 cells upon infection with *E. coli* or *Salmonella typhimurium* (Gram negative), which did not happen following challenge with heat-killed Gram-negative bacteria or live *Staphylococcus carnosus* (Gram positive). We also observed that the fluorescent signal of the GFP linked to the extracellular domain of PGRP-LCx detached from the cell membrane and co-localized with bacteria away from the cell surface and in the extracellular space. However, this was observed both after challenge with live *S. aureus* (Figures 3Ai–3Di; Video S1 up to 0 min:10 s) and live *S. aureusΔTagO* (Figures 3Aii–3Dii; Video S1, 0 min:11

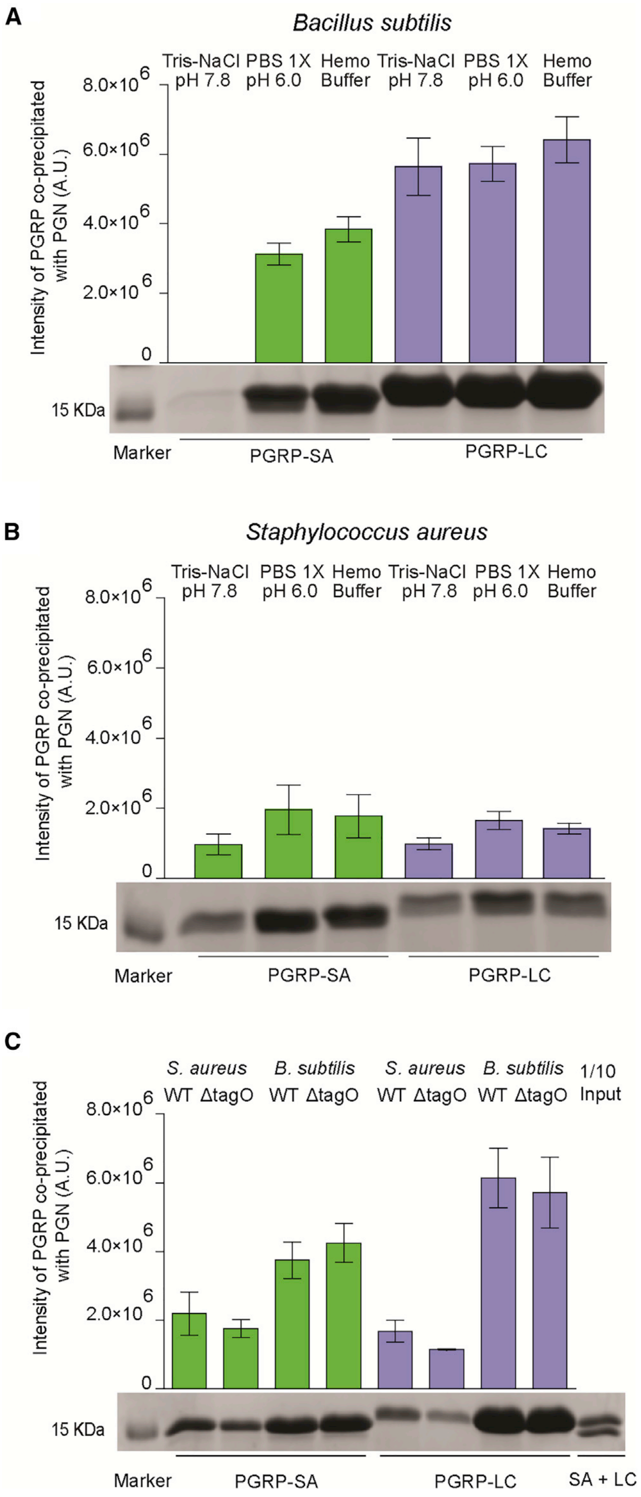

Figure 4. Binding of PGRP-SA and PGRP-LC to Purified and Quantified Peptidoglycan

(A) PGRP-SA was able to bind to DAP-type PG in PBS and HemoBuffer.

(B) Binding of PGRP-LC to the same type of PG was statistically higher, indicating a preference for DAP-type PG.

(C) In contrast, binding of the two PGRPs to *S. aureus* PG was statistically indistinguishable (when comparing binding assays in HemoBuffer). This was not the case in saturation experiments, however as seen in Figure S5. Binding of the two PGRPs to TagO versus parental strain PG was statistically indistinguishable, indicating that the PG itself did not change when WTAs were removed.

PG quantified as in Method Details. Bars in all of the graphs represent mean values, with error bars showing SDs. Statistical significance ($p < 0.005$) was determined through the Student's t test.

s–0 min:20 s). As expected, PGRP-LCx also interacted with *B. subtilis* and *B. subitilis⊿TagO* (Figures 3Aiii–3Diii and 3Aiv–3Div; data not shown).

Therefore, in keeping with our *in vitro* results as presented in Figures 2, S1, and S2, our imaging results with *hml-GAL4; UAS-PGRP-LCx-GFP* larval hemocytes indicated abundant interactions of cleaved PGRP-LCx at the level of the bacterial surface with both wild-type and WTA-less bacterial strains, irrespective of PG type. Moreover, this indicated that the use of only the PGRP-LC ectodomain was a meaningful way to test the binding of the receptor to PG, as (at the cellular level) it showed the same engagement with bacteria as the full-length protein.

PGRP-SA Binds Similarly to Purified and Quantified Polymeric Lys-Type and DAP-Type PG, whereas PGRP-LC Binds More to DAP-Type PG

To circumvent the caveats of previous studies (Chang et al., 2004; Mellroth et al., 2005; Leone et al., 2008), we conducted binding experiments using HemoBuffer and quantified pure polymeric PG from *S. aureus* and *B. subtilis*. Briefly (see the full protocol in Method Details), PG was hydrolyzed to glucosamine and muramic acid (controlling for the efficient removal of *N*-acetyl groups without affecting the sugar molecules and avoiding any WTA contamination) and chromatographically separated. The areas of chromatogram peaks were quantified in comparison to controls in which the concentration of glucosamine and muramic acid were known. With this method, we were able to determine exactly the amount of glucosamine and muramic acid used in our binding assays and therefore used precisely quantified PG. In turn, this helped in the use of PG suspensions with the same concentration of MurNAc in the binding reactions.

Our results indicated that contrary to previous observations (Chang et al., 2004; Leone et al., 2008), given the appropriate buffer, PGRP-SA was able to bind *B. subtilis* PG (Figure 4A). PGRP-LC was able to bind more to *S. aureus* PG in PBS or HemoBuffer than in Tris-NaCl (Figure 4B). In this context, binding of the two PGRPs to the Lys-type PG of *S. aureus* was statistically indistinguishable (Figure 4B). Binding of the two PGRPs to *B. subtilis* PG (DAP type) showed a statistically significant difference with PGRP-SA/PG co-immunoprecipitation intensity at 4×10^6 U, while PGRP-LC/PG co-immunoprecipitation intensity was at 6×10^6 U (Figure 4C). This indicated that PGRP-LC bound more to *B. subtilis* PG than to *S. aureus* PG, as previously proposed (Leulier et al., 2003; Kaneko et al., 2004; Filipe et al., 2005; Guan et al., 2004; Atilano et al., 2011). Nevertheless, we observed no statistical difference in the binding of each PGRP when comparing parental versus *⊿TagO* PG (Figure 4C). This indicated that the increase in binding of whole bacteria that we have identified with the removal of WTAs was not the result of a change in PG itself, but a consequence of increased

accessibility. In this context, we note that the high-performance liquid chromatography (HPLC) profiles of PGs from parental and *ΔTagO* strains were identical (Figure S5A for *B. subtilis*; see Atilano et al., 2010 for *S. aureus*).

To confirm the above results, we performed saturation-binding experiments, in which a fixed amount of each PGRP was individually mixed with increasing amounts of either PG to reach saturation binding (i.e., no increase in the PGRP protein precipitating with PG). Contrary to previous results (Chang et al., 2004), PGRP-SA in HemoBuffer did not display a significant preference between DAP-type (2.3×10^4 a.u. at saturation) and Lys-type PG (2.7×10^4 a.u. at saturation) (Figure S5B). This was confirmed by measuring the K_D and B_{max} of PGRP-SA binding of Lys-type and DAP-type PG (Table S2). The K_D for Lys-type PG binding of PGRP-SA was 182.4, whereas for DAP-type PG it was 88.94 (see Table S2; of note, the lower the K_D, the stronger the association).

In contrast, PGRP-LC showed a preference for DAP-type PG (Figure S5C; 4×10^4 a.u. at saturation) over Lys-type PG (Figure S5C; 2×10^4 a.u. at saturation) and bound significantly more DAP-type PG than PGRP-SA (compare curves for DAP-type PG in Figures S5B and S5C). However, the difference between binding of PGRP-SA and PGRP-LC to Lys-type PG was much less pronounced (2.7×10^4 a.u. for PGRP-SA and 2×10^4 a.u. for PGRP-LC; compare curves for Lys-PG in Figures S5B and S5C). This was corroborated by comparable B_{max} measurements (27,054 for PGRP-SA and 22,152 for PGRP-LC), indicating comparable numbers of PG-associated receptors in the PGRP-PG pellet at saturation. However, the K_Ds were different, indicating tighter binding of PGRP-SA to Lys-PG (Table S2).

Contrary to previous results, our data above show that PGRP-SA binds similarly to both types of PG. PGRP-LC prefers DAP-type PG but does bind to Lys-type PG, and at saturation, the numbers of LC receptors recovered in the reaction to Lys-type PG were comparable to PGRP-SA, which is considered the bona fide receptor of Gram-positive bacteria.

Structural Evidence Indicates Binding of PGRP-SA and PGRP-LC to Both Types of PG

Next, we wanted to investigate whether there is also structural evidence for the binding of PGRP-LC to Lys-type PG and whether the structure of PGRP-SA would allow binding to both Lys-type and DAP-type PG. Superposition of the two structures (Figures S6A–S6F) and examination of their electrostatic potential (Figures S6A–S6C) indicated several relevant features: the electrostatic potential at the surface of PGRP-LC (Figure S6A) calculated in the absence of PG would indicate a number of localized positively charged areas mainly associated with Lys423 and Arg349 (Chang et al., 2006). Arg427 is the third proposed surface residue to complete the Lys-type PG exclusion ring. However, its potential is tempered by a hydrogen bond formed with the backbone of residue 353. It is notable that both Lys423 and Arg349 are located on the surface of the molecule and do not form any H-bonds with neighboring residues. They should therefore be relatively free in solution to present as any of a number of rotameric variants. This in turn could lead to the relocation of any positive potential further away from the binding site or its neutralization via interactions with negatively charged residues on the molecular surface. This would suggest that Lys-PG binding is probable in solution at measurable rates. In turn, this is something corroborated by our binding assays. Nevertheless, based on this structure (Chang et al., 2006), the defining characteristic of the PGRP-LC binding groove is the conformation of Arg413, which securely anchors DAP-type PG and is likely to impose a preference for the latter via increased conformational stability (Figure S6D). This is also corroborated by our results from the binding of PGRP-LC to quantified PG (see Figures 4, S5A, and S5B; Table S2).

In the case of PGRP-SA, the structure (based on Reiser et al., 2004) is far more electronegative in the vicinity of the binding site (Figure S6B), and the positive sites are compromised by a lack of positively charged residues; here, R349 is replaced by a glycine (G27), Lys423 is a tyrosine (Y100), and Arg427 is now an alanine (A104). Moreover, the replacement of the anchoring arginine in PGRP-LC with threonine in PGRP-SA (Figure S6D) not only removes the two hydrogen bonds that would have stabilized DAP-PG but also leaves an accommodating void that can flexibly bind via a water molecule network to either type of PG (Figures S6E and S6F). In this case, there should be little difference in binding affinity. Although more structures need to be generated to confirm this, the prediction is that PGRP-SA will be more equal in the binding of the different types of PGs than PGRP-LC. Our PG binding experiments (Figure 4) and saturation experiments (Figures S5A and S5B; Table S2) showed that in the HemoBuffer, binding of PGRP-SA to DAP or Lys-type PG was statistically indistinguishable.

Flies Deficient for Both PGRP-SA and PGRP-LC Are More Susceptible to WTA-less Bacteria Than Flies Lacking Any One of These PGRPs

In broad terms, the working hypothesis in the field is that flies that are deficient for PGRP-SA are more susceptible to Gram-positive bacteria, while PGRP-LC mutants succumb to Gram-negative bacteria and Gram-positive bacilli (Kounatidis and Ligoxygakis, 2012). We wanted to test this hypothesis comparing WTA-less bacteria with their parental strains. Estimated survival curves were analyzed using both the log rank (Mantel-Cox, see Table S3) and the Gehan-Breslow-Wilcoxon test (see Table S4) to determine statistical significance between the survival probabilities. The main difference between the tests is that the Gehan-Breslow-Wilcoxon method gives more weight to deaths at early time points, which correlates with the fact that *S. aureus* kills flies fast (Atilano et al., 2011). In contrast, the Mantel-Cox test gives equal weight to all time points.

Infection with *S. aureus* NCTC did confirm that *PGRP-SA*^{seml} homozygous mutant flies were significantly more susceptible than *PGRP-LC*$^{\Delta E12}$ homozygous mutant flies to wild-type *S. aureus* (Figure 5A). Nevertheless, the survival probability of homozygous double *PGRP-SA*^{seml}; *PGRP-LC*$^{\Delta E12}$ mutant flies was statistically distinct from (and reduced compared to) *PGRP-SA*^{seml} mutant flies, suggesting that PGRP-LC did contribute to the final sum of host survival following *S. aureus* infection (Figure 5A; see Tables S3 and S4 for statistics). This contribution became more evident when host survival following

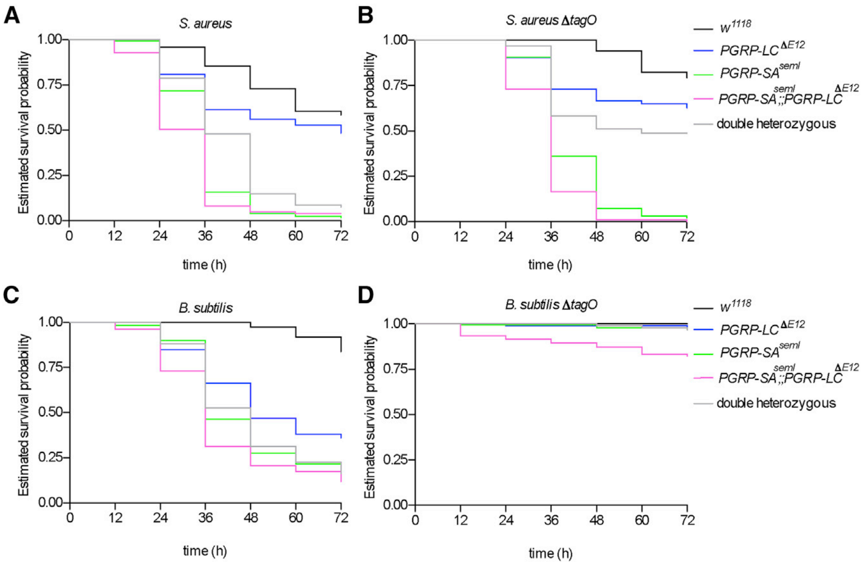

Figure 5. Survival Probability Patterns of Flies Deficient in PGRP-SA or PGRP-LC (or Both) following Infection with WTA-less Bacteria

(A) Following infection with *S. aureus*, there was a statistically significant difference in the contribution of survival to infection when comparing either *PGRP-SA*^{seml} or *PGRP-LC*$^{\Delta E12}$ single mutants with the wild-type control w^{1118} (which survived more often) or with the homozygous *PGRP-SA*^{seml}*; PGRP-LC*$^{\Delta E12}$ double mutant (which survived less often). However, PGRP-SA contributed more since *PGRP-SA*^{seml} was statistically closer to the double mutant.

(B) Infection with *S. aureusΔTagO* increased the statistical difference between PGRP-SA and *PGRP-SA*^{seml}*; PGRP-LC*$^{\Delta E12}$. This indicated the augmented participation of PGRP-LC in survival.

(C) After infection with *B. subtilis*, the survival probabilities of flies followed a pattern in which both PGRPs participated and the double homozygote mutant displayed the highest susceptibility, while w^{1118} displayed the lowest susceptibility.

(D) Nevertheless, removal of WTAs rendered both single mutants resistant to *B. subtilisΔTagO* as w^{1118} and the double heterozygote, whereas only the double homozygous mutant displayed a moderate susceptibility to infection.

The results shown here are from pooled survival data (n = 87–100 for each genotype) from three independent sets of injections (there were no significant departures between repeats; $p > 0.1$, log rank test) and plotted estimates of survival probabilities. To extract p values, populations were compared using log rank and Wilcoxon tests.

See also the statistics in Table S3.

S. aureusΔTagO was considered. Specifically, when comparing *PGRP-SA*^{seml} to *PGRP-SA*^{seml}; *PGRP-LC*$^{\Delta E12}$, the differences in survival probabilities were more distinct compared to wild-type *S. aureus* infection, and this was reflected in the greater statistically significant difference in susceptibility (Figure 5B; compare *PGRP-SA*^{seml} to *PGRP-SA*^{seml}; *PGRP-LC*$^{\Delta E12}$ in *S. aureus* and *S. aureusΔTagO* in Tables S3 and S4). This indicated that the role of PGRP-LC was more prominent in *S. aureusΔTagO* infection.

Infection by *B. subtilis* showed that both PGRP-SA and PGRP-LC contributed to survival, albeit with significant statistical differences. However, the double mutant was significantly more susceptible than either of the single mutants (Figure 5C; Tables S3 and S4). Following infection with *B. subtilisΔTagO*, the survival of both of the single mutants was statistically indistinguishable from the heterozygous double mutant and wild-type w^{1118} control exhibiting robust resistance to infection (Figure 5D; Tables S3 and S4). In contrast, the double *PGRP-SA*^{seml}; *PGRP-LC*$^{\Delta E12}$ homozygous mutant was statistically distinct and succumbed more easily to infection than either single mutant (Figure 5D; Tables S3 and S4). Of note, when injected with a sterile saline buffer (as a control for the injection itself), the survival of none of the PGRP mutants was different from the control (data not shown). In the host survival experiments described above, we used as controls the genetic background of the double mutant w^{1118} and the heterozygous siblings of the homozygous PGRP mutants. In all of the infections, the survival probabilities of the w^{1118} strain were the highest of all fly stocks challenged, the double mutant the lowest, and the double heterozygote in the center of the two (Figure S7).

Growth of WTA-less Bacteria Inside the Host Depends on Both PGRPs

To correlate fly survival with bacterial growth inside the host, survival results were combined with the measurements of pathogen growth in colony-forming units (CFUs). Our results are shown in Figure 6. The growth of both *ΔTagO* mutants was restricted in w^{1118} controls, in contrast to their parental strains (compare Figure 6A *S. aureus* and Figure 6B for *S. aureusΔTagO*; Figure 6C for *B. subtilis* and Figure 6D for *B. subtilisΔTagO*). The picture was different in PGRP mutants, in which growth of both parental bacterial strains (Figures 6A and 6B) and *ΔTagO* mutants increased through time (Figures 6B and 6D). However, the growth of *S. aureus* showed no difference between *PGRP-SA*^{seml} and *PGRP-SA*^{seml}; *PGRP-LC*$^{\Delta E12}$ double mutants until 36 h post-infection, indicating that PGRP-SA was the main restricting factor for wild-type *S. aureus*, and loss of PGRP-LC only added to the phenotype at later stages of infection (Figure 6A). Of note, *S. aureus* is a "fast killer," in which 40% of flies die in 72 h, with only 300 cells initial infection (Atilano et al., 2011).

However, *S. aureusΔTagO* growth showed increasing statistically significant differences between *PGRP-SA*^{seml}; *PGRP-LC*$^{\Delta E12}$ and *PGRP-SA*^{seml} mutants from 24 to 36 h post-infection (Figure 6B). This meant that when accessibility to cell wall PG was increased, both PGRPs were involved in restricting *S. aureus* at an earlier point in the infection.

The growth of *B. subtilis* was statistically indistinguishable between *PGRP-LC*$^{\Delta E12}$ and *PGRP-SA*^{seml}; *PGRP-LC*$^{\Delta E12}$ double mutants (Figure 6C). This was in contrast to *B. subtilisΔTagO* infection, in which there was a statistically significant increase in bacterial load in *PGRP-SA*^{seml}; *PGRP-LC*$^{\Delta E12}$ double mutants compared to *PGRP-LC*$^{\Delta E12}$ at both 24 and 36 h post-infection

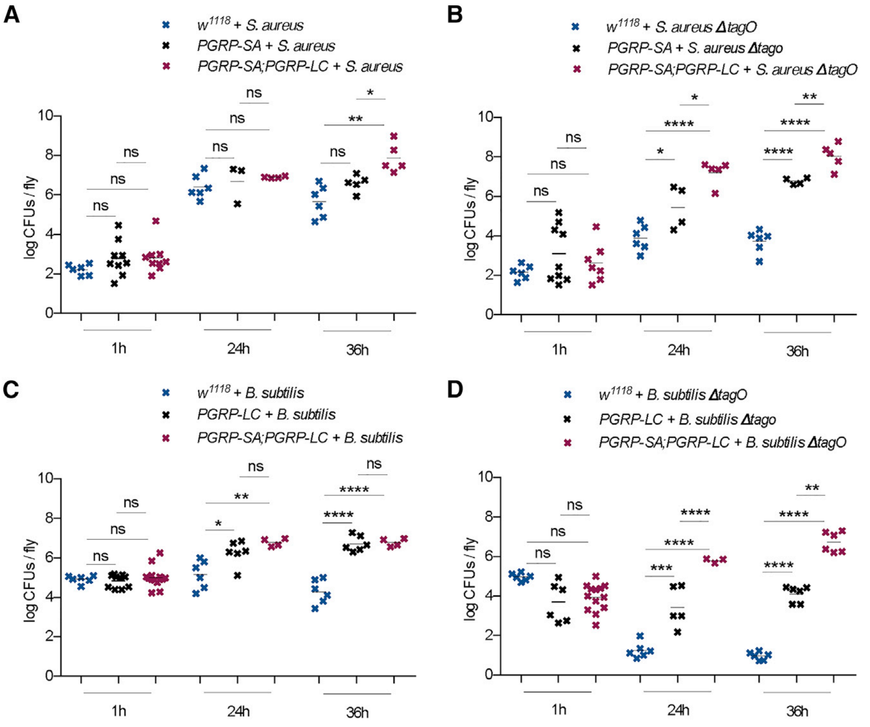

Figure 6. Growth of WTA-less *S. aureus* and *B. subtilis* Inside the Host Depend on Both PGRPs, in Contrast to Wild-Type Bacteria

(A) The growth of wild-type *S. aureus* was statistically indistinguishable between *PGRP-SA^{seml}* and *PGRP-SA^{seml}; PGRP-LC$^{\Delta E12}$* flies, except in the later stage of infection (36 h).

(B) *S. aureusΔTagO* in wild-type flies showed a significant difference in the dynamics of pathogen clearance compared to *S. aureus* wild type, with the *ΔTagO* mutant being cleared rather than restricted. However, *S. aureusΔTagO* was able to grow significantly more in *PGRP-SA^{seml}; PGRP-LC$^{\Delta E12}$* than in *PGRP-SA^{seml}* flies at 24 and 36 h, showing a dependence on both PGRPs.

(C) Growth of wild-type *B. subtilis* was dependent on PGRP-LC only, since bacterial growth in *PGRP-LC$^{\Delta E12}$* versus *PGRP-SA^{seml}; PGRP-LC$^{\Delta E12}$* was statistically indistinguishable.

(D) *B. subtilisΔTagO* grew better in *PGRP-SA^{seml}; PGRP-LC$^{\Delta E12}$* than in single *PGRP-LC$^{\Delta E12}$* mutant flies, indicating that both receptors participated in restricting growth when WTA was removed.

CFU comparisons were determined by Student's t test, in which *$p < 0.05$ and ****$p < 0.0001$ indicate significance value. ns, non-significant.

(Figure 6D). This indicated that when WTAs were removed from *B. subtilis*, not only PGRP-LC but also PGRP-SA started to play a role in restricting pathogen growth. Therefore, our CFU measurements indicated that both PGRPs were involved in the restriction of WTA-less bacteria, in contrast to the wild-type parental bacterial strains.

Infection with WTA-less Bacteria Abolishes Differential Antimicrobial Peptide Gene Expression

To trace signaling activation over time downstream of the two PGRPs following infection and ascertain whether the bindings we have observed led to the induction of immune activity, we measured AMP gene expression. As proxy for the PGRP-SA/Toll signaling pathway, we used the AMP gene *drosomycin* (*drs*), whereas as a proxy for PGRP-LC/IMD signaling pathway activity, we measured the induction of the AMP gene *diptericin* (*dipt*).

Infection of *w^{1118}* flies with *B. subtilis* induced a robust differential activation of antimicrobial peptide gene expression with almost 100× more *dipt* than *drs* at 48 h (Figure 7A; Figure S8A, AMP levels normalized with CFUs). In contrast, infection with *B. subtilisΔTagO* did not induce a differential response, indicating a more equal participation of the two PGRPs in inducing downstream signaling (Figures 7B and S8B). Infection with *S. aureus* triggered a robust *dipt* induction, which was 2× more than *drs*, contrary to expectations for an Lys-type PG bacterium (Figures 7C and S8C) but in keeping with the recent literature in *Drosophila* (Wu et al., 2012) and the Oriental fruit fly, *Bactrocera dorsalis* (Shi et al., 2017). In contrast, infection with *S. aureusΔTagO* induced statistically indistinguishable AMP levels, showing that when WTAs were removed, both PGRPs participated equally in AMP induction (Figure 7D; Figure S8D, AMP levels normalized with CFUs).

To ascertain that *drs* was indeed under PGRP-SA control and *dipt* under PGRP-LC control, we used the relevant fly mutants. In this context, *PGRP-SA^{seml}* mutants failed to induce *drs* following infection with both *B. subtilis* (Figure 7E) and *B. subtilisΔTagO* (Figure 7F) while *PGRP-LC$^{\Delta E12}$* flies did not induce *dipt* following both *S. aureus* (Figure 7G) and *S. aureusΔTagO* infection (Figure 7H).

These results showed that there was robust PGRP-LC-dependent *dipt* induction following *S. aureus* and *S. aureusTagO,* while there was also PGRP-SA-dependent induction of *drs* transcription following *B. subtilisTagO* challenge.

However, wild-type flies displayed lower AMP level induction after challenge with either *ΔTagO* mutant. The lower level of activation of the *ΔTagO* mutants was due to the higher rate of phagocytosis, which was taking these bacteria from circulation early (as supported by CFUs in Figure 6; see also Video S2).

Video S2 represents live imaging of *ex vivo* phagocytosis by moesin-GFP-labeled primary larval hemocytes. When infected with *S. aureus*, hemocytes started exhibiting the hallmarks of cell death (shrinking and karyorrhexis; see Kitanaka and Kuchino, 1999; Wlodkowic et al., 2011) at ~1:15 h post-infection (Video S2, up to 1 min:11 s). In contrast, infection with *S. aureusΔTagO* induced death at ~4:30 h, thus making more cells available for a longer time to engage in phagocytosis and pathogen clearance (Video S2, 1 min:12 s—2 min:13 s). *B. subtilis* induced the hallmarks of death much later as compared to *S. aureus* (at ~12:30 h post-infection) (Video S2, 2 min:14 s–3 min:23 s).

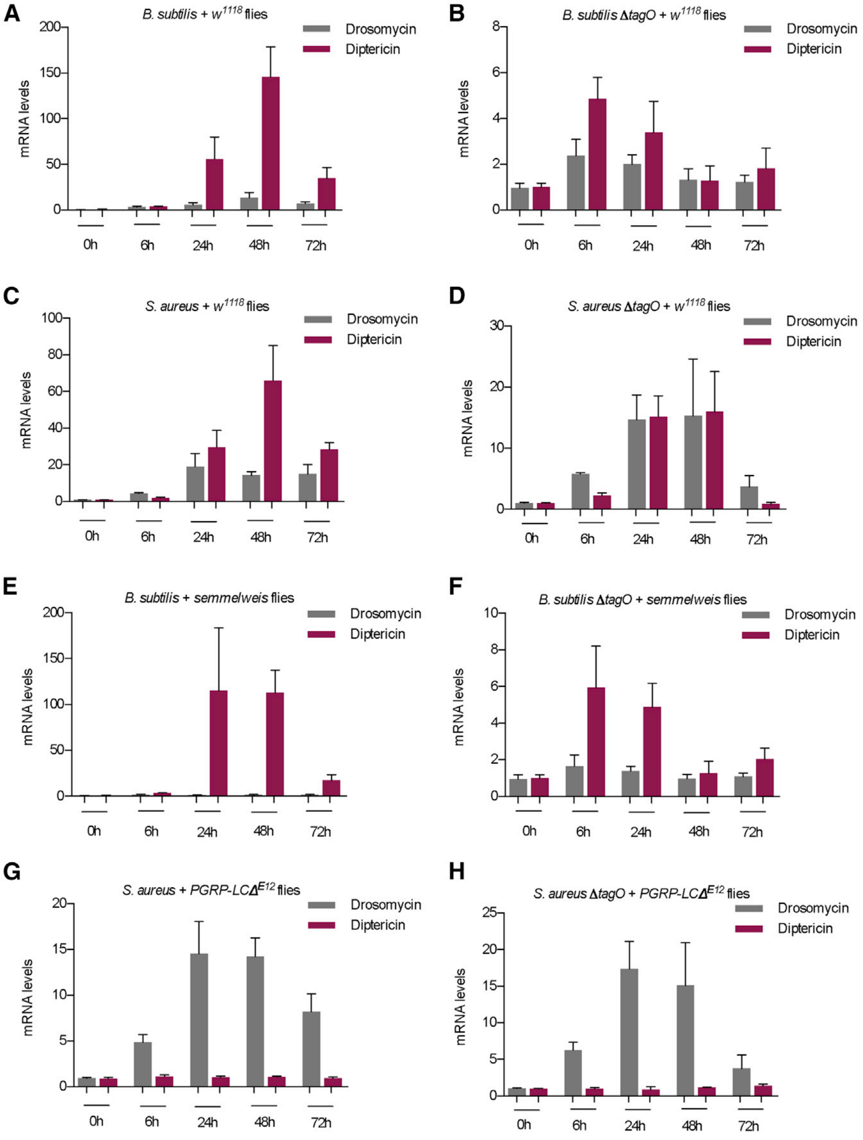

Figure 7. PGRP-SA- and PGRP-LC-Mediated Transcriptional Induction of AMPs Is Lower but More Equal after WTA-less Bacterial Infection

(A) *B. subtilis* induced a robust *dipt* induction almost 100× more than *drs*.

(B) In contrast, *B. subtilis⊿TagO* activated a lower but more equal AMP response, with the only significant difference observed at 6 h.

(C) *S. aureus* also induced a robust *dipt* activation 4–5× higher at 48 h compared to *drs*.

(D) *S. aureus⊿TagO* induced *dipt* and *drs* gene expression, which presented no statistical difference across the time points studied.

(E–H) To ascertain that *dipt* was induced by PGRP-LC following Lys-type infection and *drs* by PGRP-SA following DAP-type infection, we challenged *PGRP-SA*^{seml} mutants with *B. subtilis* (E) and *B. subtilis⊿TagO* (F) and verified that *drs* expression following DAP-type bacterial infection was dependent on PGRP-SA. Similarly, infecting *PGRP-LC*$^{\Delta E12}$ with *S. aureus* (G) or *S. aureus⊿TagO* (H) verified that the robust induction of *dipt* recorded was dependent on PGRP-LC.

However, *B. subtilis ⊿TagO* infection did not induce cellular death at all and was cleared (Video S2, 3 min:24 s–4 min:34 s), indicating again differences between parental strain and WTA-less bacteria. The outcome of infection with *B. subtilis ⊿TagO* infection was similar to hemocyte primary culture without infection, in which cells were able to survive an 18-h observation period *ex vivo* in Schneider's medium (Video S2, 4 min:35 s–5 min:44 s). Similarly, infection with heat-killed *S. aureus* did not induce hemocyte death, and host macrophages were able to contain the infection (Video S2, 5 min:45 s–6 min:56 s). Our working hypothesis is that prolonged survival of hemocytes in infections with WTA-less bacteria would increase bacterial uptake by macrophages, which are the first line of defense. These would leave much less bacteria to activate AMP responses that are typically induced much later during the timeline of the response to infection (Shia et al., 2009). Thus, AMP levels were lower in WTA-less infection.

Nevertheless, bacteria in WTA-less infections triggered per CFU equally PGRP-SA-dependent *drs* and PGRP-LC-dependent *dipt*, in contrast to the parental bacterial strains that activated per CFU differential AMP transcription.

DISCUSSION

In *Drosophila,* the generally accepted explanation for selective activation of immune pathways at the recognition level has been that PGRPs structurally discriminate between different types of PGs. This discrimination occurs on the basis of the amino acid present at position 3 of the stem peptide. Thus, PGRP-SA recognizes Lys (found in Gram-positive bacteria), while PGRP-LC interacts with DAP (found in Gram-negative bacteria and Gram-positive bacilli). The idea of structural discrimination at the level of the stem peptide resulted from a combination of observations, including PG binding assays, structural work, and *in vivo* infection experiments. However, there are several concerns with the experiments that support this Lys/DAP dichotomy or with the interpretation of the data. A summary of these follows.

First, the PG binding experiments were conducted with a buffer that is often used to solubilize proteins, but that was very different from hemolymph (where interactions between PG and PGRPs take place).

Second, the PG used for binding as well as infection experiments was quantified only by weight and not by the number of disaccharide GlcNAc-MurNAC added to the binding reaction.

Third, the structural data available and previously described referred to the binding of monomeric PG, whereas PGRP-SA

and PGRP-LCx are both able to bind polymeric PG and may do so *in vivo*. In the context of polymeric PG binding, the small difference of a carboxyl group on the Cε with D-chirality in DAP may not be as important for downstream signaling. Moreover, superposition of the two structures showed that the electrostatic potential at the surface of both PGRPs suggests that binding to both types of PG in solution is probable.

Based on the published structure (Chang et al., 2006), the defining characteristic of the binding groove is the conformation of Arg413, which securely anchors DAP-type PG and is likely to impose a preference for the latter via increased conformational stability (Figure S6D). This preference was verified with quantitative binding to Lys and DAP-type PG, in which the latter was bound in increased quantities by PGRP-LC (Figure S6C; Table S2). Nevertheless, PGRP-LC did bind Lys-type PG *in vitro*. Moreover, binding of PGRP-LC to Lys-type bacteria was recorded *ex vivo*, and this binding resulted in a robust AMP induction that was statistically higher than the one elicited through PGRP-SA, considered the bona fide receptor for Gram-positive bacteria (Figure 7C). This indicated that the upstream binding preferences of PGRP-LC did not result in differences in transcriptional AMP induction.

For PGRP-SA, replacement of the anchoring Arg413 from PGRP-LC with a threonine should leave an accommodating void that can flexibly bind via a water molecule network either types of PG (Figure S6F). In this case, there should be no noticeable difference in binding affinity. We found little difference between PGRP-SA binding to Lys PG compared with DAP-type PG (Figures 4C and S5B; Table S1).

Our results showed that when WTAs were removed, the binding of PGRPs to whole bacteria was significantly improved. This, however, was not reflected in binding assays to purified PG, showing that the increase in binding was about accessibility to PG on the bacterial cell wall, not about changing the WTA-less PG itself. This is consistent with the fact that purified PG from WTA-less bacteria and their parental strains had no significant differences in their HPLC profile analysis that could lead to an improved binding of PGRP-SA or PGRP-LC (Atilano et al., 2010).

The above results show that when WTAs were removed from bacteria, the majority of the observed phenotype attributed to selective recognition and downstream signaling based on the bacterial type were either abolished or diminished. This was accompanied by PGRP binding to whole bacteria, irrespective of PG type, in which both PGRPs played a role in host survival and restricting pathogen growth. Our results are in conflict with the general consensus of Lys/DAP discrimination and put forward an alternative explanation for differential immune triggering. We propose that at least for bacteria that have their PGs exposed to the host environment (Gram-positive bacteria and bacilli), accessibility to PG is a major factor restricting binding, recognition, and downstream signaling. Structural discrimination may still be important when monomeric PG fragments are the only means of “accessing” PG (in Gram-negative bacteria).

A fundamental issue when considering pathogen recognition in innate immunity is how a small number of germline coded receptors sense the vast variability of bacterial pathogens. Adapting receptors to an evolutionary conserved bacterial molecule essential for bacterial survival but not present in the host (e.g., PG), has been considered a productive host strategy. However, we believe that it would have been detrimental for the host to further specialize its non-enzymatic PGRP receptors to PG-stem peptide variants, as this would essentially decrease even more the effective number of possible productive host-pathogen recognition interactions, especially in an animal such as *Drosophila,* challenged by a wide variety of pathogens, each of which interacts rarely with it (Jiggins and Hurst, 2003). Put it another way, *Drosophila* is not subject to the arms race-type tight co-evolution with its pathogens that *Anopheles* is, for example, but it is subject to diffuse co-evolution with an array of non-specialist pathogens (Janzen, 1980; Jiggins and Hurst, 2003).

Evolutionary studies on *Drosophila* PGRPs (including SA and LC) have shown that there is no strong co-evolution interaction, and as such, no need for specialization (Jiggins and Hurst, 2003). We propose that it was the accessibility to PGs on different bacterial cell wall structures that defined how PGRPs were able to bind to PG on whole bacteria and the specified differences in downstream signaling responses, instead of direct structural specialization, as previous studies suggested. Our results indicate that even when structural preference exists (as in the case of PGRP-LC), differences in downstream signaling are equalized when accessibility to PG on the cell wall is increased. More work is needed to test whether accessibility is also critical for PGRP-mediated recognition of whole bacteria in mammals.

STAR★METHODS

Detailed methods are provided in the online version of this paper and include the following:

- KEY RESOURCES TABLE
- CONTACT FOR REAGENTS AND RESOURCES SHARING
- EXPERIMENTAL MODEL AND SUBJECT DETAILS
  - Bacterial strains and culture
  - Fly Strains
- METHOD DETAILS
  - Plasmids
  - PGRP-SA^{seml}; PGRP-LC$^{\Delta E12}$ double mutant
  - Hemolymph-like buffer
  - Purification of recombinant PGRP-SA and PGRP-LC proteins
  - Construction of mCherry_PGRP-SA mutant proteins
  - Purification of Peptidoglycan
  - Binding of PGRPs to whole bacteria and imaging
  - Quantification of PG using HPAEC-PAD
  - Hydrolysis of PG into its basic constituents and separation of the monosaccharides
  - Control samples for PG purification
  - Controls for quantification of Glucosamine and Muramic acid
  - Quantification of Glucosamine and Muramic acid
  - PG-PGRP co-precipitation assays
  - Live bacteria co-precipitation assays
  - Quantification of binding of fluorescent PGRPs to bacterial cells
  - Survivals and CFUs

- ○ Measurements of gene expression in whole flies
- ○ Time-lapse microscopy
- ● QUANTIFICATION AND STATISTICAL ANALYSIS

SUPPLEMENTAL INFORMATION

Supplemental Information can be found online at https://doi.org/10.1016/j.celrep.2019.04.103.

ACKNOWLEDGMENTS

We would like to thank Bruno Lemaitre and the Bloomington Stock Centre for fly stocks and Oxford MICRON for expert help with time-lapse imaging. We are grateful for support by the European Research Council Consolidator Grant 310912 and BBSRC Project Grant BB/P005691/1 (both to P.L.); PhD fellowships SFRH/BD/52207/2013 (to G.C.) and SFRH/BD/78748/2011 (to F.V.) from the Portuguese Science Agency (FCT); ONEIDA project LISBOA-01-0145-FEDER-016417; and grants PTDC/BIA-PLA/3432/2012 and IF/01464/2013 (to S.R.F.). Thanks to Andrew Jefferson and MICRON (http://micronoxford.com), supported by Wellcome Trust Strategic Awards 091911/B/10/Z and 107457/Z/15/Z, for access to equipment and assistance with imaging techniques.

AUTHOR CONTRIBUTIONS

P.L. and S.R.F. conceived the experiments and obtained funding. I.D. obtained funding for MICRON. F.V., G.C., and I.K. performed the experiments. R.M.P. and F.V. performed the time-lapse experiments. M.H. performed the re-evaluation of the available structural data. F.V., G.C., and I.K. performed the data analysis, with input from P.L. and S.R.F. P.L. wrote the manuscript, with input from S.R.F. and I.D.

DECLARATION OF INTERESTS

The authors declare no competing interests.

Received: June 14, 2017
Revised: March 19, 2019
Accepted: April 23, 2019
Published: May 21, 2019

REFERENCES

Atilano, M.L., Pereira, P.M., Yates, J., Reed, P., Veiga, H., Pinho, M.G., and Filipe, S.R. (2010). Teichoic acids are temporal and spatial regulators of peptidoglycan cross-linking in Staphylococcus aureus. Proc. Natl. Acad. Sci. USA *107*, 18991–18996.

Atilano, M.L., Yates, J., Glittenberg, M., Filipe, S.R., and Ligoxygakis, P. (2011). Wall teichoic acids of *Staphylococcus aureus* limit recognition by the *drosophila* peptidoglycan recognition protein-SA to promote pathogenicity. PLoS Pathog. *7*, e1002421.

Atilano, M.L., Pereira, P.M., Vaz, F., Catalão, M.J., Reed, P., Grilo, I.R., Sobral, R.G., Ligoxygakis, P., Pinho, M.G., and Filipe, S.R. (2014). Bacterial autolysins trim cell surface peptidoglycan to prevent detection by the Drosophila innate immune system. eLife *3*, e02277.

Chang, C.-I., Pili-Floury, S., Hervé, M., Parquet, C., Chelliah, Y., Lemaitre, B., Mengin-Lecreulx, D., and Deisenhofer, J. (2004). A *Drosophila* pattern recognition receptor contains a peptidoglycan docking groove and unusual L,D-carboxypeptidase activity. PLoS Biol. *2*, E277.

Chang, C.-I., Chelliah, Y., Borek, D., Mengin-Lecreulx, D., and Deisenhofer, J. (2006). Structure of tracheal cytotoxin in complex with a heterodimeric pattern-recognition receptor. Science *311*, 1761–1764.

Covas, G., Vaz, F., Henriques, G., Pinho, M.G., and Filipe, S.R. (2016). Analysis of cell wall teichoic acids in *Staphylococcus aureus*. Methods Mol. Biol. *1440*, 201–213.

Croghan, P.C., and Lockwood, A.P.M. (1960). The composition of the haemolymph of the larva of *Drosophila melanogaster*. J. Exp. Biol. *37*, 339–343.

D'Elia, M.A., Millar, K.E., Beveridge, T.J., and Brown, E.D. (2006). Wall teichoic acid polymers are dispensable for cell viability in Bacillus subtilis. J. Bacteriol. *188*, 8313–8316.

De Gregorio, E., Spellman, P.T., Tzou, P., Rubin, G.M., and Lemaitre, B. (2002). The Toll and Imd pathways are the major regulators of the immune response in *Drosophila*. EMBO J. *21*, 2568–2579.

Edwards, K.A., Demsky, M., Montague, R.A., Weymouth, N., and Kiehart, D.P. (1997). GFP-moesin illuminates actin cytoskeleton dynamics in living tissue and demonstrates cell shape changes during morphogenesis in Drosophila. Dev. Biol. *191*, 103–117.

Filipe, S.R., Tomasz, A., and Ligoxygakis, P. (2005). Requirements of peptidoglycan structure that allow detection by the Drosophila Toll pathway. EMBO Rep. *6*, 327–333.

Gottar, M., Gobert, V., Michel, T., Belvin, M., Duyk, G., Hoffmann, J.A., Ferrandon, D., and Royet, J. (2002). The *Drosophila* immune response against Gram-negative bacteria is mediated by a peptidoglycan recognition protein. Nature *416*, 640–644.

Guan, R., Roychowdhury, A., Ember, B., Kumar, S., Boons, G.-J., and Mariuzza, R.A. (2004). Structural basis for peptidoglycan binding by peptidoglycan recognition proteins. Proc. Natl. Acad. Sci. USA *101*, 17168–17173.

Janzen, D.H. (1980). When is it coevolution? Evolution *34*, 611–612.

Jiggins, F.M., and Hurst, G.D.D. (2003). The evolution of parasite recognition genes in the innate immune system: purifying selection on *Drosophila melanogaster* peptidoglycan recognition proteins. J. Mol. Evol. *57*, 598–605.

Kaneko, T., Goldman, W.E., Mellroth, P., Steiner, H., Fukase, K., Kusumoto, S., Harley, W., Fox, A., Golenbock, D., and Silverman, N. (2004). Monomeric and polymeric gram-negative peptidoglycan but not purified LPS stimulate the *Drosophila* IMD pathway. Immunity *20*, 637–649.

Kervann, C., and Boulanger, D. (2008). J. Local adaptivity to variable smoothness for exemplar-based image regularization and representation. Int. J. Comput. Vis. *79*, 45–69.

Kitanaka, C., and Kuchino, Y. (1999). Caspase-independent programmed cell death with necrotic morphology. Cell Death Differ. *6*, 508–515.

Kounatidis, I., and Ligoxygakis, P. (2012). *Drosophila* as a model system to unravel the layers of innate immunity to infection. Open Biol. *2*, 120075.

Lemaitre, B., Reichhart, J.M., and Hoffmann, J.A. (1997). *Drosophila* host defense: differential induction of antimicrobial peptide genes after infection by various classes of microorganisms. Proc. Natl. Acad. Sci. USA *94*, 14614–14619.

Leone, P., Bischoff, V., Kellenberger, C., Hetru, C., Royet, J., and Roussel, A. (2008). Crystal structure of Drosophila PGRP-SD suggests binding to DAP-type but not lysine-type peptidoglycan. Mol. Immunol. *45*, 2521–2530.

Leulier, F., Parquet, C., Pili-Floury, S., Ryu, J.-H., Caroff, M., Lee, W.J., Mengin-Lecreulx, D., and Lemaitre, B. (2003). The *Drosophila* immune system detects bacteria through specific peptidoglycan recognition. Nat. Immunol. *4*, 478–484.

Medzhitov, R., and Janeway, C.A., Jr. (1997). Innate immunity: the virtues of a nonclonal system of recognition. Cell *91*, 295–298.

Mellroth, P., Karlsson, J., Håkansson, J., Schultz, N., Goldman, W.E., and Steiner, H. (2005). Ligand-induced dimerization of *Drosophila* peptidoglycan recognition proteins *in vitro*. Proc. Natl. Acad. Sci. USA *102*, 6455–6460.

Michel, T., Reichhart, J.M., Hoffmann, J.A., and Royet, J. (2001). Drosophila Toll is activated by Gram-positive bacteria through a circulating peptidoglycan recognition protein. Nature *414*, 756–759.

Neyen, C., Poidevin, M., Roussel, A., and Lemaitre, B. (2012). Tissue- and ligand-specific sensing of gram-negative infection in Drosophila by PGRP-LC isoforms and PGRP-LE. J. Immunol. *189*, 1886–1897.

Park, J.W., Kim, C.H., Kim, J.H., Je, B.R., Roh, K.B., Kim, S.J., Lee, H.H., Ryu, J.H., Lim, J.H., Oh, B.H., et al. (2007). Clustering of peptidoglycan recognition

protein-SA is required for sensing lysine-type peptidoglycan in insects. Proc. Natl. Acad. Sci. USA *104*, 6602–6607.

Parton, R.M., Valles, A.M., Dobbie, I.M., and Davis, I. (2010). Pushing the limits of live cell imaging in *Drosophila*. In Live Cell Imaging: A Laboratory Manual, 2nd, R.D. Goldman and D.L. Spector, eds. (Cold Spring Harbor Laboratory Press), pp. 387–418.

Reiser, J.-B., Teyton, L., and Wilson, I.A. (2004). Crystal structure of the Drosophila peptidoglycan recognition protein (PGRP)-SA at 1.56 A resolution. J. Mol. Biol. *340*, 909–917.

Royet, J., and Dziarski, R. (2007). Peptidoglycan recognition proteins: pleiotropic sensors and effectors of antimicrobial defences. Nat. Rev. Microbiol. *5*, 264–277.

Schleifer, K.H., and Kandler, O. (1972). Peptidoglycan types of bacterial cell walls and their taxonomic implications. Bacteriol. Rev. *36*, 407–477.

Schmidt, R.L., Rinaldo, F.M., Hesse, S.E., Hamada, M., Ortiz, Z., Beleford, D.T., Page-McCaw, A., Platt, J.L., and Tang, A.H. (2011). Cleavage of PGRP-LC receptor in the *Drosophila* IMD pathway in response to live bacterial infection in S2 cells. Self Nonself *2*, 125–141.

Shi, Z., Liang, H., and Hou, Y. (2017). Functional analysis of a NF-κB transcription factor in the immune defense of Oriental fruit fly, *Bactrocera dorsalis Hendel* (Diptera: Tephritidae). Bull. Entomol. Res. *107*, 251–260.

Shia, A.K.H., Glittenberg, M., Thompson, G., Weber, A.N., Reichhart, J.M., and Ligoxygakis, P. (2009). Toll-dependent antimicrobial responses in Drosophila larval fat body require Spätzle secreted by haemocytes. J. Cell Sci. *122*, 4505–4515.

Sinenko, S.A., and Mathey-Prevot, B. (2004). Increased expression of *Drosophila* tetraspanin, Tsp68C, suppresses the abnormal proliferation of ytr-deficient and Ras/Raf-activated hemocytes. Oncogene *23*, 9120–9128.

Swaminathan, C.P., Brown, P.H., Roychowdhury, A., Wang, Q., Guan, R., Silverman, N., Goldman, W.E., Boons, G.J., and Mariuzza, R.A. (2006). Dual strategies for peptidoglycan discrimination by peptidoglycan recognition proteins (PGRPs). Proc. Natl. Acad. Sci. USA *103*, 684–689.

Takehana, A., Katsuyama, T., Yano, T., Oshima, Y., Takada, H., Aigaki, T., and Kurata, S. (2002). Overexpression of a pattern-recognition receptor, peptidoglycan-recognition protein-LE, activates imd/relish-mediated antibacterial defense and the prophenoloxidase cascade in Drosophila larvae. Proc. Natl. Acad. Sci. USA *99*, 13705–13710.

van der Meer, J.M., and Jaffe, L.F. (1983). Elemental composition of the perivitelline fluid in Drosophila embryos. Dev. Biol. *95*, 249–252.

Wlodkowic, D., Telford, W., Skommer, J., and Darzynkiewicz, Z. (2011). Apoptosis and beyond: cytometry in studies of programmed cell death. Methods Cell Biol. *103*, 55–98.

Wu, K., Conly, J., Surette, M., Sibley, C., Elsayed, S., and Zhang, K. (2012). Assessment of virulence diversity of methicillin-resistant *Staphylococcus aureus* strains with a *Drosophila melanogaster* infection model. BMC Microbiol. *12*, 274–279.

Yu, Y., Park, J.-W., Kwon, H.-M., Hwang, H.-O., Jang, I.-H., Masuda, A., Kurokawa, K., Nakayama, H., Lee, W.J., Dohmae, N., et al. (2010). Diversity of innate immune recognition mechanism for bacterial polymeric *meso*-diaminopimelic acid-type peptidoglycan in insects. J. Biol. Chem. *285*, 32937–32945.

STAR★METHODS

KEY RESOURCES TABLE

REAGENT or RESOURCE	SOURCE	IDENTIFIER
Bacterial Strains		
E. coli DH5α	Invitrogen	Cat No.11319019
E. coli BL21 (DE3)	Invitrogen	Cat No.C600003
B. subtilis	D'Elia et al., 2006	EB6
*B. subtilis*Δ*tagO*	D'Elia et al., 2006	EB1451
S. aureus	From R. Novick	NCTC8325-4
*S. aureus*Δ*tagO*	Atilano et al., 2011	NCTC8325-4 *tagO* null
Chemicals, Peptides, and Recombinant Proteins		
Hemolymph-like buffer (HemoBuffer)	This study	N/A
Fetal Calf Serum	Sigma	Cat. No F2442
Schneider's medium	GIBCO	Cat No. 21720024
HCl	Sigma	Cat No. 7647-01-0
Ribitol	Chem Scene	Cat No. 488-81-3
Glycerol	Sigma	Cat No. 56-81-5
Mannosamine	Sigma	Cat No. M4670
Critical Commercial Assays		
Maxima First Strand cDNA synthesis kit	Thermo Scientific	Cat No. K1651
SensiFASAT SYBR No-ROX kit	Bioline	Cat No. BIO-98005
Total RNA Purification Plus kit	Norgen Biotek	Cat No. 48200
Wizard PCR Clean Up kit	Promega	Cat No. A9281
Experimental Models: Organisms/Strains		
PGRP-SA^{seml}	Michel et al., 2001	N/A
PGRP-LC $^{\Delta E12}$	Gottar et al., 2002	N/A
PGRP-SA^{seml}; PGRP-LC $^{\Delta E12}$	This study	N/A
w; UAS-GFP::PGRP-LCx	Schmidt et al., 2011	Lemaitre Lab
Moesin-GFP	Edwards et al., 1997	N/A
w[1118]; P{w[+mC] = Hml-GAL4.Delta}2	Sinenko and Mathey-Prevot 2004	Stock No. 30139
TI{w[+mW.hs] = TI}Pis[1]/FM7a;P{w[+mC] = hs-Pis.MYC}3/TM2	Bloomington Stock Centre	Stock No. 27336
Oligonucleotides		
PGRP-LCx cloning from IP15793 to pET21α: GGGAAGG GAATTCAACCAAACGGACTTGGATG" (forward)	This study	N/A
PGRP-LCx cloning from IP15793 to pET21α: TGCGG CCGCAAGCTTTTAGTGATGGTGATGGTGATGGA TTTCGTGTGACCAGTGCG" (reverse)	This study	N/A
rp49 forward: AAGAAGCGCACCAAGCACTTCATC	This study	N/A
rp49 reverse: TCTGTTGTCGATACCCTTGGGCTT	This study	N/A
diptericin forward: ACCGCAGTACCCACTCAATC	This study	N/A
diptericin reverse: GGTCCACACCTTCTGGTGAC	This study	N/A
drosomycin forward: AGTACTTGTTCGCCCTCTTCGCTG	This study	N/A
drosomycin reverse: CCTTGTATCTTCCGGACAGGCAGT	This study	N/A
Recombinant DNA		
PGRP-LCx isoform plasmid	DGRC	IP15793
pET21α	Novagen/Merck	Cat No 69740

(*Continued on next page*)

Continued		
REAGENT or RESOURCE	SOURCE	IDENTIFIER
pET21α-m-Cherry-PGRP-LCx	This study	N/A
pET21α-m-Cherry-PGRP-SA	Atilano et al., 2011	N/A
Software and Algorithms		
ImageJ	NIH	https://imagej.en.softonic.com/
PyMol	https://pymol.org/2/	https://pymol.org/2/
GraphPad Prism 6	GraphPad Software	https://www.graphpad.com/
SoftWoRx	GE Healthcare	http://incelldownload.gehealthcare.com/bin/download_data/SoftWoRx/7.0.0/SoftWoRx.htm
Other		
Quantification of Peptidoglycan using HPAEC-PAD	This study	N/A

CONTACT FOR REAGENTS AND RESOURCES SHARING

Further information and requests for resources reagents should be directed to and will be fulfilled by the Lead Contact, Petros Ligoxygakis (petros.ligoxygakis@bioch.ox.ac.uk).

EXPERIMENTAL MODEL AND SUBJECT DETAILS

Bacterial strains and culture

E. coli strains DH5α and BL21(DE3) (both for construction and expression of the recombinant proteins), *B. subtilis* EB6 and EB6Δ*tagO* (EB1451) strains (D'Elia et al., 2006) were grown either in Luria–Bertani broth (LB; Difco, France) or in Luria–Bertani agar (LA; Difco). *S. aureus* strains NCTC 8325-4 (reference strain, kind gift from R. Novick) and NCTC Δ *tagO* (Atilano et al., 2011) were grown either in Tryptic Soy broth (TSB; Difco) or in Tryptic Soy agar (TSA; Difco). All cultures were grown at 30°C with aeration, except when infected flies were crushed and plated for CFUs, the LA and TSA plates were incubated at 25°C.

Fly Strains

All flies, stocks and crosses, were grown on standard cornmeal-agar medium at 25°C. We used PGRP-SA^{seml} (Michel et al., 2001), *PGRP-LC*$^{\Delta E12}$ (Gottar et al., 2002), balancer stock Bl#27336 flies (FBst0027336 from Bloomington *Drosophila* Stock Centre), *w*; UAS-GFP::PGRP-LCx (Schmidt et al., 2011), Moesin-GFP (Edwards et al., 1997) and Hml$^{\Delta}$Gal4 (Sinenko and Mathey-Prevot, 2004).

METHOD DETAILS

Plasmids

Plasmid IP15793 (*Drosophila* Genomics Resource Centre) containing the PGRP-LCx isoform, was used to amplify the PGRP domain (region 966 bp to1500 bp of the CDS) with the pair of primers (sequences from 5' to 3'): "GGGAAGGGAATTCAACCAAACGGACTTG GATG" (forward) and

"TGCGGCCGCAAGCTTTTAGTGATGGTGATGGTGATGGATTTCGTGTGACCAGTGCG" (reverse). Subsequently, the fragment was cloned into the EcoRI and EagI restriction sites of the pET21a backbones of the mCherry_PGRP-SA and rPGRP-SA vectors (Atilano et al., 2011). A 6x His-tag was added to the last coding triplet, before the stop codon, similar to the PGRP-SA constructs.

PGRP-SA^{seml}; PGRP-LC$^{\Delta E12}$ double mutant

For construction of the double mutant PGRP-SA^{seml}; PGRP-LC$^{\Delta E12}$ I and III chromosome of *semmelweis* flies and *PGRP-LC*$^{\Delta E12}$ flies were balanced through cross with. For each F1 of these two independent crosses, flies with both balancers were selected and the cross between female virgins *seml/FM7* and *TM2/+* with males *FM7/y* and *TM2/PGRP-LC*$^{\Delta E12}$ was set. Of this progeny, we selected female virgin flies that phenotypically presented both balancers and male flies that were not FM7 (hence were *seml* genotype) but presented TM2 phenotype.

Several single crosses between such females and males were set. After confirming the existence and viability of 1st instar larvae, the parents were removed and checked for both the *seml* mutation as well as for *PGRP-LC*$^{\Delta E12}$. Briefly, gDNA was extracted and the *PGRP-LC*$^{\Delta E12}$ was amplified by PCR using the primers "CACACGCTGCCATATCAGAC" and "TATCGGTTTTCCTGGGTGAG" (Neyen et al., 2012). The corresponding PCR *PGRP-LC*$^{\Delta E12}$ fragment should show a 212 bp band. Only the single crosses of which both female and male showed such amplification were kept and these PCR products along with the PCR products using the forward primer "TTAGATCTTAGCACATCAACATC" and reverse primer "GACTACTGCAATTACTTGTAGTTG" (sequences from 5' to 3') for

amplification of the *semmelweis/PGRP-SA* gene were sent to sequencing to confirm the genotypes. Females and males that did not present balancers, i.e., homozygous for both *semmelweis* and *PGRP-LC*$^{\Delta E12}$ genes were collected. Finally, single crosses between one of these females and one male were set to obtain a homogeneous population of homozygous flies for *seml* and *PGRP-LC*$^{\Delta E12}$. Three different lines from three different single crosses were obtained, of which both the parents and progeny genotype was confirmed by PCR and sequencing. Results obtained from survival and CFU studies were similar for all lines tested. For construction of the single mutants, the respective mutant flies balanced with the Bl#27336 flies were used and a similar approach as for the double mutant was followed. Again, three stocks for each single mutant, with one female and one male as progenitor parents for the whole stock were established. This was to ensure the *PGRP-SA*^{seml}, *PGRP-LC*$^{\Delta E12}$ and the double mutant, were in a similar genetic background. Results obtained from survival and CFU studies were similar for all lines tested.

Hemolymph-like buffer

This buffer (referred to in the text as HemoBuffer) was prepared according to previous studies (Croghan and Lockwood, 1960; Van Der Meer and Jaffe, 1983, see below on how HemoBuffer compares with these two publications). The suggested salts to prepare for the HemoBuffer are as follows (final concentration): $Na_2SO_4.10H_2O$ (7.3mM), $NaH_2PO_4.H_2O$ (34mM), $Na_2HPO_4.7H_2O$ (5mM), $CaCl_2.2H_2O$* (2mM), $MgCl_2.6H_2O$* (14.4mM), KCl (25mM). Please note that the final solution has a short shelf-life before precipitation occurs. We found that the easiest way to prepare this solution was to use the salt concentrations as above to prepare the final HemoBuffer solution. Furthermore, we found that $CaCl_2.2H_2O$ and $MgCl_2.6H_2O$ salts precipitate the solution if added together with the rest of the salts. Therefore, we found it useful to prepare a 10x concentration Stock Solution of HemoBuffer without $CaCl_2.2H_2O$ and $MgCl_2.6H_2O$ and prepare a fresh HemoBuffer solution by dilution and adding the $CaCl_2.2H_2O$ and $MgCl_2.6H_2O$ to the desired concentration.

Ion/ Concentration (mM)	Croghan and Lockwood, 1960	Van Der Meer and Jaffe, 1983	HemoBuffer pH 6.0
K^+	**25**	**24**	**25**
Na^+	**106**	**87**	**58.6**
Mg^{2+}	**14,4**	**26**	**14.4**
Ca^{2+}	**7,2**	**10,6**	**2**
Cl^-	**58**	**-**	**57.8**
PO_4^{2-}	**39**	**-**	**39**
SO_4^{2-}	**7,3**	**-**	**7.3**

Purification of recombinant PGRP-SA and PGRP-LC proteins

Recombinant proteins PGRP-SA and PGRP-LC with and without the mCherry fluorescent tag were purified using a protocol adapted from Atilano et al., 2011, 2014.

Construction of mCherry_PGRP-SA mutant proteins

The mCherry_PGRP-SA vector (Atilano et al., 2011) was used as a template to perform the following mutations: C54Y, also known as the PGRP-SA^{seml} mutation (Michel et al., 2001), and C48YC54Y. The positions denoted in the nomenclature refer to the position of the mutated amino acid residue in the primary sequence of the native protein that lacks the signal peptide. Mutants were constructed by Site-Directed Mutagenesis PCR with appropriate primers, followed by DpnI digestion at 37°C. Amplified PCR products were cleaned using Wizard PCR Clean Up kit (Promega) and transformed into DH5α competent cells. Following confirmation of the sequence present in the transformed plasmids, these were transformed into BL21a (DE3) *E. coli* strain, which was used as an expression system. Proteins were then purified as previously described (Atilano et al., 2011).

Purification of Peptidoglycan

The cell walls and PGs of *S. aureus* and *B. subtilis* parental and mutant strains were purified and analyzed as reported previously (Filipe et al., 2005).

Binding of PGRPs to whole bacteria and imaging

was performed as in Atilano et al., 2011.

Quantification of PG using HPAEC-PAD

Sugar components of the purified PGNs were analyzed using HPAEC-PAD (High Performance Anion Exchange Chromatography coupled with Pulsed Amperometric Detection). After quantification of the Glucosamine and Muramic acid in each PGN sample, the final PGN suspensions were adjusted to the same amount of Muramic acid for further studies.

Hydrolysis of PG into its basic constituents and separation of the monosaccharides

Three repeats for each PGN sample and three different batches of quantification were performed because as PGN is insoluble in aqueous solution, these repeats minimize potential errors in the quantification. The pure PGN suspension was left O/N stirring at RT for maximum homogenization. 20 μL of the suspensions were hydrolysed in 3 M HCl at 95°C for 2 h (150 μL reaction volume). The hydrolysed suspension was lyophilized until it was completely dried and resuspended in 500 μL of MilliQ H_2O and lyophilized O/N. Finally, the acid-free hydrolysed material was resuspended in 150 μL MilliQ H_2O.

For monosaccharide separation 10 μL of the samples were injected in a Thermo Scientific Dionex ICS-5000 system, in 18 mM NaOH constant and a gradient of 1 M $NaCH_3COO$ and MilliQ H_2O. The MilliQ H_2O as all the eluents were in MilliQ H_2O of resistivity $\geq$ 18 MΩ, filtered with 0.2 μm ϕ pore filter and degassed for 15 min in an ultrasonic bath. Between the eluents pump and the injection valve it was used a BorateTrap^{TM} column to remove borate contamination from eluents. An AminoTrap^{TM} (Thermo Scientific Dionex AminoTrap) was used as a pre-treatment column to remove the amino acids from the samples, thus only the sugars passed to the CarboPac PA10 column (Thermo Scientific CarboPac PA10) where they were separated. Each injection was done in triplicates.

Control samples for PG purification

Controls for HCl-mediated PG hydrolysis

To assess if hydrolysis had been complete, it was injected non-HCl treated Glucosamine, Muramic acid, *N*-acetylglucosamine and *N*-acetylmuramic acid. If the hydrolysis was complete, only Glucosamine and Muramic acid should be detected because the *N*-acetyl groups were removed by the acid treatment. Also these same samples HCl treated were injected, to determine if the hydrolysis had not destroyed the sugar molecules. After HCl treatment, only Glucosamine and Muramic acid should be detected and the areas of the peaks should be similar to the areas of the non-hydrolysed Glucosamine and Muramic acid standards because, theoretically, it was injected the same concentration for all of them.

Controls to confirm the purity of the PG

Injection of 1 mM of Ribitol, Glycerol and Mannosamine (HCL and non-HCL treated) to determine whether the PGN was indeed pure or still had WTAs attached.

Controls for quantification of Glucosamine and Muramic acid

Injection of fresh solutions of Glucosamine and Muramic acid, at different concentrations - 1 mM followed by 1/2 dilutions until 31.25 μM. The area of these peaks in the chromatogram were used to plot the calibration curve that if $r^2 \sim 1$, the equation was used for the quantification of Glucosamine and Muramic acid in each PGN sample. Since N-acetylglucosamine and N-acetylmuramic acid are converted into Glucosamine and Muramic acid, respectively, under this hydrolysis conditions, we can control if total sample hydrolysis has occurred. In a total hydrolysis scenario no N-acetylglucosamine and N-acetylmuramic acid should be detectable (Covas et al., 2016).

Quantification of Glucosamine and Muramic acid

The area of each peak corresponding to either Glucosamine or Muramic acid was quantified using the Thermo Scientific Dionex Chromeleon Chromatography Data (CDS) System software and thus the correspondent number of moles was calculated. PGN suspensions to be used in the co-precipitation assays were made in MilliQ H_2O and they were diluted to the same number of moles of Muramic acid of the PGN that was more diluted.

PG-PGRP co-precipitation assays

Co-precipitation or pull-down assays intended to determine the binding affinity of the PGRPs to both pure PG and to live cells, i.e., whole bacteria. The PG pull down assays are quantitative assays performed with the non-fluorescent tagged rPGRPs and analyzed by SDS-PAGE. Due to the non-solubility of the PGN in aqueous solutions, the amount of protein that binds is harvested with it. The bacterial binding assays are qualitative and were performed with the mCherry_PGRPs for microscopy visualization.

For the assays, PG suspensions at 2.89×10^{-1} nmol of Muramic acid/μL of PG were thoroughly mixed and 20 μL were taken and spun down for 3 min at RT, 16.1 x1000 *g*. The supernatants were carefully removed, in particular the NCTC Δ *tagO* who's PGN does not pellet well like the others and hence it is easily aspirated. To the pellets it was added 0.3 mg/mL final concentration of either PGRP-SA or PGRP-LC in a 200 μL reaction volume, filling up the volume with the reaction buffer (20 mM Tris-HCl pH 8.0; 300 mM NaCl or PBS 1X pH 6.0 or Hemo Buffer – 7.3 mM $Na_2SO_4.10H_2O$; 34 mM $NaH_2PO_4.H_2O$; 5 mM $Na_2HPO_4.7H_2O$; 25 mM KCl; 2 mM $CaCl_2$; 14.4 mM $MgCl_2.6H_2O$; pH 6.0-6.2 adjusted with NaOH). The mixes were incubated at 25°C for 30 min, without shaking and then centrifuged for 5 min at RT 0.8 × 1000 *g*. The pellets were washed with 200 μL of reaction buffer and centrifuged for 5 min at RT, 3.2 x1000 *g*. The pellets were finally resuspended in 20 μL of 2X SDS LB and boiled for 5 min. The samples were

centrifuged for 3 min at RT, 16.1 x1000 *g*. The supernatants were recovered (20 μL) into a fresh tube. This collection step allows the loading on the gel to be quick, clean and guarantees that only the supernatant and not bits of PG were loaded, which is important for comparing and quantifying the binding affinities. The full supernatant volume was loaded on a 12% SDS-PAGE gel. Resulting bands were visualized by Coomassie Blue Staining and both imaging and quantification were performed using a Gel Doc^{TM} EZ Gel (Bio-Rad, USA). For quantification purposes of the bindings, each experiment was repeated in two different days, with two different batches of buffers (made fresh).

Live bacteria co-precipitation assays

Bacteria were grown to OD_{600nm} ~0.5 and 1 mL aliquot was centrifuged for 5 min at RT, 16.1 x1000 *g*. The cells were washed with 500 μL of HemoBuffer and centrifuged again. They were resuspended in 0.3 mg/mL final concentration of either mCherry_PGRP-SA or mCherry_PGRP-LC in a 200 μL reaction volume, filling up the volume with Hemo buffer and incubated at RT for 5 min without shaking. The "cells-bound PGRPs" complexes were harvested for 5 min at RT, 7.5 x1000 *g*. The pellets were washed twice with 200 μL of Buffer. Finally, the pellets were resuspended in the left-over volume of the washings and 2 μL was loaded on a 1.2% (w/v) Agarose-PBS slides (it does not dissolve in HemoBuffer). Images were acquired with a GE Healthcare DeltaVision Elite integrated imaging system, in the conventional mode at 25°C using an Olympus 150x 1.45 NA TIRF Objective (Olympus, USA) using the SoftWoRx software (GE Healthcare).

Quantification of binding of fluorescent PGRPs to bacterial cells

This was performed in Image-J. Briefly, a threshold was applied to the phase image to obtain a mask that harbors the cells. Since PGRPs bind exogenously this mask was slightly dilated to encompass the fluorescence signal. The mask was processed by the watershed algorithm to separate the area of cells in close proximity and the resulting mask was used to create a list of Region of Interest (ROI), with each ROI harboring a different object. The list of ROIs was used to measure the fluorescent signal intensity after subtracting the background fluorescence. The average signal intensity of each ROI was used as a measure of the binding intensity.

Survivals and CFUs

Bacterial cultures were grown overnight. Optical density was adjusted with the appropriate medium culture as follows: *B. subtilis* 5.0, *B. subtilis* Δ *tagO* 7.0, *S. aureus* 0.5 and *S. aureus* Δ *tagO* 0.7. 500μL of these cultures was centrifuged at RT for 10min, 3380 x *g*. The cells were washed once with 1mL of PBS (Phosphate-Saline Buffer) and resuspended in 500μL of PBS. 32.2 nL of bacterial suspension was injected in thorax of 3-5-day old flies using a nanoinjector (Nanoject II; Drummond Scientific, Broomall, PA). The infected flies were kept at 25°C and monitored for 72h every 12h. For determination of the CFUs, infected flies were collected at different time-points, homogenized and plated in the appropriate media and incubated at 25°C for 24h-48h. Survival curves and CFUs were determined for at least 3 independent experiments for each infection with (n = 30/infection).

Measurements of gene expression in whole flies

Total RNA was extracted from whole flies (5 females) from three biological repeats upon injection using the Total RNA Purification Plus kit (Norgen Biotek), and cDNA was prepared from 0.5 mg total RNA using the Maxima First Strand cDNA synthesis kit (Thermo Scientific). The reaction conditions involved a step at 25°C for 10 min followed by a 15-minute step at 65°C and ending with a 5 min step at 85°C. QPCR reactions were carried out with the SensiFASAT SYBR No-ROX kit (Bioline) in a Corbet Rotor-Gene 6000 qPCR machine (QIAGEN) using 2 μl of cDNA template diluted tenfold and 400nM of each primer. Expression values were calculated using the DDCt method and normalized to rp49 expression levels. List of primers as follows:

rp49 forward: AAGAAGCGCACCAAGCACTTCATC
rp49 reverse: TCTGTTGTCGATACCCTTGGGCTT
diptericin forward: ACCGCAGTACCCACTCAATC
diptericin reverse: GGTCCACACCTTCTGGTGAC
drosomycin forward: AGTACTTGTTCGCCCTCTTCGCTG
drosomycin reverse: CCTTGTATCTTCCGGACAGGCAGT

Time-lapse microscopy

For microscopy of the GFP::PGRP-LCx binding to live bacterial cells, *w* UAS-GFP::PGRP-LCx virgin females were crossed with Hml$^{\Delta}$Gal4. Primary macrophages from F1 third instar larvae were used as described below. All other videos were done using primary macrophages from Moesin-GFP third instar larvae.

Macrophage preparation

Three third instar larvae were washed in 1 mL of ddH_2O followed by 50% (v/v) bleaching and vortex. The larvae were quickly washed three times with autoclaved MilliQ H_2O and left swimming while the slides were prepared. Into previously washed metal slides (100% (v/v) Ethanol) with a 1.0 mm coverslip it was put Schneider medium supplemented with 5% Fetal Calf Serum. The larvae were bled into the medium and the macrophages were let settle for 1 h at RT in a humid chamber.

Preparation of the bacteria

200 μL of an O/N culture were harvested at RT and washed with of Schneider. The cells were resuspended in 200 μL of Schneider with 0.5 μL of a DAPI solution (at 1 mg/mL in MilliQ H_2O) and incubated for 5 min at RT, without shaking. The cells washed with 200 μL of Schneider and resuspended in 100 μL of Schneider. Finally, they were dilute 1/10 in Schneider in a 200 μL final volume.

Imaging of macrophages-bacteria interaction

To image the interactions between bacteria and macrophages, 200 μL of bacterial suspension was added on macrophages cultures in a "well slide" as previously described (Parton et al., 2010). Briefly, well slides were prepared by gluing a coverslip with SYLGARD 184 Elastomer (Dow Corning) to an aluminum metal slide with a central hole of 1.5cm diameter. Approximately 200 μL of medoum was added to the well, macrophages isolated into this and allowed to settle onto the coverslip in a humidifier chamber. Before mounting on the microscope, an YSI 5775 Standard Membrane (YSI Incorporated, Japan) was placed over the medium to reduce dehydration and improve imaging. This was covered with a humidifier chamber for long-term imaging. Live imaging of UAS-GFP-PGRP-LCx haemocytes was performed at 25°C in a humid chamber for 12 h at 15 min intervals. With the same conditions, live imaging of moesin-GFP hemocytes was performed for 18h at 3min intervals. In all experiments we used a GE Healthcare DeltaVision Elite integrated imaging system and an Olympus MPLAPON-Oil immersion objective (100X 1.40 NA). For high-sensitivity imaging and to allow lower intensities of excitation light, we used the Photometrics Evolve back-thinned EMCCD camera. Time-lapse images were denoised post acquisition using an implementation of the Patch-Based Denoising algorithm (Kervann and Boulanger, 2008). Default parameters were used, except: number of iterations (= 3), adaptability (= 10) and patch radius (= 3).

QUANTIFICATION AND STATISTICAL ANALYSIS

All data was plotted and analyzed using GraphPad Prism 5 (GraphPad Software, Inc.). Statistical significance for CFU data was determined by Student's t test. To determine statistical significance between them, estimated survival probability curves were analyzed using the Log-rank (Mantel-Cox) and Wilcoxon tests.

Cell Reports, Volume *27*

Supplemental Information

Accessibility to Peptidoglycan Is Important for the Recognition of Gram-Positive Bacteria in *Drosophila*

Filipa Vaz, Ilias Kounatidis, Gonçalo Covas, Richard M. Parton, Maria Harkiolaki, Ilan Davis, Sergio Raposo Filipe, and Petros Ligoxygakis

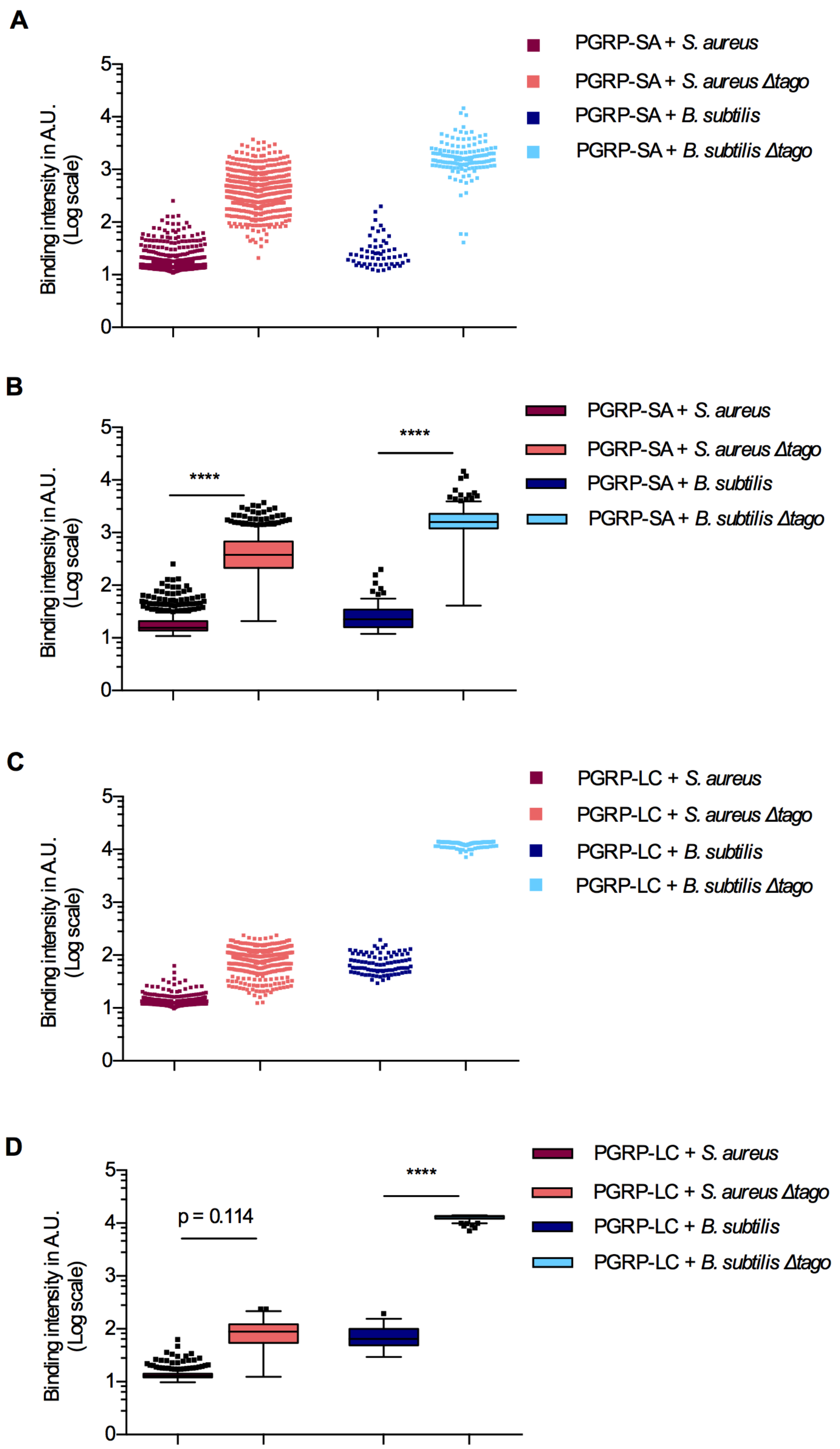
A
Binding intensity in A.U. (Log scale)
0
1
2
3
4
5
PGRP-SA + S. aureus
PGRP-SA + S. aureus Δtago
PGRP-SA + B. subtilis
PGRP-SA + B. subtilis Δtago
B
Binding intensity in A.U. (Log scale)
****
****
PGRP-SA + S. aureus
PGRP-SA + S. aureus Δtago
PGRP-SA + B. subtilis
PGRP-SA + B. subtilis Δtago
C
Binding intensity in A.U. (Log scale)
PGRP-LC + S. aureus
PGRP-LC + S. aureus Δtago
PGRP-LC + B. subtilis
PGRP-LC + B. subtilis Δtago
D
Binding intensity in A.U. (Log scale)
p = 0.114
****
PGRP-LC + S. aureus
PGRP-LC + S. aureus Δtago
PGRP-LC + B. subtilis
PGRP-LC + B. subtilis Δtago

Figure S1. Quantification of PGRP-SA and PGRP-LC binding to *S. aureus, B. bacillus* and their TagO variants. Related to Figures 1 and 2 (A) Scatterplot to indicate all values of mCherry-PGRP-SA binding to whole bacteria **(B)** Box-plot with mean values and 95% confidence intervals; all comparisons with one-way ANOVA (see Table S1). **(C)** Scatterplot to indicate all values of mCherry-PGRP-LCx binding to whole bacteria **(D)** Box-plot with mean values and 95% confidence intervals; all comparisons with one-way ANOVA (see Table S1).

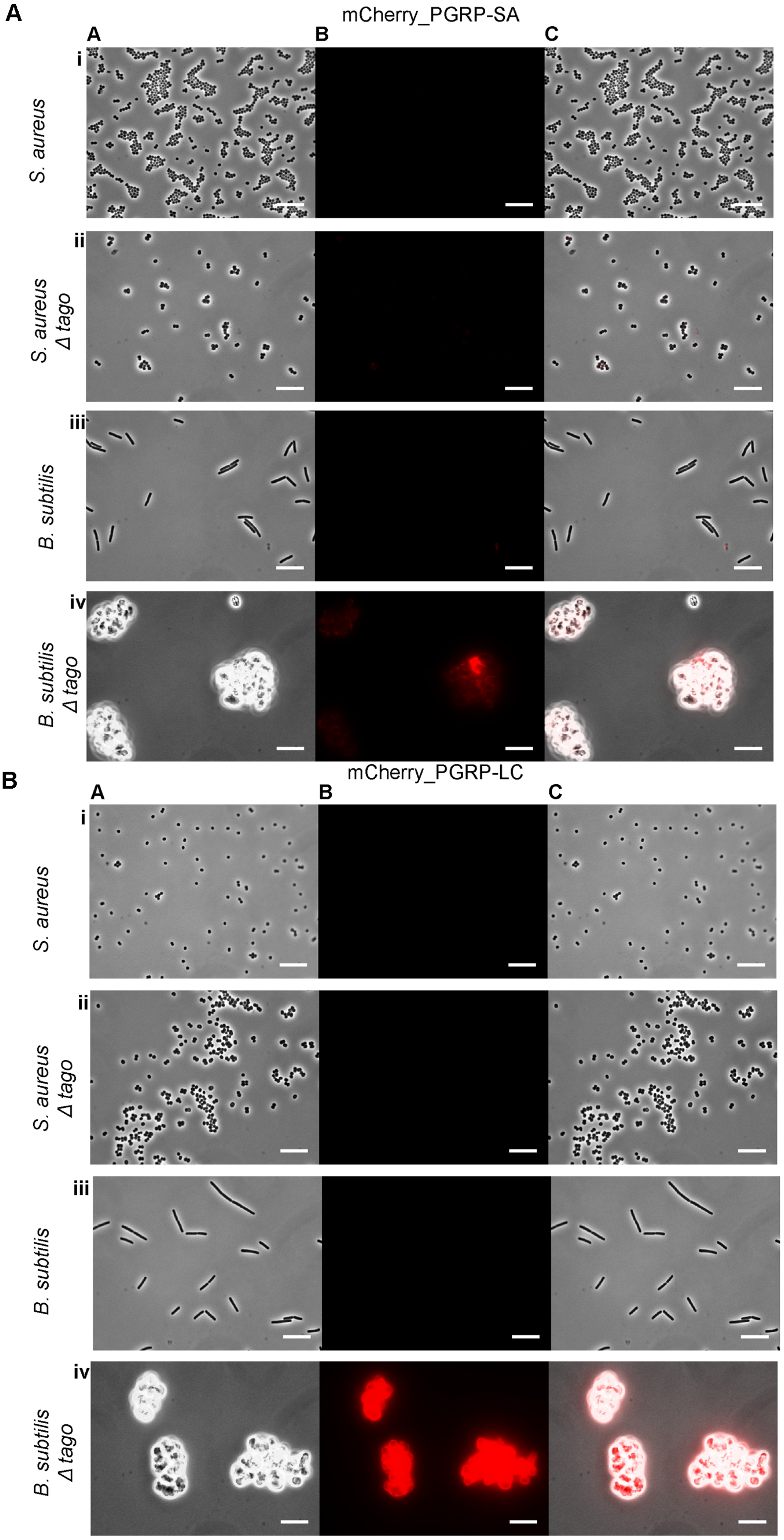
A
mCherry_PGRP-SA
A
B
C
i
S. aureus
ii
S. aureus
Δ tago
iii
B. subtilis
iv
B. subtilis
Δ tago
B
mCherry_PGRP-LC
A
B
C
i
S. aureus
ii
S. aureus
Δ tago
iii
B. subtilis
iv
B. subtilis
Δ tago

Figure S2. Binding of mCherry-PGRP-SA (A) and mPGRP-LC (B) to *S. aureus, B. bacillus* and their TagO mutants with contrast adjusted for easier visual comparison of images with low signal intensities. Related to Figures 1 and 2. (A) Phase contrast **(B)** m-Cherry-PGRP-SA and **(C)** overlay

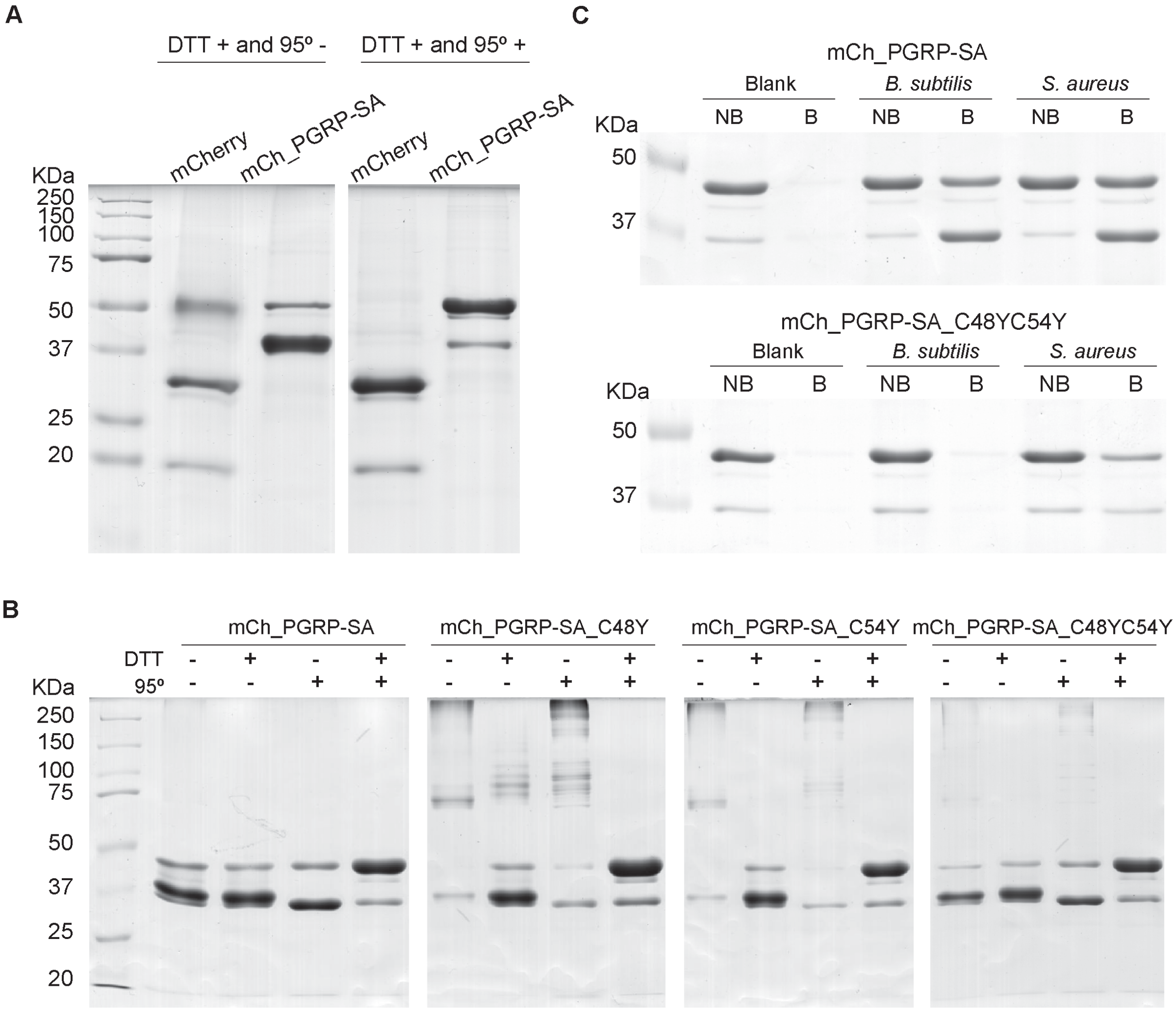
A
DTT + and 95º -
DTT + and 95º +
mCherry
mCh_PGRP-SA
mCherry
mCh_PGRP-SA
KDa
250
150
100
75
50
37
25
20
C
mCh_PGRP-SA
Blank
B. subtilis
S. aureus
KDa
NB
B
NB
B
NB
B
50
37
mCh_PGRP-SA_C48YC54Y
Blank
B. subtilis
S. aureus
KDa
NB
B
NB
B
NB
B
50
37
B
mCh_PGRP-SA
mCh_PGRP-SA_C48Y
mCh_PGRP-SA_C54Y
mCh_PGRP-SA_C48YC54Y
DTT
95º
KDa
250
150
100
75
50
37
25
20

Figure S3. Purified recombinant mCherry_PRGP-SA proteins. Related to Figures 1, 2 and 4. (A) Purified mCherry has an expected size of 29.070 KDa. Under non-fully denaturing conditions (DTT but not heated), mCherry presented a multimer form (ca. 50 KDa), the fully denatured 29 KDa band and a band below due to an internal RBS, which led to a 12aa shorter protein. The lower band, ca. 20 KDa is a degradation product. mCherry_PGRP-SA submitted to the same treatment, presented two bands ca. 50 KDa, which corresponded to the full-length denatured protein (49.701 KDa) and to the 12aa shorter form. The band of ca. 40 KDa is the non-fully denatured form. When heated at 95˚, both proteins showed a stronger band with the expected sizes for the full-length proteins. **(B)** The single mutants C48Y and C54Y had an expected size of 49.77 KDa, while C48YC54Y a size of 49.81 KDa. Both single cysteine mutants presented high number of multimer forms, which suggested that they had folding defects. In contrast, C48YC54Y presented in solution, a similar native conformation as the wild-type protein. **(C)** Purified and quantified PG from *B. subtilis* and *S. aureus* was incubated with mCherry_PGRP-SA and mCherry_PGRP-SA^{C48YC54Y}. After incubation, the complex PG-PGRP was pulled down by low speed centrifugation. NB – Non-bound fraction, i.e. amount of PGRP that did not bind to PG, present in the supernatant after centrifugation. B – Bound fraction, i.e. amount of PGRP that bound to PG and was pulled down after centrifugation (pellet). Blank shows each PGRP incubated in the reaction buffer without PG under the same conditions as the PG-PGRP sample. Both mCherry_PGRP-SA and mCherry_PGRP-SA^{C48YC54Y} were soluble and did not precipitate. The double cysteine mutant however, showed impaired binding ability to both PG types.

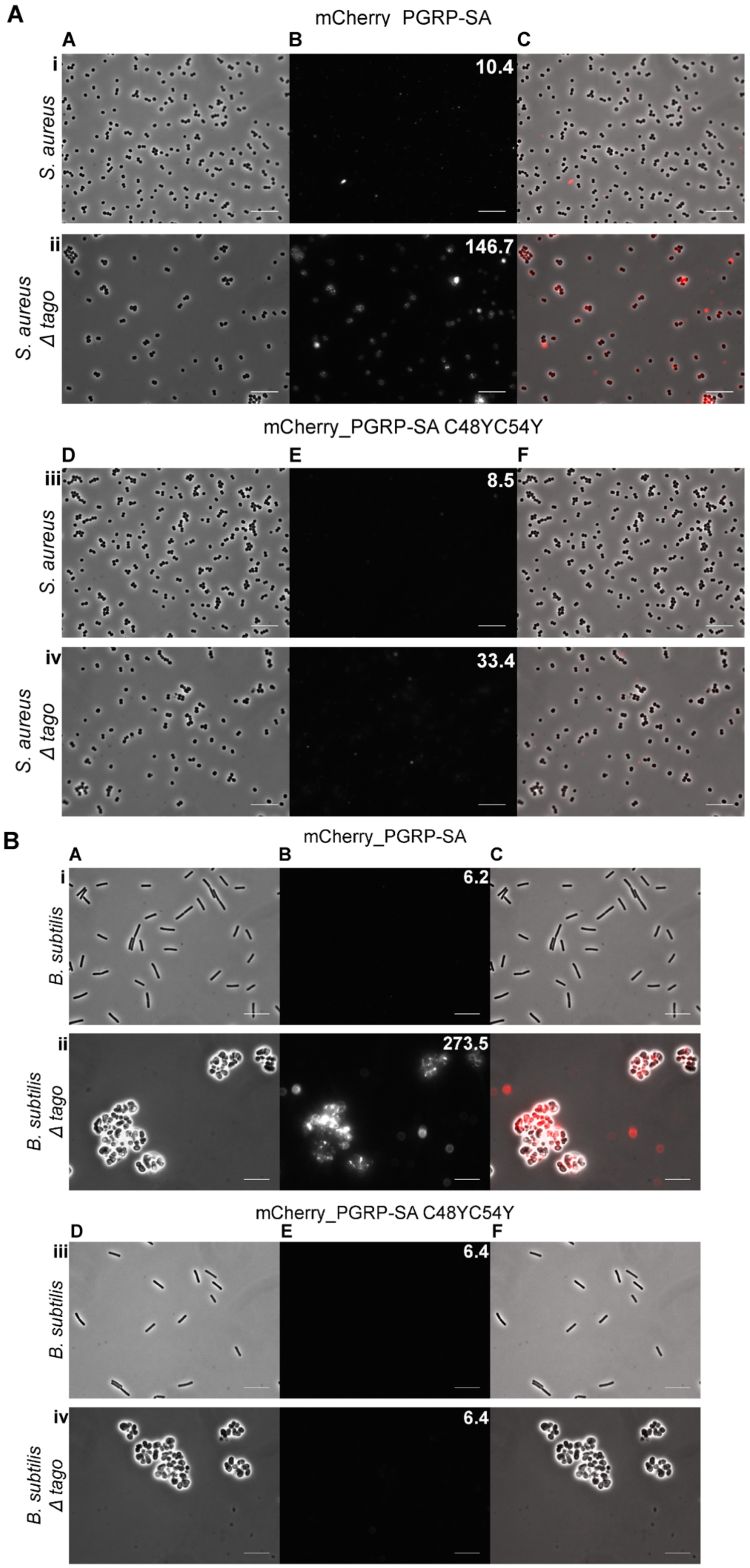
A
mCherry PGRP-SA
A
B
C
i
S. aureus
10.4
ii
S. aureus Δ tago
146.7
mCherry_PGRP-SA C48YC54Y
D
E
F
iii
S. aureus
8.5
iv
S. aureus Δ tago
33.4
B
mCherry_PGRP-SA
A
B
C
i
B. subtilis
6.2
ii
B. subtilis Δ tago
273.5
mCherry_PGRP-SA C48YC54Y
D
E
F
iii
B. subtilis
6.4
iv
B. subtilis Δ tago
6.4

Figure S4. Binding of m-Cherry-PGRP-SA and m-Cherry-PGRP-SA^{C48YC54Y} to *S. aureus* (A) and to *B. subtilis* (B). Related to Figures 1 and 2.

***S. aureus* wild type and *TagO* mutant*:* (A-C).** Binding of a wild type version of a fluorescently tagged PGRP-SA (mCherry_PGRP-SA) to *S. aureus* (i) and *S. aureusΔTagO* (ii). **(A)** Phase contrast channel **(B)** m-Cherry-PGRP-SA and **(C)** overlay. Quantification of binding showed a significant increase in median intensity from 10.4 AU (Bi) to 146.7 AU (Bii). **(D-F).** Binding of a fluorescently tagged mutant PGRP-SA (mCherry_PGRP-SA^{C48YC54Y}) to *S. aureus* (iii) and *S. aureusΔTagO* (iv). **(D)** Phase contrast channel **(E)** m-Cherry-PGRP-SA^{C48YC54Y} and **(F)** overlay. Quantification of binding showed a non-significant increase of median intensity from 8.5 AU (Eiii) to 33.4 AU (Eiv). Exposure time for all 10msec, scale bar 1μm.

***B. subtilis* wild type and *TagO* mutant (A-C).** Binding of a wild type version of a fluorescently tagged PGRP-SA (mCherry_PGRP-SA) to *B. subtilis* (i) and *B. subtilisΔTagO* (ii). **(A)** Phase contrast channel **(B)** m-Cherry-PGRP-SA and **(C)** overlay. Quantification of binding showed a significant increase in median intensity from 6.2 AU (Bi) to 273.5 AU (Bii). **(D-F).** Binding of a mutant version of a mutant fluorescently tagged PGRP-SA (mCherry_PGRP-SA^{C48YC54Y}) to *B. subtilis* (iii) and *B. subtilisΔTagO* (iv). **(D)** DIC channel **(E)** m-Cherry-PGRP-SA^{C48YC54Y} and **(F)** overlay. Quantification of binding showed no increase in median intensity (compare Eiii and Eiv). Exposure time for all 10ms, scale bar 1μm.

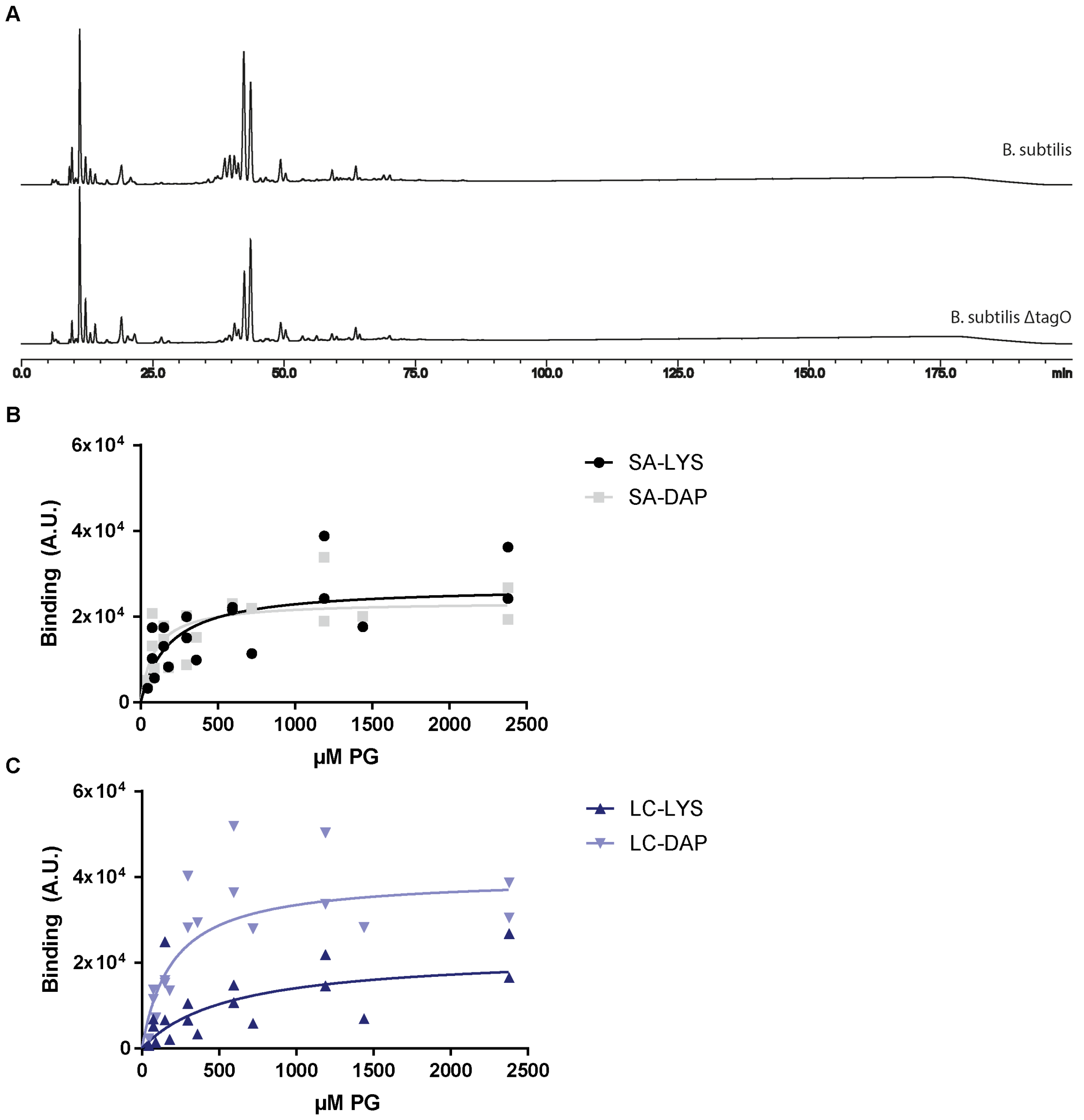

Figure S5. HPLC profiles of *B. subtilisΔTagO* and its parental strain and saturation binding of PG to PGRPs. Related to Figure 4. (A) ***HPLC Profiles***: muropeptide composition of the two *B. subtilis* strains used were identical as seen by HPLC analysis of mutanolysin-digested PG. Therefore, removal of WTA did not affect PG composition.

(B and C) ***Saturation binding experiments:*** Curves of best fit in PG saturation binding experiments where the concentration of each PGRP was kept constant while the amount of quantified PG added to the reaction was increased until no more PGRP precipitated with the addition of the PG ligand. **(B)** There was no significant difference (non-linear regression analysis) between the binding of PGRP-SA to Lys-type and DAP-type PG. **(C)** In contrast, there was significant preference of PGRP-LC for DAP-type PG and comparable to PGRP-SA (but lower) binding to Lys-type PG.

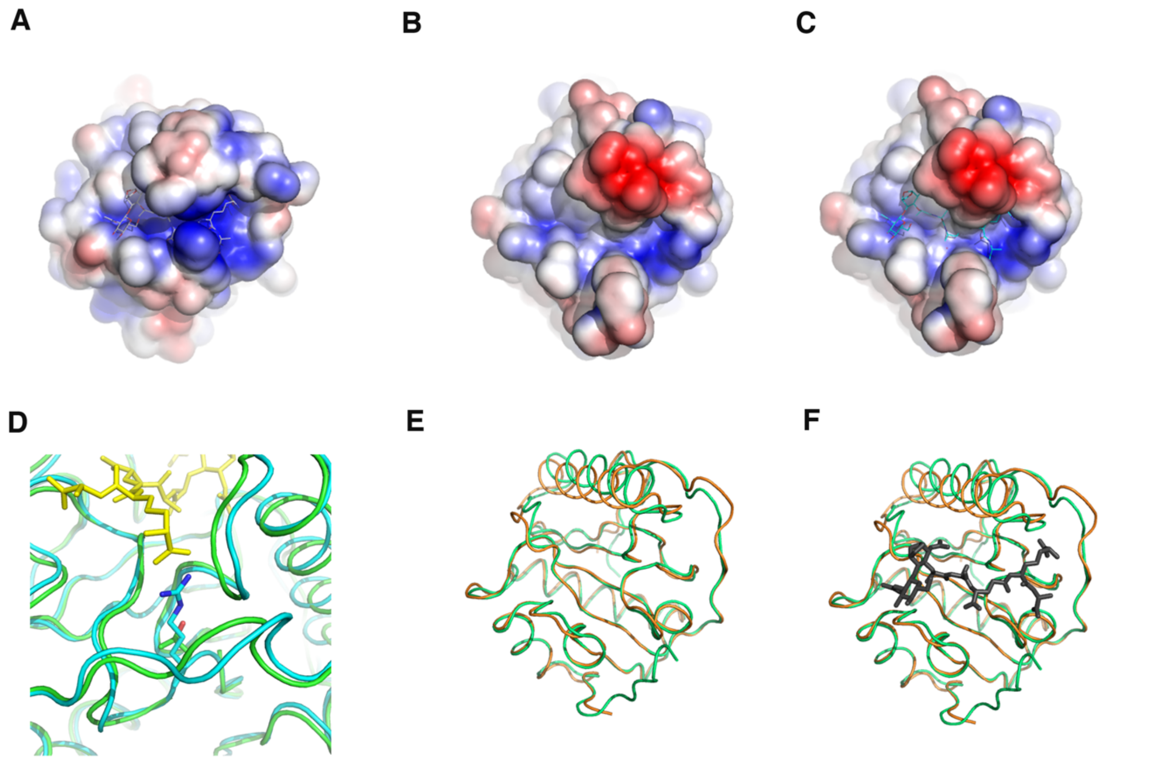

Figure S6. Structural meta-analysis of PGRP-LC and PGRP-SA binding to PG. Related to Figure 4. APBS/Pymol Electrostatic surface representations of: **(A)** PGRP-LC (with TCT in stick representation), **(B)** PGRP-SA, and **(C)** PGRP-SA with the TCT in position as placed by the superposition of the protein backbone of PGRP-LC against PGRP-SA. **(D)** A close-up of TCT bound to PGRP-LC (cyan) with the structure of PGRP-SA (green) superposed; the key Arg413 that docks DAP in PGRP-LC is shown (blue) along with the Threonine (red) that takes its place in PGRP-SA. **(E)** Calpha-defined superposition of PGRP-LC and PGRP-SA and **(F)** the same with TCT bound.

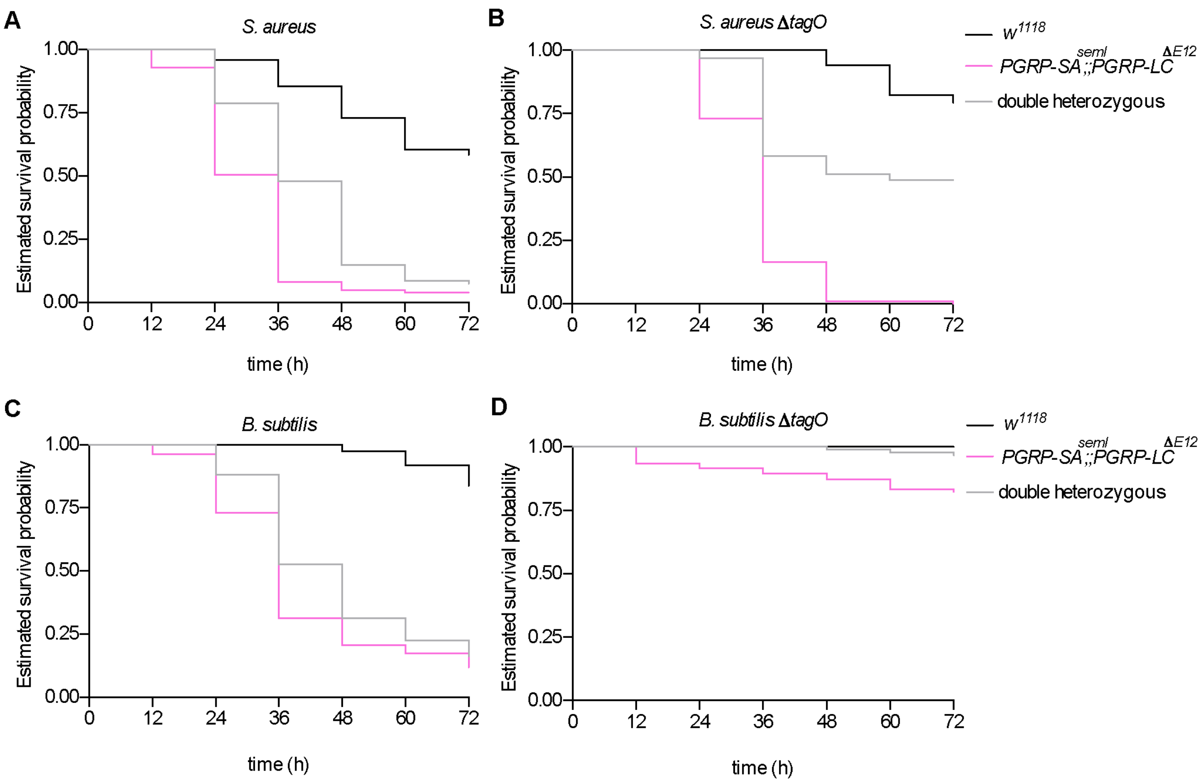

Figure S7. Survival probabilities of controls vs. double *PGRP-SA^{seml};;PGRP-LC$^{\Delta E12}$* homozygous mutants. Related to Figure 5. In all cases namely, after infection with **(A)** *S. aureus* **(B)** *S. aureusΔTagO* **(C)** *B. subtilis* **(D)** *B. subtilisΔTagO, w1118* flies were the most resistant. The double homozygous mutant was the most susceptible while the double heterozygote mutant was significantly different from the double homozygote in all cases and significantly different from wild type in cases A, B and C (stats in Table S3).

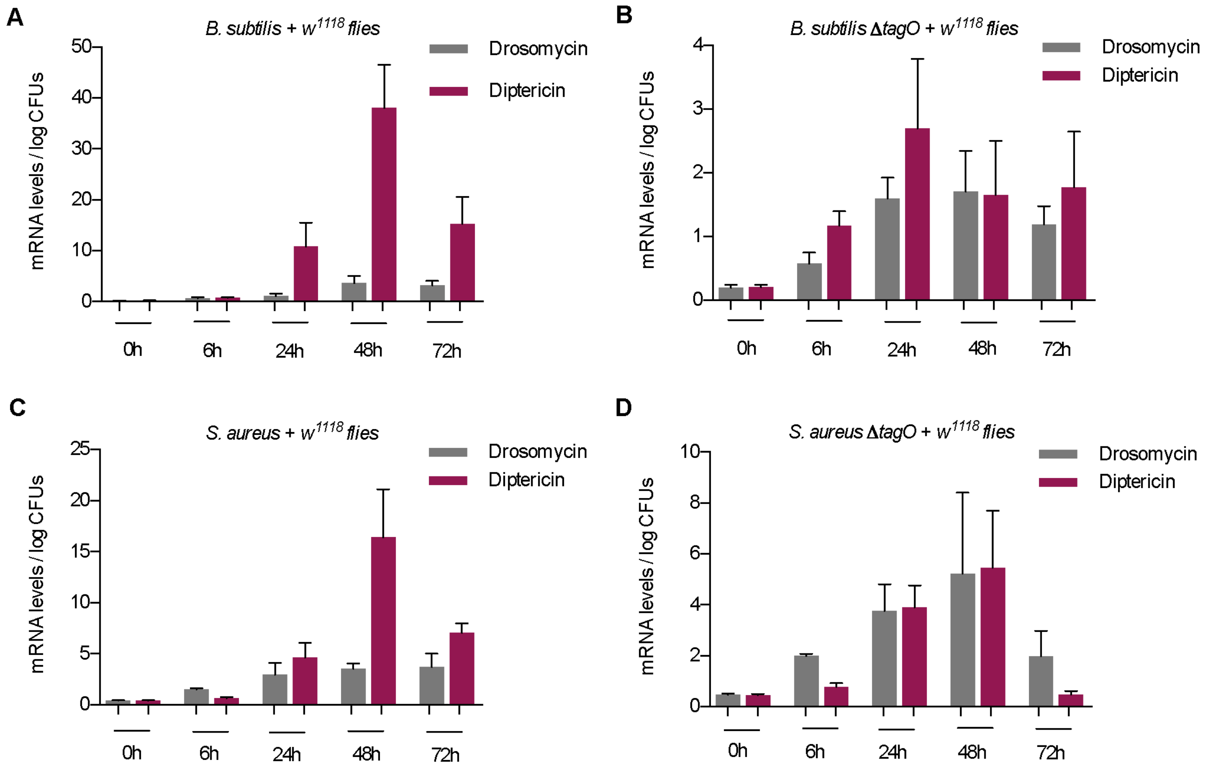

Figure S8. Pattern of AMP induction in w^{1118} flies normalized to CFUs. Related to Figure 7. (A) Infection by *B. subtilis* induced differential AMP activation with significant differences between *dipt* and *drs*. **(B)** These differences disappeared after infection with *B. subtilisΔTagO*. **(C)** Similarly, infection with *S. aureus* induced differential AMP expression that was abolished **(D)** when flies were infected with *S. aureusΔTagO.*

Statistics (SF3 and SF4)	
Ordinary one-way ANOVA	
Number of families	1
Number of comparisons per family	28
Alpha	0.05

Uncorrected Fisher's LSD	Mean Diff.	95% CI of diff.	Significant?	Summary	Individual P Value
PGRP-LC + *B. subtilis* vs. PGRP-LC + *B. subtilis Δtago*	-12690	-12873 to -12507	Yes	****	< 0.0001
PGRP-LC + *B. subtilis* vs. PGRP-LC + *S. aureus*	59.91	-92.49 to 212.3	No	ns	0.4408
PGRP-LC + *B. subtilis* vs. PGRP-LC + *S. aureus Δtago*	-16.07	-171.0 to 138.9	No	ns	0.8389
PGRP-LC + *B. subtilis* vs. PGRP-SA + *B. subtilis*	40.07	-181.2 to 261.4	No	ns	0.7225
PGRP-LC + *B. subtilis* vs. PGRP-SA + *B. subtilis Δtago*	-2058	-2235 to -1881	Yes	****	< 0.0001
PGRP-LC + *B. subtilis* vs. PGRP-SA + *S. aureus*	51.89	-99.80 to 203.6	No	ns	0.5024
PGRP-LC + *B. subtilis* vs. PGRP-SA + *S. aureus Δtago*	-480.4	-630.7 to -330.1	Yes	****	< 0.0001
PGRP-LC + *B. subtilis Δtago* vs. PGRP-LC + *S. aureus*	12750	12614 to 12887	Yes	****	< 0.0001
PGRP-LC + *B. subtilis Δtago* vs. PGRP-LC + *S. aureus Δtago*	12674	12535 to 12814	Yes	****	< 0.0001
PGRP-LC + *B. subtilis Δtago* vs. PGRP-SA + *B. subtilis*	12730	12520 to 12941	Yes	****	< 0.0001
PGRP-LC + *B. subtilis Δtago* vs. PGRP-SA + *B. subtilis Δtago*	10633	10469 to 10796	Yes	****	< 0.0001
PGRP-LC + *B. subtilis Δtago* vs. PGRP-SA + *S. aureus*	12742	12607 to 12878	Yes	****	< 0.0001
PGRP-LC + *B. subtilis Δtago* vs. PGRP-SA + *S. aureus Δtago*	12210	12076 to 12344	Yes	****	< 0.0001
PGRP-LC + *S. aureus* vs. PGRP-LC + *S. aureus Δtago*	-75.98	-171.6 to 19.62	No	ns	0.1192
PGRP-LC + *S. aureus* vs. PGRP-SA + *B. subtilis*	-19.84	-204.5 to 164.9	No	ns	0.8332
PGRP-LC + *S. aureus* vs. PGRP-SA + *B. subtilis Δtago*	-2118	-2246 to -1989	Yes	****	< 0.0001
PGRP-LC + *S. aureus* vs. PGRP-SA + *S. aureus*	-8.02	-98.25 to 82.22	No	ns	0.8617
PGRP-LC + *S. aureus* vs. PGRP-SA + *S. aureus Δtago*	-540.3	-628.2 to -452.5	Yes	****	< 0.0001
PGRP-LC + *S. aureus Δtago* vs. PGRP-SA + *B. subtilis*	56.14	-130.6 to 242.9	No	ns	0.5556
PGRP-LC + *S. aureus Δtago* vs. PGRP-SA + *B. subtilis Δtago*	-2042	-2173 to -1910	Yes	****	< 0.0001
PGRP-LC + *S. aureus Δtago* vs. PGRP-SA + *S. aureus*	67.96	-26.49 to 162.4	No	ns	0.1584
PGRP-LC + *S. aureus Δtago* vs. PGRP-SA + *S. aureus Δtago*	-464.4	-556.6 to -372.2	Yes	****	< 0.0001
PGRP-SA + *B. subtilis* vs. PGRP-SA + *B. subtilis Δtago*	-2098	-2303 to -1892	Yes	****	< 0.0001
PGRP-SA + *B. subtilis* vs. PGRP-SA + *S. aureus*	11.82	-172.3 to 195.9	No	ns	0.8998
PGRP-SA + *B. subtilis* vs. PGRP-SA + *S. aureus Δtago*	-520.5	-703.5 to -337.5	Yes	****	< 0.0001
PGRP-SA + *B. subtilis Δtago* vs. PGRP-SA + *S. aureus*	2110	1982 to 2237	Yes	****	< 0.0001
PGRP-SA + *B. subtilis Δtago* vs. PGRP-SA + *S. aureus Δtago*	1577	1451 to 1703	Yes	****	< 0.0001
PGRP-SA + *S. aureus* vs. PGRP-SA + *S. aureus Δtago*	-532.3	-619.0 to -445.7	Yes	****	< 0.0001

Table S1. Related to Figures 1, 2, S2, S3.

PGRPs							
mCherry_PGRP-SA				mCherry_PGRP-LC			
Lys Type PG		DAP Type PG		Lys Type PG		DAP Type PG	
Kd	Bmax	Kd	Bmax	Kd	Bmax	Kd	Bmax
182.4	27054	88.94	23486	575.9	22152	198.3	40085

Table S2. Binding affinity (Kd) and Maximum binding (Bmax) of mCherry_PGRP-SA and mCherry_PGRP-LC to Lys type and DAP type peptidoglycan. Related to Figure 4 and Figures S5B and S5C. Calculations were made with GraphPad using one site specific binding regression curve. It is based on the Michaelis-Menten enzymology equation {S + E <-> [SE] -> E + P} where S = substrate, E = enzyme, P = product. Based on published structures (see Fig. S6 above), we assume that PGRP-SA and PGRP-LC have only one binding site for PG, which means that 1 PGRP molecule binds to PG only once.

Statistics for Figure 5A								
Log-rank test	w^{1118} vs *PGRP-LC*$^{\Delta E12}$	w^{1118} vs *PGRP-SA*	w^{1118} vs *PGRP-SA;;PGRP-LC*$^{\Delta E12}$	w^{1118} vs double hetero	*PGRP-LC*$^{\Delta E12}$ vs *PGRP-SA*	*PGRP-SA* vs *PGRP-SA;;PGRP-LC*$^{\Delta E12}$	*PGRP-LC*$^{\Delta E12}$ vs *PGRP-SA;;PGRP-LC*$^{\Delta E12}$	*PGRP-SA;;PGRP-LC*$^{\Delta E12}$ *vs double hetero*
Chi square	3.908	85.99	79.19	45.5	64.36	7.402	69.55	30.87
df	1	1	1	1	1	1	1	1
P value	0.0481	< 0.0001	< 0.0001	< 0.0001	< 0.0001	0.0065	< 0.0001	< 0.0001
P value summary	*	****	****	****	****	**	****	****
Are the survival curves sig different?	Yes	Yes	Yes	Yes	Yes	Yes	Yes	Yes

Statistics for Figure 5B								
Log-rank test	w^{1118} vs *PGRP-LC*$^{\Delta E12}$	w^{1118} vs *PGRP-SA*	w^{1118} vs *PGRP-SA;;PGRP-LC*$^{\Delta E12}$	w^{1118} vs double hetero	*PGRP-LC*$^{\Delta E12}$ vs *PGRP-SA*	*PGRP-SA* vs *PGRP-SA;;PGRP-LC*$^{\Delta E12}$	*PGRP-LC*$^{\Delta E12}$ vs *PGRP-SA;;PGRP-LC*$^{\Delta E12}$	*PGRP-SA;;PGRP-LC*$^{\Delta E12}$ *vs double hetero*
Chi square	4.004	84.25	97.69	10.45	82.49	15.82	108	74.96
df	1	1	1	1	1	1	1	1
P value	0.0454	< 0.0001	< 0.0001	0.0012	< 0.0001	< 0.0001	< 0.0001	< 0.0001
P value summary	*	****	****	**	****	****	****	****
Are the survival curves sig different?	Yes	Yes	Yes	Yes	Yes	Yes	Yes	Yes

Statistics for Figure 5C								
Log-rank test	w^{1118} vs *PGRP-LC*$^{\Delta E12}$	w^{1118} vs *PGRP-SA*	w^{1118} vs *PGRP-SA;;PGRP-LC*$^{\Delta E12}$	w^{1118} vs double hetero	*PGRP-LC*$^{\Delta E12}$ vs *PGRP-SA*	*PGRP-SA* vs *PGRP-SA;;PGRP-LC*$^{\Delta E12}$	*PGRP-LC*$^{\Delta E12}$ vs *PGRP-SA;;PGRP-LC*$^{\Delta E12}$	*PGRP-SA;;PGRP-LC*$^{\Delta E12}$ *vs double hetero*
Chi square	24.48	56.36	66.87	50.22	11.05	8.178	25.7	25.7
df	1	1	1	1	1	1	1	1
P value	< 0.0001	< 0.0001	< 0.0001	< 0.0001	0.0009	0.0042	< 0.0001	< 0.0001
P value summary	****	****	****	****	***	**	****	****
Are the survival curves sig different?	Yes	Yes	Yes	Yes	Yes	Yes	Yes	Yes

Statistics for Figure 5D								
Log-rank test	w^{1118} vs *PGRP-LC*$^{\Delta E12}$	w^{1118} vs *PGRP-SA*	w^{1118} vs *PGRP-SA;;PGRP-LC*$^{\Delta E12}$	w^{1118} vs double hetero	*PGRP-LC*$^{\Delta E12}$ vs *PGRP-SA*	*PGRP-SA* vs *PGRP-SA;;PGRP-LC*$^{\Delta E12}$	*PGRP-LC*$^{\Delta E12}$ vs *PGRP-SA;;PGRP-LC*$^{\Delta E12}$	*PGRP-SA;;PGRP-LC*$^{\Delta E12}$ *vs double hetero*
Chi square	0.7735	1.173	6.915	1.18	0.255	20.45	13.11	11.04
df	1	1	1	1	1	1	1	1
P value	0.3791	0.2788	0.0085	0.2774	0.6136	< 0.0001	0.0003	0.0009
P value summary	ns	ns	**	ns	ns	****	***	***
Are the survival curves sig different?	No	No	Yes	No	No	Yes	Yes	Yes

Table S3. Statistics of survival probabilities (log-rank test) related to Fig 5.

Statistics for Figure 5A

Gehan-Breslow-Wilcoxon test	w^{1118} vs *PGRP-LC*$^{\Delta E12}$	w^{1118} vs *PGRP-SA*	w^{1118} vs *PGRP-SA;;PGRP-LC*$^{\Delta E12}$	w^{1118} vs double hetero	*PGRP-LC*$^{\Delta E12}$ vs *PGRP-SA*	*PGRP-SA* vs *PGRP-SA;;PGRP-LC*$^{\Delta E12}$	*PGRP-LC*$^{\Delta E12}$ vs *PGRP-SA;;PGRP-LC*$^{\Delta E12}$	*PGRP-SA;;PGRP-LC*$^{\Delta E12}$ *vs double hetero*
Chi square	4.424	69.37	72.42	39.15	40.52	13.66	57.77	37.81
df	1	1	1	1	1	1	1	1
P value	0.0427	< 0.0001	< 0.0001	< 0.0001	< 0.0001	0.0002	< 0.0001	< 0.0001
P value summary	*	****	****	****	****	***	****	****
Are the survival curves sig different?	Yes	Yes	Yes	Yes	Yes	Yes	Yes	Yes

Statistics for Figure 5B

Gehan-Breslow-Wilcoxon test	w^{1118} vs *PGRP-LC*$^{\Delta E12}$	w^{1118} vs *PGRP-SA*	w^{1118} vs *PGRP-SA;;PGRP-LC*$^{\Delta E12}$	w^{1118} vs double hetero	*PGRP-LC*$^{\Delta E12}$ vs *PGRP-SA*	*PGRP-SA* vs *PGRP-SA;;PGRP-LC*$^{\Delta E12}$	*PGRP-LC*$^{\Delta E12}$ vs *PGRP-SA;;PGRP-LC*$^{\Delta E12}$	*PGRP-SA;;PGRP-LC*$^{\Delta E12}$ *vs double hetero*
Chi square	5.442	69.12	75.96	14.36	57.46	15.91	85.11	58.24
df	1	1	1	1	1	1	1	1
P value	0.0197	< 0.0001	< 0.0001	0.0002	< 0.0001	< 0.0001	< 0.0001	< 0.0001
P value summary	*	****	****	***	****	****	****	****
Are the survival curves sig different?	Yes	Yes	Yes	Yes	Yes	Yes	Yes	Yes

Statistics for Figure 5C

Gehan-Breslow-Wilcoxon test	w^{1118} vs *PGRP-LC*$^{\Delta E12}$	w^{1118} vs *PGRP-SA*	w^{1118} vs *PGRP-SA;;PGRP-LC*$^{\Delta E12}$	w^{1118} vs double hetero	*PGRP-LC*$^{\Delta E12}$ vs *PGRP-SA*	*PGRP-SA* vs *PGRP-SA;;PGRP-LC*$^{\Delta E12}$	*PGRP-LC*$^{\Delta E12}$ vs *PGRP-SA;;PGRP-LC*$^{\Delta E12}$	*PGRP-SA;;PGRP-LC*$^{\Delta E12}$ *vs double hetero*
Chi square	26.4	54.24	61	47.78	8.374	14.1	25.34	11.84
df	1	1	1	1	1	1	1	1
P value	< 0.0001	< 0.0001	< 0.0001	< 0.0001	0.0038	0.0002	< 0.0001	0.0006
P value summary	****	****	****	****	**	***	****	***
Are the survival curves sig different?	Yes	Yes	Yes	Yes	Yes	Yes	Yes	Yes

Statistics for Figure 5D

Gehan-Breslow-Wilcoxon test	w^{1118} vs *PGRP-LC*$^{\Delta E12}$	w^{1118} vs *PGRP-SA*	w^{1118} vs *PGRP-SA;;PGRP-LC*$^{\Delta E12}$	w^{1118} vs double hetero	*PGRP-LC*$^{\Delta E12}$ vs *PGRP-SA*	*PGRP-SA* vs *PGRP-SA;;PGRP-LC*$^{\Delta E12}$	*PGRP-LC*$^{\Delta E12}$ vs *PGRP-SA;;PGRP-LC*$^{\Delta E12}$	*PGRP-SA;;PGRP-LC*$^{\Delta E12}$ *vs double hetero*
Chi square	0.7324	0.994	6.551	1.01	0.325	17.672	8.75	9.52
df	1	1	1	1	1	1	1	1
P value	0.3851	0.3121	0.0094	0.3192	0.7127	< 0.0001	0.0008	0.0023
P value summary	ns	ns	**	ns	ns	***	**	**
Are the survival curves sig different?	No	No	Yes	No	No	Yes	Yes	Yes

Table S4. Statistics of survival probabilities (Gehan-Breslow-Wilcoxon test) related to Fig 5.
